# Cerebral small vessel disease and risk of incident stroke, dementia and depression, and all-cause mortality: A systematic review and meta-analysis

**DOI:** 10.1016/j.neubiorev.2018.04.003

**Published:** 2018-04-13

**Authors:** Sytze P. Rensma, Thomas T. van Sloten, Lenore J. Launer, Coen D.A. Stehouwer

**Affiliations:** a CARIM School for Cardiovascular Diseases, Maastricht University Medical Centre+, PO Box 616, 6200 MD, Maastricht, The Netherlands; b Department of Internal Medicine, Maastricht University Medical Centre+, P. Debyelaan 25, 6229 HX, Maastricht, The Netherlands; c Intramural Research Program, Laboratory of Epidemiology and Population Sciences, National Institute on Aging, National Institutes of Health, 7201 Wisconsin Avenue, Bethesda, MD, USA

**Keywords:** Cerebral small vessel disease, White matter hyperintensities, Lacunes, Microbleeds, Perivascular spaces, Cerebral atrophy, Stroke, Dementia, Depression, Mortality, Systematic review, Meta-analysis

## Abstract

MRI features of cerebral small vessel disease (CSVD), i.e. white matter hyperintensities, lacunes, microbleeds, perivascular spaces, and cerebral atrophy, may be associated with clinical events, but the strength of these associations remains unclear. We conducted a systematic review and meta-analysis on the association between these features and incident ischaemic and haemorrhagic stroke, all-cause dementia and depression, and all-cause mortality. For the association with stroke, 36 studies were identified (number of individuals/events [n] = 38,432/4,136), for dementia 28 (n = 16,458/1,709), for depression nine (n = 9,538/1,746), and for mortality 28 (n = 23,031/2,558). Only two studies evaluated perivascular spaces; these results were not pooled. Pooled analyses showed that all other features were associated with all outcomes (hazard ratios ranged 1.22–2.72). Combinations of two features were more strongly associated with stroke than any individual feature. Individual features and combinations of CSVD features are strongly associated with incident ischaemic and haemorrhagic stroke, all-cause dementia and depression, and all-cause mortality. If these associations are causal, the strength of these associations suggests that a substantial burden of disease is attributable to CSVD.

## 1. Introduction

Cerebral small vessel disease (CSVD) features include white matter hyperintensities (WMHs) and lacunes of presumed vascular origin, cerebral microbleeds (CMBs), perivascular spaces, and total cerebral atrophy.(Wardlaw et al., 2013) These features are related to ageing and vascular risk factors,(Pantoni, 2010) and are highly prevalent. (Wardlaw et al., 2013) CSVD has been suggested to be an important source of morbidity associated with ischaemic and haemorrhagic stroke, dementia, and depression,(Pantoni, 2010) and CSVD may increase mortality risk.(Pantoni, 2010)

However, systematic evidence for the importance of MRI CSVD features is limited. For instance, no meta-analysis for incident stroke, dementia, depression, or mortality has been conducted for lacunes, perivascular spaces, or total cerebral atrophy. Two meta-analyses (Charidimou et al., 2018; Debette and Markus, 2010) have examined the association of only WMHs(Debette and Markus, 2010) or CMBs (Charidimou et al., 2018) and incident stroke and dementia, and mortality. Of two meta-analyses(van Agtmaal et al., 2017; Wang et al., 2014) on WMHs and incident depression, only one(van Agtmaal et al., 2017) found an association. Three other meta-analyses(Charidimou et al., 2017; Charidimou et al., 2013; Wang et al., 2015) have examined the association between CMBs and incident stroke. However, these studies included only individuals with prior stroke,(Charidimou et al., 2017; Charidimou et al., 2013; Wang et al., 2015) and evaluated only haemorrhagic stroke.(Charidimou et al., 2017; Wang et al., 2015) Finally, no meta-analysis has evaluated the predictive value of presence of combined CSVD features.

We did a systematic review and meta-analysis on the association between CSVD features, including WMHs, lacunes, CMBs, perivascular spaces, and total cerebral atrophy, and incident ischaemic and haemorrhagic stroke, all-cause dementia and depression, and all-cause mortality. Additionally, we investigated the effect of two or more individual CSVD features combined on any of these outcomes.

## 2. Methods

This review was prepared according to the meta-analyses of observational studies in epidemiology (MOOSE) checklist (Appendix A in Supplementary material).(Stroup et al., 2000) This protocol was published in PROSPERO (CRD42016038521) (Appendix B in Supplementary material).(Booth et al., 2011)

### 2.1. Evaluation procedure

Two independent investigators (SR and TVS) selected all relevant studies based on title and abstract, retrieved selected full texts, performed eligibility assessments, extracted data, and assessed risk of bias. Disagreement between the reviewers was resolved by consensus. A third independent reviewer (CS) solved any persisting disagreements.

### 2.2. Information sources and search

We identified relevant studies through a search of MEDLINE and Embase, from inception to March 2017 (for search terms see Tables S1.1-S1.4). We applied no language restrictions. We hand-searched reference lists of eligible studies and related meta-analyses to identify further relevant studies.

### 2.3. Eligibility criteria and study selection

We included prospective cohort studies in adults (with and without a history of stroke or depression) that evaluated the association between baseline MRI features of CSVD and incident ischaemic or haemorrhagic stroke, dementia or depression, or all-cause mortality. For CSVD, we included WMHs and lacunes of presumed vascular origin, CMBs, perivascular spaces, and total cerebral atrophy.(Wardlaw et al., 2013) Studies were also included when they did not specifically assess lacunes but did assess subcortical infarcts (infarcts in the deep brain region not extending into the cortex) and silent infarcts (infarcts detected in individuals without prior stroke), which include lacunes. (Wardlaw et al., 2013) We excluded studies with a sample size ≤ 50, a mean follow-up < 12 months, or including only CSVD occurring in long-term inflammatory or neurodegenerative conditions (e.g. multiple sclerosis or Parkinson’s disease). In the case of multiple publications from the same cohort, we included the most up-to-date or comprehensive information.

### 2.4. Data collection process

We used a predesigned extraction form to collect information on the following items: study size; follow-up duration; age; sex; prior stroke; baseline cognitive performance; prior depression; MRI characteristics; definitions of CSVD features; outcome definitions; number of events; statistical analysis used; reported risk estimates; and variables adjusted for in the analyses. Any relevant missing information was requested from corresponding authors.

### 2.5. Risk of bias assessment

We evaluated risk of bias with the Newcastle-Ottawa scale (NOS) (Appendix C in Supplementary material).

### 2.6. Variable definition

We used definitions of CSVD features and outcomes as reported in the original published papers. Incident stroke included fatal and nonfatal cerebral infarction and intracerebral haemorrhage. Incident dementia included Alzheimer’s disease, presumed vascular dementia and dementia not further specified. Incident depression subtype was not specified by any of the included studies. For stroke and depression, we included both first and recurrent events.

### 2.7. Statistical analysis

We pooled results for each CSVD feature when ≥ three studies were available with the same outcome. We pooled hazard ratios (HRs) using the random effects inverse variance method. We included the fully adjusted HR (but without adjustments for other CSVD features) (if available). HRs were reported by 49 studies, whereas fifteen studies reported results as odds ratios or relative risks (Table S3); these were treated as HRs.

For WMHs, we separately pooled dichotomous and continuous measures. For dichotomous measures, we compared the HR for a higher vs. a lower category. When ≥ two categories for WMHs were present, we selected the two categories with the highest number of participants and events. For total cerebral atrophy, we pooled only studies using a continuous scale (as percentage total intracranial volume or raw volume), as there were few data with atrophy measured dichotomously. We standardized continuous measures per standard deviation. For studies that reported only deep or periventricular WMHs instead of total WMHs, we included the results for periventricular WMHs in the main analysis, because periventricular WMHs more closely represent total WMHs.(Prasad et al., 2011) Similarly, for studies that reported only deep or lobar CMBs instead of total CMBs, we included the results for deep CMBs in the main analysis, because deep CMBs more closely represent total CMBs(Charidimou et al., 2013) and are more strongly related to hypertension.(Wardlaw et al., 2013) For analyses with combined presence of ≥ two individual CSVD features as the determinant, we pooled HRs for any combination of individual features.

We evaluated the level of statistical heterogeneity across pooled studies per CSVD feature using the I^2^ test.(Higgins and Thompson, 2002) High statistical heterogeneity was defined as I^2^ > 60%. We assessed potential publication bias using funnel plots and, when ≥ ten studies were included in the analysis, by Egger’s test. We corrected for the potential effect of significant funnel plot asymmetry using the trim and fill approach.(Duval and Tweedie, 2000)

We did several pre-specified sensitivity analyses. We repeated analyses by subtype of disease (ischaemic or haemorrhagic stroke, and Alzheimer’s disease or presumed vascular dementia); using only population-based cohort studies; using only studies with high-risk populations (e.g. individuals with prior stroke or other cardiovascular disease, mild cognitive impairment, prior depression, or chronic kidney disease); including only high-quality studies (defined as NOS score > four); using only studies that measured WMHs on an observer-rated semi-quantitative scale; using only studies that measured WMHs on an automated quantitative scale; replacing the results for periventricular WMHs with those for deep WMHs; replacing the risk estimates for deep CMBs with those for lobar CMBs; and replacing adjusted risk estimates with unadjusted risk estimates. In addition, we did several post hoc analyses, including repeating the analyses using only studies that included individuals with prior stroke. Other post hoc analyses are described in the supplemental material (Table S2).

Analyses were done with Review Manager 5.3 and R 3.2.3.

## 3. Results

### 3.1. Selection process and study characteristics

**Fig. 1:**
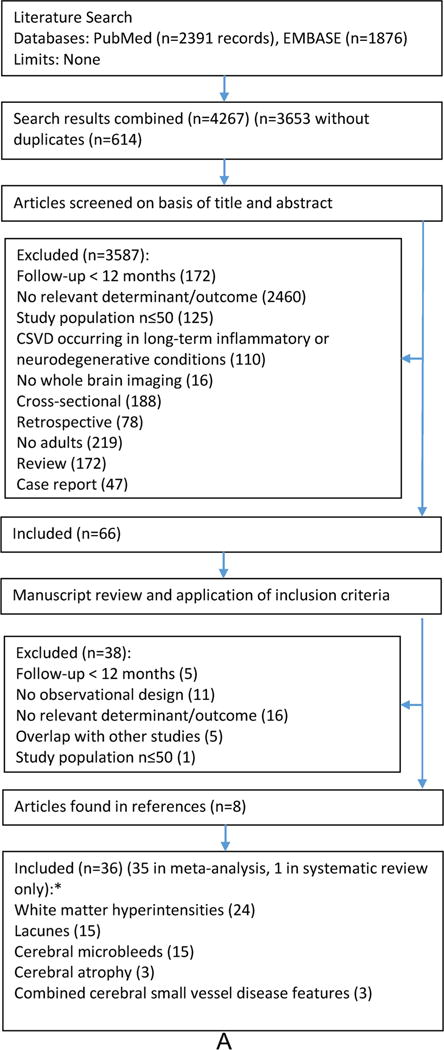

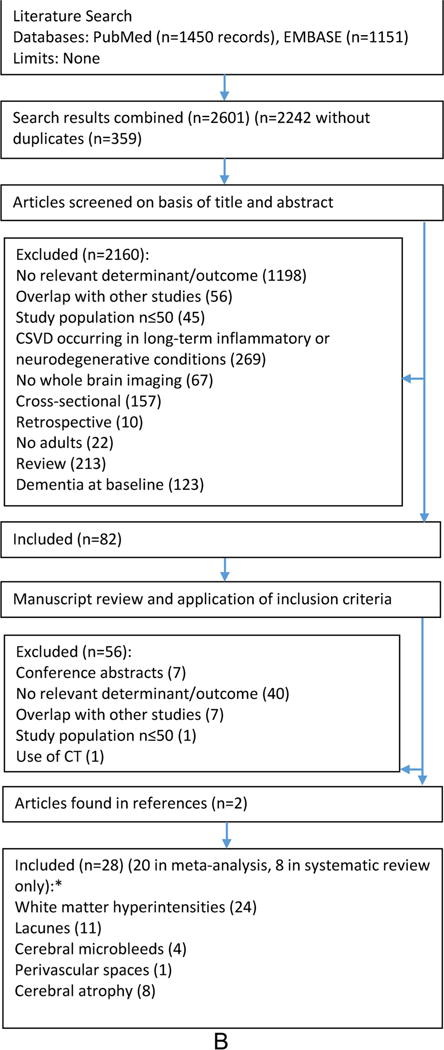

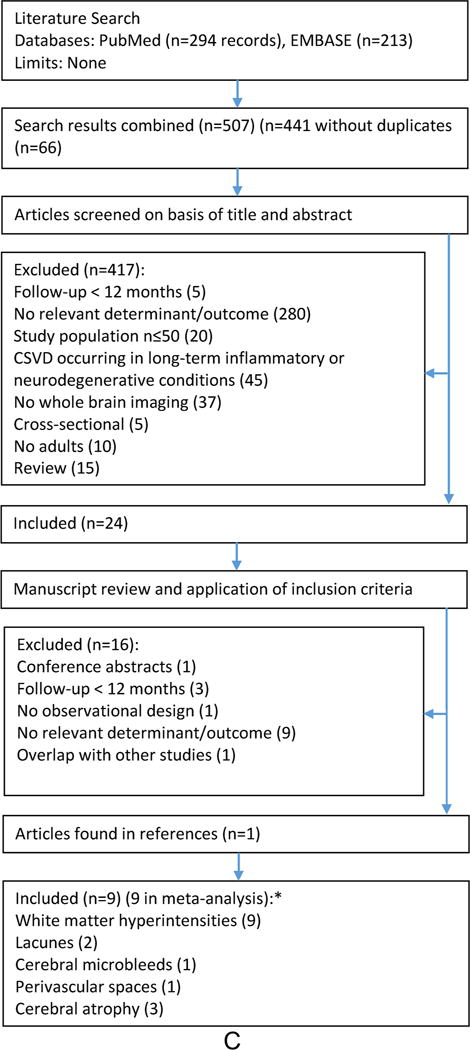

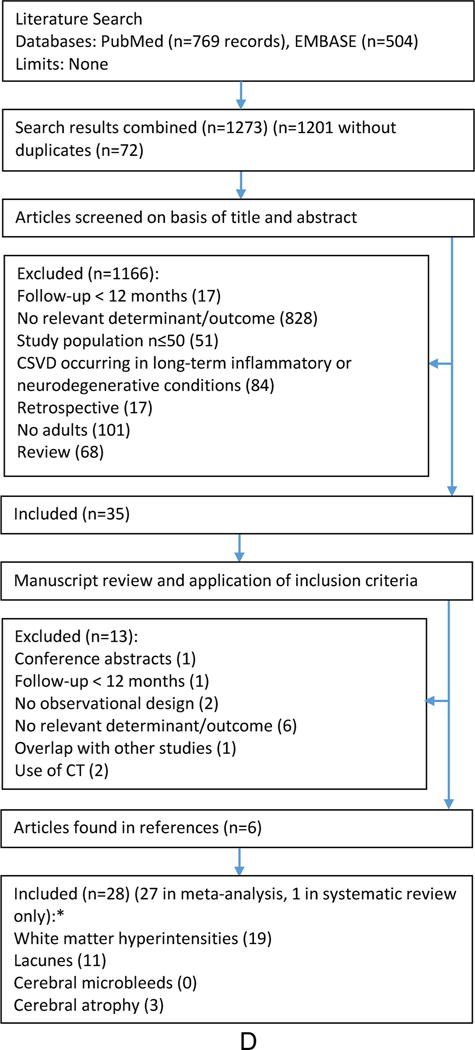
Flow chart of selection procedures for ischaemic and haemorrhagic stroke (panel A), all-cause dementia (B), depression (C) and all-cause mortality (D). *Some studies evaluated multiple markers of cerebral small vessel disease.

Fig. 1 shows the selection process of included studies. In the systematic review, we included 36 studies for ischaemic or haemorrhagic stroke (n = 38,432/4,136 individuals/events), 28 for all-cause dementia (n = 16,458/1,709), nine for depression (n = 9,538/1,746), and 28 for all-cause mortality (n = 23,031/2,558). Table S3 shows the number of included studies in the pooled analyses and per CSVD feature. Only two studies evaluated perivascular spaces; these results were not pooled (Table S3). Full study characteristics and NOS scores are provided as supplemental material (Tables S4.1-S4.4 and S5.1-S5.4, respectively).

### 3.2. Stroke

**Fig. 2:**
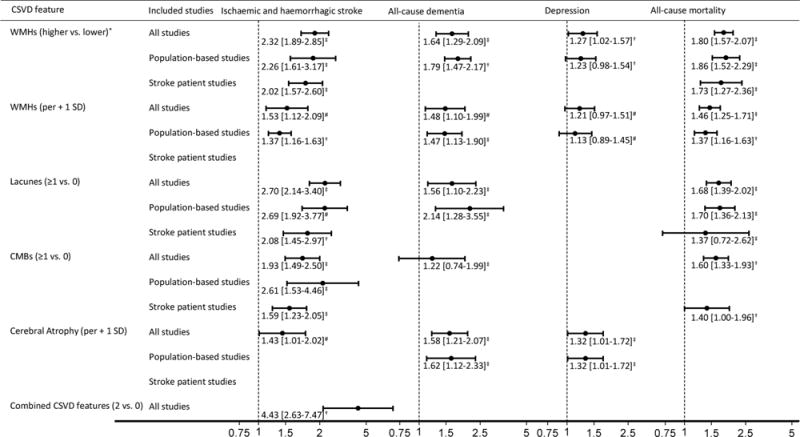
Pooled hazard ratios (95% confidence intervals) for the association between MRI features of cerebral small vessel disease (CSVD) and incident ischaemic and haemorrhagic stroke, all-cause dementia and depression, and all-cause mortality. *Higher vs. lower as defined by the individual studies. ^†^Heterogeneity, I^2^ < 30%, ^‡^I^2^ = 30–60%, ^#^I^2^ > 60%. For the forest plots of each pooled analysis, see supplemental material, Figures S1.1-S1.4. Abbreviations: WMHs = white matter hyperintensities; SD = standard deviation

Fig. 2 shows the pooled hazard ratios and corresponding 95% confidence intervals for the association between CSVD features and clinical events. Results are given for all included studies, and (if available) separately for population-based studies and studies done among individuals with prior stroke. All CSVD features were statistically significantly associated with incident ischaemic or haemorrhagic stroke, with moderate to substantial heterogeneity across studies pooled per CSVD feature (Fig. 2, Figures S1.1a-S1.1e). Of all included studies, 95% found a positive association between any feature and stroke (Figures S1.1a-S1.1e). One study in the systematic review could not be included in the pooled analysis because no risk estimates were presented (Table S3). This study reported a statistically significant association between WMHs and stroke.

For analyses with stroke subtype as the outcome (ischaemic or haemorrhagic), sufficient studies were available for WMHs on a dichotomous scale (14 studies) and CMBs (10 studies) (Table S3). For WMHs, the HRs were qualitatively similar for the risk of ischaemic and haemorrhagic stroke (Figure S2.1). For CMBs, the risk of haemorrhagic stroke was higher than that of ischaemic stroke (Figure S2.4).

### 3.3. Dementia

All features, except CMBs, were statistically significantly associated with incident all-cause dementia, with moderate to substantial heterogeneity across studies pooled per CSVD feature (Fig. 2, Figures S1.2a-S1.2e). Of all included studies, 80% found a positive association between any feature and dementia (Figures S1.2a-S1.2e). Twelve studies were excluded from the pooled analysis (Table S3), of which six (four on WMHs, one on perivascular spaces and one on total cerebral atrophy) found a statistically significant association with dementia.

For analyses with dementia subtype as the outcome (Alzheimer’s disease or presumed vascular dementia), sufficient studies were available for WMHs on a dichotomous scale (nine studies) (Table S3). For WMHs, the risk of presumed vascular dementia was higher than that of Alzheimer’s disease (Figure S2.1).

### 3.4. Depression

WMHs on a dichotomous scale and total cerebral atrophy were statistically significantly associated with incident depression, with low to substantial heterogeneity across studies pooled per CSVD feature (Fig. 2, Figures S1.3a-S1.3c). Of all included studies, 94% found a positive association between any feature and depression (Figures S1.3a-S1.3c). Four studies were excluded from the pooled analysis (Table S3), of which one, on lacunes, found a statistically significant association with depression.

### 3.5. All-cause mortality

WMHs, lacunes and CMBs were statistically significantly associated with all-cause mortality, with low to moderate heterogeneity across studies pooled per CSVD feature (Fig. 2, Figures S1.4a-S1.4d). Among studies done in individuals with prior stroke, lacunes were not statistically significantly associated with all-cause mortality. Of all included studies, 94% found a positive association between any feature and all- cause mortality (Figures S1.4a-S1.4d). All four studies that were excluded from the pooled analysis (Table S3) showed a statistically significant association with all-cause mortality.

### 3.6. Combinations of CSVD features

For incident stroke, two studies examined the combined effect of WMHs and lacunes and one of WMHs and CMBs (Table S3). No study examined the combined effect of ≥ three individual features, or examined the association between multiple individual features combined and any of the other outcomes. The combination of two CSVD features was statistically significantly associated with incident ischaemic and haemorrhagic stroke (Fig. 2, Figure S1.1f). This risk was higher than the risk of any individual CSVD feature (Fig. 2).

### 3.7. Sources of heterogeneity

Substantial heterogeneity (I^2^ > 60%) was present in four of the 17 main analyses (Fig. 2). Exploration of heterogeneity showed that more concordance was present in analyses with only high-quality studies and with only studies that measured WMHs on an automated quantitative scale (Tables S6.1-S6.4). Results of these analyses were qualitatively similar to the results of the main analyses.

### 3.8. Potential publication bias

Significant funnel plot asymmetry was found for analyses with stroke (for WMHs on a dichotomous scale, lacunes and CMBs) and all-cause mortality (for WMHs on a dichotomous scale) (Table S7, Figure S3). We used the trim and fill approach to adjust the estimates for funnel plot asymmetry. The adjusted estimates were qualitatively similar to those in the main analyses (Table S8).

### 3.9. Sensitivity analyses

Results were quantitatively similar when analyses were repeated using only high-quality studies; replacing the risk estimates for periventricular WMHs with those for deep WMHs; replacing the risk estimates for deep CMBs with those for lobar CMBs; and replacing adjusted risk estimates with unadjusted risk estimates (Figures S2.1-S2.5). In high-risk populations, the associations between WMHs and lacunes and all-cause dementia were weaker and not statistically significant (Figures S2.1, S2.3). For all studied outcomes, except for depression, the strength of the association was higher for WMHs measured on an automated quantitative scale than for WMHs measured on an observer rated semi-quantitative scale (Figure S2.1). The results of the other post hoc analyses are provided in the supplemental material (Figures S2.1-S2.5).

## 4. Discussion

### 4.1. Main findings

This systematic review and meta-analysis shows that various individual CSVD features are consistently and strongly associated with a higher incidence of ischaemic and haemorrhagic stroke, all-cause dementia and depression, and all-cause mortality. In addition, the combination of two CSVD features is more strongly associated with incident stroke than individual features alone. The combined effect of multiple CSVD features could not be investigated for all-cause dementia or depression, or all-cause mortality. If these associations are causal, the strength of these associations suggests that a substantial burden of disease is attributable to CSVD.

Our findings agree with and extend previous meta-analyses (Charidimou et al., 2017; Charidimou et al., 2013; Charidimou et al., 2018; Debette and Markus, 2010; van Agtmaal et al., 2017; Wang et al., 2015; Wang et al., 2014) on CSVD. These studies evaluated only WMHs, (Debette and Markus, 2010; van Agtmaal et al., 2017; Wang et al., 2014) or only CMBs,(Charidimou et al., 2017; Charidimou et al., 2013; Charidimou et al., 2018; Wang et al., 2015) and found an association with incident stroke, dementia, depression or mortality. The present meta-analysis is the first to evaluate the effect of various CSVD features in different study populations on multiple clinical outcomes.

### 4.2. Methodologic considerations

Some methodological issues warrant consideration. First, the associations with all outcomes were consistent across the different MRI features. Our analyses suggest individual MRI features of CSVD may not carry a differential risk for a specific clinical event, but may reflect disease severity, consistent with the hypothesis that these features are manifestations of the same disease.(Wardlaw et al., 2013) For instance, CMBs were associated not only with incident intracerebral haemorrhage, but also with ischaemic stroke. In accordance, a recent study among individuals with CADASIL (Puy et al., 2017) found that presence of CMBs was associated with ischaemic stroke. Second, there was substantial quantitative heterogeneity in four of the 17 main analyses. More concordance was present among high-quality studies and among studies that measured WMHs on an automated quantitative scale. Third, potential publication bias was present in four of the 17 main analyses. However, additional analyses suggested that this bias may have led to only a slight overestimation of true effect estimates (adjusted estimates calculated using the trim and fill approach were qualitatively similar to the results of the main analysis). Fourth, only two studies evaluated perivascular spaces, and only three studies evaluated the effect of combinations of CSVD features. Evidence for associations between perivascular spaces or combined CSVD features and the outcomes studied is, therefore, weak, and this requires further study. Fifth, effect estimates for stroke were higher than for dementia and depression, and mortality. The assessment of dementia and depression may have a larger measurement error than the assessment of stroke or death. Furthermore, other non-vascular factors may lead to dementia, depression, or greater mortality. Sixth, the association between CMBs and incident dementia was not statistically significant. This needs to be interpreted cautiously, because only four studies with relatively small samples were included in this analysis. Finally, results of most sensitivity analyses were consistent with our main analyses, with few exceptions. For CMBs, risk of haemorrhagic stroke was higher than for ischaemic stroke, which is in accordance with a previous meta-analysis. (Charidimou et al., 2013) This may be due to direct enlargement of CMBs transforming to intracranial haemorrhage.(Akoudad et al., 2015) In addition, in high-risk populations, the associations between WMHs and lacunes and all-cause dementia were weaker than those in the population based studies. Similarly, in stroke populations, the associations between MWHs, lacunes and CMBs and (recurrent) stroke and mortality were weaker. This may be due to the lower quality and smaller sample size of studies done in high-risk populations (only three of these 12 studies had a NOS score > four and their mean sample size was 209) and stroke populations (four of these 21 studies had a NOS score > four and their mean sample size was 861) as compared to population-based studies (11 of these 12 studies had a NOS score > four and their mean sample size was 1484).

### 4.3. Underlying mechanisms

CSVD may lead to stroke, dementia, depression, and mortality through multiple mechanisms. First, CSVD may be a direct cause of stroke, dementia, and depression. Notably, MRI features of CSVD may reflect poor cerebral blood flow regulation, predisposing to ischemia (e.g. due to chronic cerebral hypoperfusion), which may lead to stroke. (Ostergaard et al., 2016) Furthermore, interruption of prefrontal sub-cortical structures or loops involved in cognitive function or mood regulation may directly lead to cognitive decline and depression. (Alexopoulos et al., 1997; Wardlaw et al., 2013) Second, CSVD may indirectly lead to cognitive decline and depression through incident stroke. Similarly, CSVD may increase mortality risk through incident stroke, dementia, and depression. Third, CSVD has been suggested(de la Torre, 2002) to lead to cognitive decline through interaction with Alzheimer’s disease pathology. In accordance, the present study showed that CSVD increased the risk not only of presumed vascular dementia, but also of Alzheimer’s disease. Fourth, CSVD reflecting an individual’s poor health or social economic status might also explain our findings, particularly the association between CSVD and all-cause mortality. Although studies that adjusted for measures of frailty (e.g. walking speed) or education found similar results, we cannot exclude the possibility of residual confounding.

### 4.4. Implications

This review has several clinical and research implications. It shows that individuals with CSVD features are at high risk of ischaemic and haemorrhagic stroke, all-cause dementia, depression and mortality; a risk ratio similar to that of individuals with diabetes(Gregg et al., 2012; Gregg et al., 2014; Xu et al., 2004; Yu et al., 2015) or hypertension. (Barengo et al., 2013; Imano et al., 2009; Li et al., 2007; Sharp et al., 2011; Verdecchia et al., 2005) An incidental finding of CSVD should be recognized as implying a substantial risk of stroke, dementia, depression, and mortality, and should prompt work-up and treatment of relevant risk factors. Prevention of CSVD itself could be an important therapeutic goal, although evidence for effective interventions is lacking. Trials are therefore needed that target suspected mechanisms of CSVD, including blood-brain barrier dysfunction, capillary flow pattern dysregulation, and blood pressure variability.(Ostergaard et al., 2016) In addition, CSVD may be a surrogate marker of risk of stroke, dementia and depression. CSVD features can be quantified reliably(De Guio et al., 2016) and their change over time may be more sensitive than change of cognition.(Benjamin et al., 2016) However, only one previous study(Dufouil et al., 2005) found that reduction of progression of CSVD correlated with reduced occurrence of clinical endpoints, while others(Launer et al., 2011; Williamson et al., 2014) did not. This requires further study. Finally, the present study showed that the combination of two CSVD features is most strongly associated with incident stroke. This suggests that imaging scales that integrate many CSVD features, such as the scale recently developed by Huijts et al.,(Huijts et al., 2013) are most suitable to assess CSVD and most likely to enable improved risk prediction of clinical outcomes beyond established risk factors.

### 4.5. Limitations

This review has some limitations. We only evaluated baseline measurements of CSVD. Baseline measurements may, however, not accurately reflect the exposure to new lesions during follow-up. We also did not evaluate the location of CSVD features, although their clinical consequences may depend upon location, so our estimates are an average over possibly different associations per region. In addition, the pooled estimates for WMHs on a dichotomous scale should be interpreted with caution. Different definitions of this measure were used across studies which hampers its interpretation.

## 5. Conclusion

The present systematic review and meta-analysis shows that various CSVD features are strongly and consistently associated with a higher incidence of ischaemic and haemorrhagic stroke, all-cause dementia and depression, and greater all-cause mortality.

## Supplementary Material

Supplementary Material 1

Supplementary Material 2

Supplementary Material 3

## Appendix A. Supplementary data

Supplementary material related to this article can be found, in the online version, at doi:https://doi.org/10.1016/j.neubiorev.2018.04.003.
