# Supplementary material for: Cerebral small vessel disease and risk of incident stroke, dementia and depression, and all-cause mortality: A systematic review and meta-analysis
Source: Neurosci Biobehav Rev. Author manuscript; Available in PMC 2019 Jul 1. (PMC6123527; doi:10.1016/j.neubiorev.2018.04.003)
Supplement: Supplementary Material 1 [file NIHMS974997-supplement-Supplementary_Material_1.doc]

**MOOSE Statement - Reporting Checklist for Authors, Editors, and Reviewers of Meta-analyses of Observational Studies**

| **Reporting Criteria** | **Reported (Yes/No)** | **Reported on Page** |
| --- | --- | --- |
| **Reporting of Background** |  |  |
| Problem definition | Yes | 4 |
| Hypothesis statement | Yes | 4 |
| Description of Study Outcome(s) | Yes | 6 |
| Type of exposure or intervention used | Yes | 5 |
| Type of study design used | Yes | 5 |
| Study population | Yes | 5 |
| **Reporting of Search Strategy** |  |  |
| Qualifications of searchers (e.g. librarians and investigators) | Yes | 5 |
| Search strategy, including time period  included in the synthesis and keywords | Yes | 5, Supplemental material, Tables S1.1 to S1.4 |
| Effort to include all available studies,  including contact with authors | Yes | 6 |
| Databases and registries searched | Yes | 5 |
| Search software used, name and  version, including special features used  (eg, explosion) |  |  |
| Use of hand searching (eg, reference  lists of obtained articles) | Yes | 5 |
| List of citations located and those  excluded, including justification | Yes | Figure 1, Table S3 |
| Method for addressing articles  published in languages other than  English | Yes | 6 |
| Method of handling abstracts and  unpublished studies | Yes | 6 |
| Description of any contact with authors | Yes | 6 |
| **Reporting of Methods** |  |  |
| Description of relevance or  appropriateness of studies assembled for  assessing the hypothesis to be tested | Yes | 6 |
| Rationale for the selection and coding of  data (e.g. sound clinical principles or  convenience) | Yes | 6 |
| Documentation of how data were  classified and coded (e.g. multiple raters,  blinding, and interrater reliability) | Yes | 6 |
| Assessment of confounding (e.g.  comparability of cases and controls in  studies where appropriate | Yes | 7 |
| Assessment of study quality, including  blinding of quality assessors;  stratification or regression on possible  predictors of study results YES 5 | Yes | 6, Supplemental material, Appendix C |
| Assessment of heterogeneity | Yes | 8, Supplemental material, Table S2 |
| Description of statistical methods (e.g.  complete description of fixed or random  effects models, justification of whether  the chosen models account for predictors  of study results, dose-response models,  or cumulative meta-analysis) in sufficient  detail to be replicated | Yes | 7 |
| Provision of appropriate tables and  graphics | Yes | Figure 1 and 2, Supplemental material |
| **Reporting of Results** |  |  |
| Table giving descriptive information for  each study included | Yes | Supplemental material, Tables S4.1 to S4.4, Tables S5.1 to S5.4 |
| Results of sensitivity testing (e.g.  subgroup analysis) | Yes | Supplemental material, Figures S2.1 to S2.4, Tables S6.1 to S6.4 |
| Indication of statistical uncertainty of  findings | Yes | Figure 2 |
| **Reporting of Discussion** |  |  |
| Quantitative assessment of bias (e.g.  publication bias) | Yes | 12, Supplemental material, Figure S3, Table S7 and S8 |
| Justification for exclusion (e.g. exclusion  of non–English-language citations) | No | N/A |
| Assessment of quality of included studies | Yes | 12, Supplemental material, Tables S5.1 to S5.4 |
| **Reporting of Conclusions** |  |  |
| Consideration of alternative explanations  for observed results | Yes | 13 |
| Generalization of the conclusions (i.e.  appropriate for the data presented and  within the domain of the literature review) | Yes | 18 |
| Guidelines for future research | Yes | 18 |
| Disclosure of funding source | Yes | 19 |
