# Supplementary material for: Cerebral small vessel disease and risk of incident stroke, dementia and depression, and all-cause mortality: A systematic review and meta-analysis
Source: Neurosci Biobehav Rev. Author manuscript; Available in PMC 2019 Jul 1. (PMC6123527; doi:10.1016/j.neubiorev.2018.04.003)
Supplement: Supplementary Material 2 [file NIHMS974997-supplement-Supplementary_Material_2.pdf]

UNIVERSITY of York
Centre for Reviews and Dissemination

NHS
National Institute for Health Research

PROSPERO International prospective register of systematic reviews

Cerebral small vessel disease and risk of stroke, dementia, depression, and all-cause mortality: a systematic review and meta-analysis

Sytze Rensma, Thomas Van Sloten, Lenore Launer, Coen Stehouwer

Citation

Sytze Rensma, Thomas Van Sloten, Lenore Launer, Coen Stehouwer. Cerebral small vessel disease and risk of stroke, dementia, depression, and all-cause mortality: a systematic review and meta-analysis. PROSPERO 2016:CRD42016038521 Available from http://www.crd.york.ac.uk/PROSPERO_REBRANDING/display_record.asp?ID=CRD42016038521

Review question(s)

Are different magnetic resonance imaging (MRI) defined manifestations of cerebral small vessel disease (i.e. higher white matter hyperintensity volume, presence of lacunes, cerebral microbleeds and perivascular spaces, and brain atrophy) associated with a higher risk of incident stroke, dementia, depression and/or all-cause mortality?

Searches

We will identify relevant studies through a search of MEDLINE and EMBASE, from July 1977 (first recorded use of brain MRI) to present, using predefined search terms. In addition, we will identify papers by reviewing the reference list of all relevant articles identified.

There are no language restrictions.

Types of study to be included

Prospective studies will be included that evaluate the association between manifestations of cerebral small vessel disease and incident stroke, dementia, depression and/or all-cause mortality.

Specific inclusion criteria are:

1. MRI defined manifestations of cerebral small vessel disease (as defined by the STandards for Reporting Vascular changes on nEuroimaging [STRIVE] [1]) determined at baseline:

a. Volume of white matter hyperintensities of presumed vascular origin

b. Lacunes of presumed vascular origin, subcortical infarcts and silent brain infarcts. Subcortical infarcts and silent brain infarcts will be included, because most of these infarcts are lacunes of presumed vascular origin [1].

c. Cerebral microbleeds

d. Perivascular spaces

e. Brain atrophy

2. Any of the following outcomes (as defined by the individual studies):

a. Incident clinical stroke (first or recurrent) (not silent stroke)

b. Incident dementia

c. Incident depression (first or recurrent episode)

d. All-cause mortality

3. Study population of at least 50 subjects

4. Mean/median follow-up duration =12 months after brain MRI

5. Studying adults (individuals aged 18 years or older)

6. Full-text article available (no congress abstracts)

Specific exclusion criterion is:

1. Studies only including individuals with cerebral inflammatory or neurodegenerative diseases (e.g. multiple sclerosis, lupus erythematosus, Huntington's disease and Parkinson's disease) or monogenetic cerebrovascular disease (e.g. CADASIL)

Condition or domain being studied

Manifestations of cerebral small vessel disease detected by MRI include white matter hyperintensities, lacunes of presumed vascular origin, cerebral microbleeds, perivascular spaces and cerebral atrophy. Several studies have evaluated the association between these manifestations and various outcomes, including incident stroke, dementia, depression and mortality. However, the results of these studies have not been consistent. Thus far, no study has systematically evaluated the association between different manifestations of cerebral small vessel disease and incident stroke, dementia, depression and/or all-cause mortality.

Participants/ population

All adults in whom the association between manifestations of cerebral small vessel disease and incident stroke, dementia, depression and/or all-cause mortality is evaluated as specified above (please see "types of study to be included"), both in the general population and in a hospital-based setting.

Intervention(s), exposure(s)

The exposure variable is the presence of MRI defined manifestations of cerebral small vessel disease.

Comparator(s)/ control

The control is the group of participants without or with a lower burden of MRI defined manifestations of cerebral small vessel disease.

Outcome(s)

Primary outcomes

1. Incident clinical stroke (first or recurrent)

2. Incident dementia

3. Incident depression (first or recurrent episode)

4. All-cause mortality

Secondary outcomes

1. Specific subtypes of stroke (ischaemic vs. haemorrhagic stroke)

2. Specific subtypes of dementia (vascular dementia vs. Alzheimer's disease)

Data extraction, (selection and coding)

Two independent reviewers (SR and TVS) will select all relevant studies based on title and abstract. Full texts will then be retrieved and assessed for eligibility. In the case of multiple publications from the same cohort, we will include the most up-to-date or comprehensive information. Two independent reviewers (SR, TVS) will extract data with use of a pre-designed data extraction form. Any disagreements between the reviewers will be resolved by consensus. A third independent reviewer is available to solve any persisting disagreements. We will collect information on the following items: study size; follow-up duration; age; sex; diabetes mellitus, prior stroke; other

prior cardiovascular diseases; hypertension; atrial fibrillation; baseline cognitive performance; prior depression; baseline depression scale score; MRI characteristics; definitions of CSVD features; outcome definitions; number of events; statistical analysis used; reported risk estimates; and other variables adjusted for in the analyses.

Risk of bias (quality) assessment

Two reviewers (SR and TVS) will independently assess the risk of bias with the Newcastle-Ottawa Scale. Any disagreements between the reviewers will be resolved by consensus. A third independent reviewer (CS) is available to resolve any persisting disagreements.

Strategy for data synthesis

All analyses will be done with Cochrane Review manager (version 5.3) and R statistical software (version 3.2.3). We will pool results for each manifestation of cerebral small vessel disease when at least three studies are available with the same outcome. For white matter hyperintensity volume and brain atrophy, we will pool results separately for dichotomous and continuous measures. For analysis with combined presence of two or more individual features of CSVD as the determinant, we will pool HRs for any accumulating combination of individual features of CSVD. Continuous measures will be included as per one higher standard deviation (SD). Weighted subgroup SDs will be used when no overall SD is provided. For studies that measure deep and periventricular white matter hyperintensity volumes separately and do not provide a measure of total white matter hyperintensity volume, we will include the results for periventricular white matter hyperintensity volume in the main analysis only, because periventricular white matter hyperintensity volume is more closely related to total white matter hyperintensity volume. For lacunes, cerebral microbleeds and perivascular spaces, we will pool results for dichotomous measures. For studies that measure deep and lobar microbleeds separately and do not provide a total microbleed count, we will include the results for deep microbleeds in the main analysis only, because deep microbleeds are more closely related to the total cerebral microbleed count [1, 4]. Pooled hazard ratios (HRs) will be calculated using the random effects inverse variance method. Most studies will report HRs. Odds ratios and relative risks will be treated as HRs. For each study, we will include the fully adjusted HR (but without adjustments for other manifestations of cerebral small vessel disease). The level of statistical heterogeneity will be evaluated using the I-squared test. High statistical heterogeneity was defined as I-squared >60% . Potential publication bias will be assessed with Egger's test and by construction of funnel plots.

Analysis of subgroups or subsets

The following sensitivity analyses will be done:

1. Analyses will be repeated separately for studies with high risk populations (i.e. individuals with previous stroke or mild cognitive impairment) and population based studies

2. Analyses will be repeated after excluding studies with a relatively high risk of bias (defined as Newcastle-Ottawa scale score <4)

3. Analyses will be repeated including only those studies that defined a lacuna of presumed vascular origin according to STRIVE, i.e. a round or ovoid, subcortical, fluid-filled (cerebrospinal fluid-like) cavity between 3-15 mm on T1 and/or T2 weighted MRI images

4. Analyses will be repeated separately for studies that measured white matter hyperintensity volume on a quantitative scale and those that measured white matter hyperintensity volume on a visual semi-quantitative scale (e.g. Fazekas and Wahlund scale).

5. Analyses will be repeated replacing the results for periventricular white matter hyperintensity volume with those for deep white matter hyperintensity volume

6. Analyses will be repeated replacing the results for deep cerebral microbleeds with those for lobar cerebral microbleeds

7. Analyses will be repeated with unadjusted (or minimally adjusted) risk estimates

8. For white matter hyperintensity volume and brain atrophy, results will be pooled per ten millilitre volume instead

of per SD.

Contact details for further information
Mr Rensma

s.rensma@maastrichtuniversity.nl

Organisational affiliation of the review
Department of Internal Medicine , Maastricht University Medical Centre, Maastricht, the Netherlands

Review team
Mr Sytze Rensma, Maastricht University Medical Centre
Dr Thomas Van Sloten, Maastricht University Medical Centre
Dr Lenore Launer, Laboratory of Epidemiology and Population Sciences, National Institute on Aging, National Institutes of Health
Professor Coen Stehouwer, Maastricht University Medical Centre

Anticipated or actual start date
01 November 2015

Anticipated completion date
01 April 2017

Funding sources/sponsors
Sytze Rensma, Thomas van Sloten and Coen Stehouwer are supported by the European Regional Development Fund as part of Het Operationeel Programma Zuid-Nederland (OP-ZUID), the province of Limburg, Department of Economic Affairs of Netherlands (grant 31O.041), Stichting de Weijerhorst, Pearl String Initiative Diabetes, Cardiovascular Centre Maastricht, Cardiovascular Research Institute Maastricht (CARIM), School for Nutrition, Toxicology and Metabolism (NUTRIM), Stichting Annadal, and Health Foundation Limburg.

Conflicts of interest
None known

Language
English

Country
Netherlands

Subject index terms status
Subject indexing assigned by CRD

Subject index terms
Cerebral Small Vessel Diseases; Dementia; Depression; Depressive Disorder; Humans; Stroke

Any other information
References 1. Wardlaw, J.M., et al., Neuroimaging standards for research into small vessel disease and its contribution to ageing and neurodegeneration. Lancet Neurol, 2013. 12(8): p. 822-38. 2. Wells GA SB, S.B., O'Connell D, Peterson J et al., The Newcastle-Ottawa Scale (NOS) for assessing the quality of nonrandomized studies in meta-analyses. p. http://www.ohri.ca/programs/clinical_epidemiology/oxford.asp. 3. Prasad, K., et al., White matter disease independently predicts progression from mild cognitive impairment to Alzheimer's disease in a clinic cohort. Dement Geriatr Cogn Disord, 2011. 31(6): p. 431-4. 4. Charidimou, A., et al., Cerebral microbleeds and recurrent stroke risk: systematic review and meta-analysis of prospective ischemic stroke and transient ischemic attack cohorts. Stroke, 2013. 44(4): p. 995-1001.

Stage of review
Completed but not published

Date of registration in PROSPERO
04 May 2016

Date of publication of this revision
04 May 2017

**Stage of review at time of this submission**	**Started**	**Completed**
Preliminary searches	Yes	Yes
Piloting of the study selection process	Yes	Yes
Formal screening of search results against eligibility criteria	Yes	Yes
Data extraction	Yes	Yes
Risk of bias (quality) assessment	Yes	Yes
Data analysis	Yes	Yes

PROSPERO
International prospective register of systematic reviews
The information in this record has been provided by the named contact for this review. CRD has accepted this information in good faith and registered the review in PROSPERO. CRD bears no responsibility or liability for the content of this registration record, any associated files or external websites.
