# Supplementary material for: Cerebral small vessel disease and risk of incident stroke, dementia and depression, and all-cause mortality: A systematic review and meta-analysis
Source: Neurosci Biobehav Rev. Author manuscript; Available in PMC 2019 Jul 1. (PMC6123527; doi:10.1016/j.neubiorev.2018.04.003)
Supplement: Supplementary Material 3 [file NIHMS974997-supplement-Supplementary_Material_3.docx]

**Supplemental material**

**Cerebral small vessel disease and risk of incident stroke, dementia and depression, and all-cause mortality: a systematic review and meta-analysis**

Authors:

SP Rensma

TT van Sloten

LJ Launer

CDA Stehouwer

This supplemental file includes the adjusted Newcastle-Ottawa scale, and 8 tables and 3 figures. The MOOSE checklist (Appendix A) and the PROSPERO protocol (Appendix B) are provided as a separate file.

Appendix C. Adjusted Newcastle-Ottawa Scale

Tables S1.1 to S1.4. Search strategy

Table S2. Description of post hoc analyses

Table S3. Number of included studies in the systematic review and pooled analyses

Tables S4.1 to S4.4. Study characteristics

Tables S5.1 to S5.4. Newcastle-Ottawa Scale scores

Tables S6.1 to S6.4. Heterogeneity for main and sensitivity analyses

Table S7. Results of Egger’s tests

Table S8. Results of trim and fill tests

Figures S1.1 to S1.4. Forest plots main analyses

Figures S2.1 to S2.5. Results of sensitivity and post hoc analyses

Figure S3. Funnel plots for analyses with funnel plot asymmetry

**Appendix C. Adjusted Newcastle-Ottawa Scale**For the present study, items 2 and 3 (selection category) of the original Newcastle-Ottawa Scale (NOS) for cohort studies were combined (for the individual items, see below). The original items evaluated the quality of the assessment of the exposed and non-exposed cohorts, respectively. In the present study, however, the total study was “exposed” to the risk factor under study (i.e. cerebral small vessel disease). A study can be awarded a maximum of one star for each numbered item within the selection and outcome categories. A maximum of two stars can be given for comparability.

The individual items of the scale are described below.

Selection

1) Representativeness of the cohort

a) truly representative of the general population *****

b) somewhat representative of the general population *****

c) high risk populations (e.g. individuals with: prior stroke, mild cognitive impairment, cardiovascular disease, prior depression (for the association with incident depression), or individuals receiving dialysis)

d) no description or other cohorts

2) Ascertainment of determinant (cerebral small vessel disease)

a) use of an MRI scanner with a field strength of 1.5 Tesla or higher and the following (minimal) sequences: for white matter hyperintensities: T2-weighted and fluid-attenuated inversion recovery (FLAIR); for lacunes: T1- and(or) T2-weighted; for cerebral microbleeds: T2*-weighted gradient echo sequence; for perivascular spaces: T2-weighted; and for total cerebral atrophy: T1/FLAIR *

b) not a method described above

c) no description

3) Demonstration that outcome of interest was not present at start of study

a) yes *****

b) no

Comparability

1) Comparability of cohorts based on of the design or analysis

a) study controls for cardiovascular risk factors; smoking habits, type 2 diabetes, and systolic blood pressure, and/or use of antihypertensive medication. *****

b) study controls for age, sex, and (for studies evaluating incident dementia) education *****

c) other

Outcome

1) Assessment of outcome

a) objective measurements and/or record linkage and/or expert opinion *****

- For depression: validated criteria, ICD codes
- For dementia: validated cognitive tests, ICD codes
- For stroke: clinical diagnosis
- For mortality: population or hospital register

b) self- and/or peer-report

c) no description/other

2) Was follow-up long enough for outcomes to occur

a) yes (median/mean follow-up duration >4 year) *****

b) no

3) Adequacy of follow-up of cohorts

a) complete follow-up and/or all subjects accounted for *****

b) subjects lost to follow-up unlikely to introduce bias, small number lost (>80 % follow-up), or description provided of those lost *****

c) follow up rate <80% and no description of those lost to follow-up

d) no statement

**Table S1.1 –Search strategy for incident ischaemic and haemorrhagic stroke**

| **PubMed** |
| --- |
| (((“White matter” AND (hyperintens* OR lesion* OR disease* OR change*)) OR (“leukoaraiosis” OR "Leukoaraiosis"[Mesh] OR "Leukoaraiosis/pathology"[Mesh]) OR ((“cerebral small vessel” OR “cerebral small-vessel”) AND disease*) OR "Cerebral Small Vessel Diseases"[Mesh]) OR (((“lacunar” OR “deep” OR “subcortical” OR “silent” OR “small vessel”) AND (infarc* OR Stroke*)) OR "Stroke, Lacunar"[Mesh] OR ((microinfarct* OR microscopic infarct*) AND (“brain” OR cerebral* OR cerebrum*))) OR ((“brain” OR cerebral* OR cerebrum*) AND ((microhemorrhag* OR microbleed* OR microhaemorrhag*) OR (“dot-like” AND (suscept* OR hemosid*)))) OR ((“brain” OR cerebral* OR cerebrum*) AND (Virchow-Robin* OR Virchow Robin* OR “etat crible” OR (perivascular space*))) OR ((“brain” OR cerebral* OR cerebrum*) AND (“Atrophy” OR volum* OR “volume loss”))) AND (“Magnetic Resonance Imaging”[Mesh] OR “MRI” OR “magnetic resonance imaging”) AND ((“Stroke” or ((“brain” OR cerebral* OR cerebrum*) AND (Infarction* OR hemorrhag* OR haemorrhag*)) or "Stroke"[Mesh] or "Stroke/epidemiology"[Mesh] or "Cerebral Hemorrhage"[Mesh] OR "Cerebral Infarction"[Mesh])) AND (“Longitudinal study” OR “Cohort study” OR “Prospective study” OR “Longitudinal Studies”[Mesh] or “Cohort Studies”[Mesh] OR "Prospective Studies"[Mesh]) |
| **Embase** |
| (((exp white matter/ AND (hyperintensity.mp. OR lesion.mp. OR disease.mp. OR exp diseases/)) OR exp white matter lesion/ OR leukoaraiosis.mp. OR cerebral small vessel disease.mp. OR exp cerebrovascular disease/) OR (exp lacunar stroke/ OR ((lacunar.mp. OR deep.mp. OR subcortical.mp. OR silent.mp. OR small vessel.mp.) AND (exp infarction/ OR exp stroke/)) OR ((microinfarct.mp. OR microscopic infarct.mp.) AND (cerebral.mp. OR exp brain/ OR cerebrum.mp.))) OR (((cerebral.mp. OR exp brain/ OR cerebrum.mp.) AND (microhaemorrhage.mp. OR microhemorrhage.mp. OR microbleed.mp.)) OR (dot-like.mp. AND (susceptible.mp. OR hemosiderin.mp.))) OR ((cerebral.mp. OR exp brain/ OR cerebrum.mp.) AND (virchow-robin.mp. OR Virchow robin.mp. OR etat crible.mp. OR perivascular space.mp.)) OR (((cerebral.mp. OR exp brain/ OR cerebrum.mp. OR exp brain size/) AND (exp atrophy/ OR volume loss.mp.)) OR exp brain atrophy/)) AND (((cerebral.mp. OR exp brain/ OR cerebrum.mp.) AND (exp infarction/ OR exp stroke/ OR exp bleeding/ OR microhaemorrhage.mp. OR microhemorrhage.mp.)) OR Exp brain infarction/ OR exp cerebrovascular accident/) AND (MRI.mp. OR magnetic resonance imaging.mp. OR exp nuclear magnetic resonance imaging/) AND (exp longitudinal study/ OR exp cohort analysis/ OR exp prospective study/) |

**Table S1.2 –Search strategy for incident all-cause dementia**

| **PubMed** |
| --- |
| ((((((("White matter" AND (hyperintens* OR lesion* OR disease* OR change*)) OR ("leukoaraiosis" OR "Leukoaraiosis"[Mesh] OR "Leukoaraiosis/pathology"[Mesh]) OR (("cerebral small vessel" OR "cerebral small-vessel") AND disease*) OR "Cerebral Small Vessel Diseases"[Mesh]) OR ((("lacunar" OR "deep" OR "subcortical" OR "silent" OR "small vessel") AND (infarc* OR Stroke*)) OR "Stroke, Lacunar"[Mesh] OR ((microinfarct* OR microscopic infarct*) AND ("brain" OR cerebral* OR cerebrum*))) OR (("brain" OR cerebral* OR cerebrum*) AND ((microhemorrhag* OR microbleed* OR microhaemorrhag*) OR ("dot-like" AND (suscept* OR hemosid*)))) OR (("brain" OR cerebral* OR cerebrum*) AND (Virchow-Robin* OR Virchow Robin* OR "etat crible" OR (perivascular space*))) OR (("brain" OR cerebral* OR cerebrum*) AND ("Atrophy" OR volum* OR "volume loss"))) AND ("Magnetic Resonance Imaging"[Mesh] OR "MRI" OR "magnetic resonance imaging"))) AND (("Dementia" OR "Alzheimer disease" OR "Vascular dementia" OR "Dementia"[Mesh] OR "Dementia/epidemiology"[Mesh] OR "Alzheimer Disease"[Mesh] OR "Dementia, Multi-Infarct"[Mesh] OR "Dementia, Vascular"[Mesh] OR "Mild Cognitive Impairment"[Mesh] OR "cognitive impairment")))) AND (("Longitudinal study" OR "Cohort study" OR "Prospective study" OR "Longitudinal Studies"[Mesh] OR "Cohort Studies"[Mesh] OR "Prospective Studies"[Mesh])) |
| **Embase** |
| (((exp white matter/ AND (hyperintensity.mp. OR lesion.mp. OR disease.mp. OR exp diseases/)) OR exp white matter lesion/ OR leukoaraiosis.mp. OR cerebral small vessel disease.mp. OR exp cerebrovascular disease/) OR (exp lacunar stroke/ OR ((lacunar.mp. OR deep.mp. OR subcortical.mp. OR silent.mp. OR small vessel.mp.) AND (exp infarction/ OR exp stroke/)) OR ((microinfarct.mp. OR microscopic infarct.mp.) AND (cerebral.mp. OR exp brain/ OR cerebrum.mp.))) OR (((cerebral.mp. OR exp brain/ OR cerebrum.mp.) AND (microhaemorrhage.mp. OR microhemorrhage.mp. OR microbleed.mp.)) OR (dot-like.mp. AND (susceptible.mp. OR hemosiderin.mp.))) OR ((cerebral.mp. OR exp brain/ OR cerebrum.mp.) AND (virchow-robin.mp. OR Virchow robin.mp. OR etat crible.mp. OR perivascular space.mp.)) OR (((cerebral.mp. OR exp brain/ OR cerebrum.mp. OR exp brain size/) AND (exp atrophy/ OR volume loss.mp.)) OR exp brain atrophy/)) AND (exp dementia/ OR exp Alzheimer disease/ OR exp multinfarct dementia/ OR vascular dementia.mp. OR exp cognitive defect/ OR cognitive impairment.mp.) AND (MRI.mp. OR magnetic resonance imaging.mp. OR exp nuclear magnetic resonance imaging/) AND (exp longitudinal study/ OR exp cohort analysis/ OR exp prospective study/) |

**Table S1.3 –Search strategy for incident depression**

| **PubMed** |
| --- |
| (((“White matter” AND (hyperintens* OR lesion* OR disease* OR change*)) OR (“leukoaraiosis” OR "Leukoaraiosis"[Mesh] OR "Leukoaraiosis/pathology"[Mesh]) OR ((“cerebral small vessel” OR “cerebral small-vessel”) AND disease*) OR "Cerebral Small Vessel Diseases"[Mesh]) OR (((“lacunar” OR “deep” OR “subcortical” OR “silent” OR “small vessel”) AND (infarc* OR Stroke*)) OR "Stroke, Lacunar"[Mesh] OR ((microinfarct* OR microscopic infarct*) AND (“brain” OR cerebral* OR cerebrum*))) OR ((“brain” OR cerebral* OR cerebrum*) AND ((microhemorrhag* OR microbleed* OR microhaemorrhag*) OR (“dot-like” AND (suscept* OR hemosid*)))) OR ((“brain” OR cerebral* OR cerebrum*) AND (Virchow-Robin* OR Virchow Robin* OR “etat crible” OR (perivascular space*))) OR ((“brain” OR cerebral* OR cerebrum*) AND (“Atrophy” OR volum* OR “volume loss”))) AND (“Magnetic Resonance Imaging”[Mesh] OR “MRI” OR “magnetic resonance imaging”) AND (“Depression” OR “Depression”[Mesh] OR “Depressive Disorder, Major”[Mesh] OR “Suicide” OR "Suicide"[Mesh]) AND (“Longitudinal study” OR “Cohort study” OR “Prospective study” OR “Longitudinal Studies”[Mesh] or “Cohort Studies”[Mesh] OR "Prospective Studies"[Mesh]) |
| **Embase** |
| (((exp white matter/ AND (hyperintensity.mp. OR lesion.mp. OR disease.mp. OR exp diseases/)) OR exp white matter lesion/ OR leukoaraiosis.mp. OR cerebral small vessel disease.mp. OR exp cerebrovascular disease/) OR (exp lacunar stroke/ OR ((lacunar.mp. OR deep.mp. OR subcortical.mp. OR silent.mp. OR small vessel.mp.) AND (exp infarction/ OR exp stroke/)) OR ((microinfarct.mp. OR microscopic infarct.mp.) AND (cerebral.mp. OR exp brain/ OR cerebrum.mp.))) OR (((cerebral.mp. OR exp brain/ OR cerebrum.mp.) AND (microhaemorrhage.mp. OR microhemorrhage.mp. OR microbleed.mp.)) OR (dot-like.mp. AND (susceptible.mp. OR hemosiderin.mp.))) OR ((cerebral.mp. OR exp brain/ OR cerebrum.mp.) AND (virchow-robin.mp. OR Virchow robin.mp. OR etat crible.mp. OR perivascular space.mp.)) OR (((cerebral.mp. OR exp brain/ OR cerebrum.mp. OR exp brain size/) AND (exp atrophy/ OR volume loss.mp.)) OR exp brain atrophy/)) AND (exp depression/ OR exp major depression/ OR exp suicide/ OR exp suicide attempt/) AND (MRI.mp. OR magnetic resonance imaging.mp. OR exp nuclear magnetic resonance imaging/) AND (exp longitudinal study/ OR exp cohort analysis/ OR exp prospective study/) |

**Table S1.4 –Search strategy for all-cause mortality**

| **PubMed** |
| --- |
| (((“White matter” AND (hyperintens* OR lesion* OR disease* OR change*)) OR (“leukoaraiosis” OR "Leukoaraiosis"[Mesh] OR "Leukoaraiosis/pathology"[Mesh]) OR ((“cerebral small vessel” OR “cerebral small-vessel”) AND disease*) OR "Cerebral Small Vessel Diseases"[Mesh]) OR (((“lacunar” OR “deep” OR “subcortical” OR “silent” OR “small vessel”) AND (infarc* OR Stroke*)) OR "Stroke, Lacunar"[Mesh] OR ((microinfarct* OR microscopic infarct*) AND (“brain” OR cerebral* OR cerebrum*))) OR ((“brain” OR cerebral* OR cerebrum*) AND ((microhemorrhag* OR microbleed* OR microhaemorrhag*) OR (“dot-like” AND (suscept* OR hemosid*)))) OR ((“brain” OR cerebral* OR cerebrum*) AND (Virchow-Robin* OR Virchow Robin* OR “etat crible” OR (perivascular space*))) OR ((“brain” OR cerebral* OR cerebrum*) AND (“Atrophy” OR volum* OR “volume loss”))) AND (“Magnetic Resonance Imaging”[Mesh] OR “MRI” OR “magnetic resonance imaging”) AND (“Death” OR “Mortality” OR “Death”[Mesh] OR “all-cause mortality” OR “Mortality”[Mesh] OR “survival analysis”[Mesh] OR “survival analysis”) AND (“Longitudinal study” OR “Cohort study” OR “Prospective study” OR “Longitudinal Studies”[Mesh] or “Cohort Studies”[Mesh] OR "Prospective Studies"[Mesh]) |
| **Embase** |
| (((exp white matter/ AND (hyperintensity.mp. OR lesion.mp. OR disease.mp. OR exp diseases/)) OR exp white matter lesion/ OR leukoaraiosis.mp. OR cerebral small vessel disease.mp. OR exp cerebrovascular disease/) OR (exp lacunar stroke/ OR ((lacunar.mp. OR deep.mp. OR subcortical.mp. OR silent.mp. OR small vessel.mp.) AND (exp infarction/ OR exp stroke/)) OR ((microinfarct.mp. OR microscopic infarct.mp.) AND (cerebral.mp. OR exp brain/ OR cerebrum.mp.))) OR (((cerebral.mp. OR exp brain/ OR cerebrum.mp.) AND (microhaemorrhage.mp. OR microhemorrhage.mp. OR microbleed.mp.)) OR (dot-like.mp. AND (susceptible.mp. OR hemosiderin.mp.))) OR ((cerebral.mp. OR exp brain/ OR cerebrum.mp.) AND (virchow-robin.mp. OR Virchow robin.mp. OR etat crible.mp. OR perivascular space.mp.)) OR (((cerebral.mp. OR exp brain/ OR cerebrum.mp. OR exp brain size/) AND (exp atrophy/ OR volume loss.mp.)) OR exp brain atrophy/)) AND (exp death/ OR exp mortality/ OR exp survival/ OR survival analysis.mp.) AND (MRI.mp. OR magnetic resonance imaging.mp. OR exp nuclear magnetic resonance imaging/) AND (exp longitudinal study/ OR exp cohort analysis/ OR exp prospective study/) |

**Table S2 – Description of post hoc analyses**

| **Post hoc analyses** |
| --- |
| Post hoc analyses were done when at least 3 studies were available per outcome. Results were pooled using only studies with a first episode of ischaemic of haemorrhagic stroke or depression; using only studies with stroke patients; using only hazard ratios (i.e. excluding studies that reported odds ratios or relative risks); using risk estimates comparing highest vs. lowest categories of white matter hyperintensities (irrespective of the number of participants per category), instead of risk estimates comparing higher and lower categories with the highest number of participants and events; using only risk estimates for silent cerebral infarcts; excluding studies that reported risk estimates for silent cerebral infarcts only; using only studies with Newcastle-Ottawa Scale (NOS) score >3; using only studies with NOS score >5; using only risk estimates of periventricular white matter hyperintensities; using only risk estimates of deep white matter hyperintensities; using only risk estimates for deep cerebral microbleeds; and using only risk estimates for lobar cerebral microbleeds**.** |

**Table S3. Number of included studies in the systematic review and pooled analyses**

| Outcome | White matter hyperintensities | | | Lacunes | | Cerebral microbleeds | | Perivascular spaces | | Total cerebral atrophy | | Combination of CSVD features |
| --- | --- | --- | --- | --- | --- | --- | --- | --- | --- | --- | --- | --- |
|  | Systematic review | Pooled analysis* | | Systematic review | Pooled analysis* | Systematic review | Pooled analysis* | Systematic review | Pooled analysis* | Systematic review | Pooled analysis* | Pooled analysis |
|  | Scale | | | | | | | | | | | |
|  | Any | Dichotomous | Continuous | Any | Dichotomous | Any | Dichotomous | Any | Dichotomous | Any | Continuous | Dichotomous |
| Any stroke type | 24 | 23 | 4 | 15 | 15 | 15 | 15 | 0 | 0 | 3 | 3 | 3 |
| Ischaemic stroke |  | 11 | 0 |  | 0 |  | 9 |  | 0 |  | 0 | 0 |
| Haemorrhagic stroke |  | 7 | 0 |  | 0 |  | 8 |  | 0 |  | 0 | 0 |
| All-cause dementia | 24 | 15 | 7 | 11 | 10 | 4 | 4 | 1 | 0 | 8 | 4 | 0 |
| Alzheimer’s disease |  | 8 | 0 |  | 0 |  | 0 |  | 0 |  | 0 | 0 |
| Presumed vascular dementia |  | 4 | 0 |  | 0 |  | 0 |  | 0 |  | 0 | 0 |
| Depression | 9 | 8 | 6 | 2 | 0 | 1 | 0 | 1 | 0 | 3 | 3 | 0 |
| All-cause mortality | 19 | 16 | 6 | 11 | 11 | 10 | 10 | 0 | 0 | 3 | 0 | 0 |

*Studies were excluded from the pooled analysis, because no risk estimates were presented,(Gomar et al., 2011; Inzitari et al., 2009; Prasad et al., 2011; Sluimer et al., 2008; Staff et al., 2010; Steffens et al., 2002b; Steffens et al., 2007; Stoub et al., 2014) less than three studies were available per CSVD feature (Perez, 2013; Staff et al., 2010; van der Veen et al., 2014; van Sloten et al., 2015; Zhu et al., 2010) or white matter hyperintensities were not evaluated on a dichotomous or continuous scale.(Korf et al., 2004; Meguro et al., 2007; Miwa et al., 2014; Prasad et al., 2011; Prins et al., 2013; Tapiola et al., 2008; van Straaten et al., 2008) 30 studies (Bokura et al., 2006; Buyck et al., 2009; Conijn et al., 2011; Debette et al., 2010; DeCarli et al., 2004; Firbank et al., 2012a; Henneman et al., 2009; Ikram et al., 2010a; Ikram et al., 2009; Imaizumi et al., 2015b; Kaffashian et al., 2016a; Kantarci et al., 2009; Kobayashi et al., 1997; Kumral et al., 2015; Miwa et al., 2014; Mok et al., 2009; Naka et al., 2006; Perez, 2013; Poels et al., 2012; Prasad et al., 2011; Putaala et al., 2011; Qiu et al., 2016; Soo et al., 2008; Staekenborg et al., 2009; Stephan et al., 2015; van der Holst et al., 2016; van der Veen et al., 2014; van Sloten et al., 2015; van Uden et al., 2015; Vermeer et al., 2003; Weber et al., 2012; Weinstein et al., 2013; Windham et al., 2015; Yamamoto et al., 2002; Yamauchi et al., 2002) evaluated multiple CSVD manifestations and 22 studies (Andersen et al., 2017; Andersen et al., 2016; Appelros et al., 2005; Benedictus et al., 2015; Bokura et al., 2006; Boulanger et al., 2006; Conijn et al., 2011; Debette et al., 2010; Fan et al., 2003; Firbank et al., 2012a; Fu et al., 2005; Haji et al., 2015; Inzitari et al., 2009; Kaffashian et al., 2016a; Kwa et al., 2012; Mok et al., 2009; Putaala et al., 2011; van der Veen et al., 2014; Weber et al., 2012; Weinstein et al., 2013; Windham et al., 2015; Yamauchi et al., 2002) evaluated multiple outcomes. HRs were reported by 48 studies.(Akoudad et al., 2013; Akoudad et al., 2015; Akoudad et al., 2016; Altmann-Schneider et al., 2011; Andersen et al., 2017; Andersen et al., 2016; Appelros et al., 2005; Benedictus et al., 2015; Bernick et al., 2001; Bokura et al., 2011; Bombois et al., 2008; Boulanger et al., 2006; Buyck et al., 2009; Conijn et al., 2011; Debette et al., 2010; DeCarli et al., 2004; Firbank et al., 2012a; Firbank et al., 2012b; Gerdes et al., 2006; Gioia et al., 2012; Godin et al., 2010; Henneman et al., 2009; Ikram et al., 2009; Ikram et al., 2010b; Ishikawa et al., 2007; Kaffashian et al., 2016a; Kantarci et al., 2009; Kerber et al., 2006; Kim et al., 2015; Kim et al., 2016; Kitagawa et al., 2015; Korf et al., 2004; Kuller, 2003; Kuller et al., 2007; Kuller et al., 2004; Kwa et al., 2012; Lavretsky et al., 2010; Melkas et al., 2012; Miwa et al., 2014; Mok et al., 2009; Naganuma et al., 2015; Naka et al., 2006; Nishikawa et al., 2009; Oksala et al., 2009; Poels et al., 2012; Prins et al., 2013; Prins et al., 2004; Putaala et al., 2011; Sluimer et al., 2008; Smith et al., 2008; Smith et al., 2004; Soo et al., 2008; Staekenborg et al., 2009; Staff et al., 2010; Steffens et al., 2002a; Stephan et al., 2015; Tapiola et al., 2008; Thijs et al., 2010; van der Veen et al., 2014; van Straaten et al., 2008; van Uden et al., 2015; Verdelho et al., 2010; Vermeer et al., 2003; Weinstein et al., 2013; Windham et al., 2015; Yamamoto et al., 2002; Zhu et al., 2010) Fifteen studies (Fan et al., 2003; Fu et al., 2005; Geroldi et al., 2006; Haji et al., 2015; Imaizumi et al., 2015b; Kario et al., 2001; Kobayashi et al., 1997; Kumral et al., 2015; Levy et al., 2003; Lopez et al., 2014; Meguro et al., 2007; Park et al., 2015; Prasad et al., 2011; van Sloten et al., 2015; Weber et al., 2012) reported results as odds ratios or relative risks and these were treated as HRs. Abbreviations: CSVD: cerebral small vessel disease; HRs: hazard ratios.

**Table S4.1 –Characteristics for studies on the association between cerebral small vessel disease and incident ischaemic and haemorrhagic stroke**

Table S4.1.a, Studies on the association between white matter hyperintensities (WMHs) and incident ischaemic and haemorrhagic stroke

| **Reference** | **Study population characteristics** | | | | | | | **MRI characteristics** | **WMHs assessment** | **Stroke type** | **Number of events** | **Outcome** | **Adjustments** |
| --- | --- | --- | --- | --- | --- | --- | --- | --- | --- | --- | --- | --- | --- |
|  | **FU (y)** | **Study** | **N^a^** | **Study participants** | **Country** | **Age (y)** | **Male (%)** |  |  |  |  |  |  |
| Andersen et al., 2017 | 3.3 | NA | 832 | Stroke | Denmark | 59.6 | 58.0 | 1.5-3T, T2, DWI | SQ, (0-6)^‡^, dichotomised (2 vs. 1) for WMHs, (0-3) ^‡^, dichotomised (2 vs. 1) for PVHs and DWMHs | IS | 55 | HR= 1.65 (0.70-3.86) for WMHs grade 2  HR= 2.00 (1.01-3.93) for PVHs grade 2  HR= 1.93 (0.89-4.18) for DWMHs grade 2 | CHA2DS2-VASc score |
| Appelros et al., 2005 | 5.0 | NA | 81 | Stroke | Sweden | 66.4 | 63.0 | T2 | SQ, (0-3)^¥^, dichotomised (2-3 vs. 0-1) | Any type | 24 | HR= 1.70 (1.20-2.70) for WMHs grade 2-3 | DM |
| Bokura et al., 2006 | 6.3 | Shimane | 2,684 | Community-dwelling | Japan | 57.8 | 54.9 | 0.15T, 0.2T, 1.5T, T1, T2, PD, FLAIR | SQ, (0-4 for PVHs, 0-3 for DWMHs)^‡^, dichotomised (3-4 vs. 0-2 for PVHs, 2-3 vs. 0 for DWMHs) | Any type | 102 | OR= 2.08 (1.04-4.17) for PVHs grade 3-4  OR= 2.73 (1.32-5.63) for DWMHs grade 0-2 | Age, sex, BP, HC, DM, smoking, alcohol, family history of stroke |
| Buyck et al., 2009 | 4.9 | 3-CS | 1,232 (1,643) | Community-dwelling | France | 72.3 | 37.4 | 1.5T, T1, T2, PD | Qt, dichotomised (3^th^ quartile vs. lower than median) | Any type | 11 | HR= 2.70 (0.80-9.00) for 3^rd^ quartile WMHs  HR= 3.60 (1.00-12.50) for 3^rd^ quartile PVHs  HR= 2.60 (0.90-7.70) for 3^rd^ quartile DWMHs | Age, sex, BP, HC, DM, smoking, alcohol, WMV |
| Conijn et al., 2011 | 5.3 | SMART-MR | 1,228 | With cardiovascular disease(s) | Netherlands | 58.6 | 79.6 | 1.5T, T1, T2, FLAIR, IR | Qt, continuous (per mL, per SD), dichotomised (5^th^ quintile vs. rest) | IS | 46 | HR= 1.04 (1.01-1.06) per mL WMHs  HR= 1.47 (1.10-1.77) per SD WMHs^2^  HR= 3.60 (1.90-6.90) for 5^th^ quintile WMHs | Age, sex, BMI, BP, HC, DM, smoking, alcohol |
| Debette et al., 2010 | 5.6 | FOS | 2,177 | Community-dwelling | USA | 62 | 47.1 | 1T, 1.5T, T1, T2 | Qt, continuous (log %WMHs, per SD), dichotomised (high vs. low) | Any type  IS | 32  26 | HR= 1.33 (0.93-1.90) per logWMHs  HR= 1.33 (0.93-1.90) per SD WMHs^2^  HR= 2.28 (1.02-5.13) for high WMHs  HR= 2.97 (1.28-6.85) for high WMHs | Age, sex, BP, DM, smoking, alcohol, CerVD |
| Fu et al., 2005 | 1.9 | NA | 228 | Stroke | China | 68.3 | 57.0 | 1.5T, T1, T2, FLAIR, DWI | SQ, (0-3)^¥^, dichotomised (2-3 vs. 0-1) | Any type | 29 | HR= 4.18 (2.04-8.56) per WMHs grade  OR= 4.32 (1.58-11.79) for WMHs grade 2-3 | Age, sex, BP, DM, smoking, alcohol, CVD, AF |
| Gerdes et al., 2006 | 3.5 | NA | 230 | With cardiovascular disease(s) | Netherlands | 62 | 68 | 1.5T, T1, T2, PD | SQ, (0-4 for PVHs, 0-3 for DWMHs)^‡^, dichotomised (2-4 vs. 0 for PVHs, 1-3 vs. 0 for DWMHs) | IS | 21 | HR= 3.60 (1.40-9.20) for PVHs grade 2-4  HR= 1.50 (0.60-3.80) for DWMHs 1-3 | Age, BP, CVD, medication  None |
| Imaizumi et al., 2015b | 4.2 | NA | 226 (305) | Stroke | Japan | NA | 45.1 | 1.5T, T1, T2, PD | SQ, (0-3)^‡^, dichotomised (2-3 vs. 1) | Any type  IS  ICH | 52  40  12 | OR= 2.97 (1.46-6.05) for WMHs grade 2-3  OR= 2.54 (1.10-5.85) for WMHs grade 2-3^2^  OR= 3.22 (0.85-12.2) for WMHs grade 2-3 | None |
| Inzitari et al., 2009 | 2.4 | LADIS | 639 | MCI | Europe | 74.1 | 45.1 | 1.5T, T2*, FLAIR, DWI | SQ, (1-3) ^‡^, dichotomised (3 vs. 1) | Any type | NA | P< 0.05 (p-value for Kaplan-Meier log rank test for difference in WMHs grade for stroke group vs. control) | None |
| Kaffashian et al., 2016a | 9.6 | 3-CS | 1677 | Community-dwelling | France | 72.0 | 39.0 | 1.5T, T1, T2, PD | Qt, continuous (per unit ln(WMHs/TCV, per SD), dichotomised (4^th^ quartile vs. rest) | Any type | 68 | HR= 1.72 (1.24-2.40) per lnWMH  HR= 1.42 (1.15-1.76) per SD^2^  HR= 1.88 (1.16-3.07) for 4^th^ quartile WMHs | Age, sex, ASE, BP, HC, DM, smoking, CVD, APOE |
| Kaffashian et al., 2016b | 9.6 | 3-CS | 1731 | Community-dwelling | France | 72.0 | 39.0 | 1.5T, T1, T2, PD | Qt, continuous (per unit ln(WMHs/TCV), dichotomised (4^th^ quartile vs. rest) | IS  ICH | 54  15 | HR= 1.50 (1.03-2.20) per lnWMH  HR= 1.60 (0.91-2.80) for 4^th^ quartile WMHs  HR= 3.54 (1.65-7.60) per lnWMH  HR= 4.92 (1.43-16.88) for 4^th^ quartile WMHs | Age, sex, ASE, BP, HC, DM, smoking, CVD, APOE |
| Kobayashi et al., 1997 | NA | NA | 933 | Community-dwelling | Japan | NA | 57.4 | 0.15T, 0.2T, T1, T2, PD | Ql, absence vs. presence | Any type | 19 | OR= 4.81 (1.13-20.58) for WMHs presence | None |
| Kuller et al., 2004 | 7.0 | CHS | 1,395 (3,293) | Community-dwelling | USA | 75 | 40.5 | 0.35T-3T, T1, T2, PD | SQ, (0-9)^£^, dichotomised (2 vs. 0-1 for any type stroke, 5-9 vs. 0-1 for IS) | Any type  IS | 117  NA | HR= 1.50 (1.00-2.20) for WMHs grade 2  HR= 2.86 (1.70-4.80) for WMHs grade 5-9 | Age, sex, race, BP, DM, CVD, AF, clinic  None |
| Kumral et al., 2015 | 5.0 | ESR | 9,522 | Stroke | Turkey | 65 | 56.6 | T2, FLAIR | SQ, (0-3) ^‡^, dichotomised (3 vs. 0-2) | Any type  ICH | 2181  NA | OR= 1.53 (1.39-1.69) for WMHs grade 3  OR= 1.88 (1.32-2.66) for WMHs grade 3 | None |
| Melkas et al., 2012 | 5.0 | HSAMC | 320 | Stroke | Finland | 70.8 | 49.7 | 1.0T, T1, T2, PD | SQ, (0-3)^‡^, dichotomised (2-3 vs. 0) | IS | 76 | HR = 1.80 (1.11-2.95) for WMHs grade 2-3 | ASE, BP, AF, peripheral arterial disease |
| Mok et al., 2009 | 5.0 | NA | 75 | Stroke | China | 70.7 | 52.0 | 1.5T, T1, T2, DWI | Qt, continuous (per mL, per SD) | Any type | 12 | HR= 1.97 (1.47-2.62) per mL WMHs  HR= 4.97 (2.49-9.77) per SD WMHs^2^ | Age |
| Naganuma et al., 2013 | 3.4 | ODC | 179 | Receiving haemodialysis | Japan | 58.2 | 63.7 | 1.5T, T1, T2, FLAIR, PD | SQ, (0-3)^‡^, dichotomised (2-3 vs. 0-1 for PVHs and DWMHs) | Any type | 10 | OR= 3.09 (0.82-11.60) for PVHs grade 2-3^2^  OR= 5.96 (1.53-23.24) for DWMHs grade 2-3^2^ | None |
| Naka et al., 2006 | 1.5 | NA | 266 | Stroke | Japan | 67.2 | 62.8 | 1T, T2, T2* | SQ, (0-3)^‡^, dichotomised (2-3 vs. 0-1) | IS ICH | 17  10 | HR= 10.66 (2.60-43.68) for WMHs grade 2-3  HR= 0.02 (0.001-0.26) for WMHs grade 2-3 | Age, sex, vascular risk factors, stroke type, days from stroke onset, CMBs |
| Ntaios et al., 2015 | 2.5 | NA | 617 | Stroke | Greece | 71.0 | 38.2 | T2 | Ql, absence vs. presence | Any type | 351 | HR non-AF group= 2.23 (1.37-3.62) for WMHs presence  HR AF group= 1.83 (0.55-6.17) for WMHs presence  HR total= 2.17 (1.38-3.41) for presence of WMHs2 | CHA2DS2-VASc score |
| Poels et al., 2012 | 10.0 | RSS | 665 (1,007) | Community-dwelling | Netherlands | 72 | 48.2 | 1.5T, T2, 3D HASTE, PD | SQ, (0-9)^1^, dichotomised (3^rd^ tertile vs. 1^st^) | Any type | 77 | HR= 1.80 (0.90-3.70) for 3^rd^ tertile PVHs  HR= 4.80 (2.10-7.80) for 3^rd^ tertile DWMHs | Age, sex, BP, DM, CVD |
| Putaala et al., 2011 | 8.3 | NA | 634 (655) | Stroke | Finland | 40.0 | 58.8 | 1-1.5T, T1, T2, FLAIR | SQ, (0-3)^~^, dichotomised (2-3 vs. 0) | IS | 69 | HR= 1.07 (0.40-2.85) for WMHs grade 2-3 | Age, sex, BP, DM, stroke type, silent cerebral infarcts, prior TIA |
| Smith et al., 2004 | 2.7 | NA | 82 | Stroke | USA | 76.3 | 48.9 | N.A, FLAIR | SQ, (0-9)^1^, dichotomised (2^nd^-3^rd^ tertile vs. 1^st^) | ICH | NA | HR= 9.00 (1.20-67.20) for higher tertiles PVHs | None |
| Soo et al., 2008 | 2.2 | NA | 908 | Stroke | China | 68.4 | 57.7 | 1.5T, T1, T2, T2*, FLAIR | SQ, (0-3)^^^, dichotomised (1-3 vs. 0) | ICH | 96 | HR= 3.47 (0.46-26.25) | None |
| Weinstein et al., 2013 | 7.4 | FOS | 1,414 (1,469) | Community-dwelling | USA | 65.7 | 46.7 | 1T, 1.5T, T1, T2 | Qt, continuous (per SD), dichotomised (5^th^ quintile vs. rest) | Any type | 45 | HR= 1.45 (1.11-1.90) per SD WMHs  HR= 2.73 (1.48-5.02) for 5^th^ quintile WMHs | ASE, BMI, BP, DM, smoking, APOE |
| Weinstein et al., 2013 | 5.9 | FHS | 224 | Community-dwelling | USA | 84.8 | 48.0 | 1T, 1.5T, T1, T2 | Qt, continuous (per SD), dichotomised (5^th^ quintile vs. rest) | Any type | 20 | HR= 0.80 (0.46-1.40) per SD WMHs  HR= 0.60 (0.15-2.37) for 5^th^ quintile WMHs | ASE, BMI, BP, DM, smoking, APOE |
| Windham et al., 2015 | 14.5 | ARIC | 1,881 | Community-dwelling | USA | 62.4 | 40.0 | 1.5T, T1, T2, PD | SQ, (0-9)^ð^, dichotomised (3-9 vs. 0-2) | Any type  IS  ICH | 157  140  15 | HR= 1.30 (1.15-1.46) per WMHs grade  HR= 2.14 (1.45-3.16) for WMHs grade 3-9  HR= 1.30 (1.14-1.47) per WMHs grade  HR= 2.12 (1.41-3.20) for WMHs grade 3-9  HR= 1.42 (0.99-2.03) per WMHs grade  HR= 3.13 (0.93-10.54) for WMHs grade 3-9 | ASE, race, BMI, BP, HC, DM, smoking, alcohol, CVD, centre |
| Yamauchi et al., 2002 | 4.3 | NA | 89 | Stroke or headache | Japan | 66 | 42.7 | 0.5T, T1, T2 | SQ, (0-16)^α^, dichotomised (9-16 vs. 0-8) | Any type | 7 | HR= 1.60 (1.02-2.54) per WMHs grade  OR= 15.30 (2.50-93.90) for WMHs grade 9-16^2^ | Age, sex, BP, DM, smoking, lacunar infarcts, antiplatelet use |

*Tables S4.1.a*. Summary of study characteristics. Abbreviations: APOE: apolipoprotein E; ARIC: Atherosclerosis Risk in Communities Study; ASE: age, sex, education; BP: blood pressure; BMI: body mass index; CerVD: cerebrovascular disease; CHS: Cardiovascular Health Study; CMBs: cerebral microbleeds; CVD: cardiovascular disease; DM: diabetes mellitus; DWMHs: deep white matter hyperintensities; ESR: Ege Stroke Registry; FHS: Framingham Health Study; FLAIR: fluid attenuation inversion recovery; FOS: Framingham Offspring Study; FU: follow-up; HC: hypercholesterolemia; HR: hazard ratio; HSAMC: Helsinki Stroke Aging Memory Cohort; ICH: intracerebral haemorrhage; IR: inverse recovery; IS: ischaemic stroke; LADIS: Leukoaraiosis And DISability; mL: milliliter; MRI: magnetic resonance imaging; NA: not available; ODC: Osaka Dialysis Cohort; OR: odds ratio; PD: proton density; PVHs: periventricular hyperintensities; Ql: qualitative; Qt: quantitative; RSS: Rotterdam Scan Study; SD: standard deviation; SMART-MR: Second Manifestations of ARTerial disease-Magnetic Resonance; SQ: semi-quantitative; TIA: transient ischaemic attack; USA: United States of America; WMHs: white matter hyperintensities; WMV: white matter volume; 3-CS: Three-City Study. ^a^Participant number is presented as amount used in analysis, number between brackets represents total study participant number; ^‡^ Fazekas scale; ^¥^ARWMC scale; ^£^Manolio scale; ^~^Mäntylä scale; ^Wahlund scale; ^ð^ARIC study scale; ^α^Swieten scale; ^1^Rotterdam Scan Study scale; ^2^risk estimate calculated based on information in original article.

Table S4.1.b, Studies on the association between lacunes and incident ischaemic and haemorrhagic stroke

| **Reference** | **Study population characteristics** | | | | | | | **MRI characteristics** | **Lacunes assessment** | **Stroke type** | **Number of events** | **Outcome** | **Adjustments** |
| --- | --- | --- | --- | --- | --- | --- | --- | --- | --- | --- | --- | --- | --- |
|  | **FU (y)** | **Study** | **N^a^** | **Study participants** | **Country** | **Age (y)** | **Male (%)** |  |  |  |  |  |  |
| Andersen et al., 2016 | 2.9 | NA | 786 | Stroke | Denmark | 71.2 | 58.0 | 1T - 3T, T1, T2, T2*, FLAIR | Ql, presence (>2) vs. absence | IS | 46 | HR= 2.52 (1.25-5.09) | Age, sex, BP, DM, CVD |
| Bernick et al., 2001 | 4.2 | CHS | 2,647 (3,324) | Community-dwelling | USA | 59.5 | 57.1 | 1.5-3.0T, T1, T2, FLAIR | Ql, presence (1) vs. 1 | Any type | 124 | HR= 1.44 (0.96-2.16) | Age, sex, BP, DM, AF, CVD, intima media thickness |
| Bokura et al., 2006 | 6.3 | Shimane | 2,684 | Community-dwelling | Japan | 3.7 | 40.4 | 1.5T, T1, T2, PD | Ql, presence vs. absence | Any type | 102 | OR= 3.66 (2.28-5.89) | Age, sex, BP, HC, DM, smoking, alcohol, family history of stroke |
| Conijn et al., 2011 | 5.3 | SMART - MR | 1,228 | With cardiovascular disease(s) | Netherlands | 57.8 | 54.9 | 0.15T, 0.2T, 1.5T, T1, T2, PD, FLAIR | Ql, presence vs. absence | IS | 46 | HR= 3.20 (1.70-5.80) | Age, sex, BMI, BP, HC, DM, smoking, alcohol |
| Debette et al., 2010 | 5.6 | FOS | 2,177 | Community-dwelling | USA | 58.6 | 79.6 | 1.5T, T1, T2, FLAIR, IR | Ql, presence vs. absence | Any type  IS | 32  26 | HR= 2.84 (1.32-6.10)  HR= 3.49 (1.54-7.91) | Age, sex, BP, DM, smoking, alcohol, CerVD |
| Gioia et al., 2012 | 2.1 | NA | 170 | Stroke | Canada | 62 | 47.1 | 1 - 1.5T, T1, T2 | Ql, presence vs. absence | Any type | 19 | HR= 3.20 (1.20-8.70) | Age, sex, BP, HC, DM, smoking, alcohol, drug use, CerVD, migraine, CVD |
| Ishikawa et al., 2007 | 3.4 | NA | 514 | Hypertensive | Japan | 39.4 | 52.4 | 1.5 - 3T, T2, FLAIR | Ql, presence vs. absence | Any type | 43 | HR= 4.60 (1.91-11.03) | Age, BP, smoking, CRP level |
| Kaffashian et al., 2016a | 9.6 | 3-CS | 1677 | Community-dwelling | France | 72.0 | 39.0 | 1.5T, T1, T2, PD | Ql, presence vs. absence | Any type | 68 | HR= 2.69 (1.46-4.95) | Age, sex, ASE, BP, HC, DM, smoking, CVD, APOE |
| Kaffashian et al., 2016a | 9.6 | 3-CS | 1731 | Community-dwelling | France | 72.0 | 39.0 | 1.5T, T1, T2, PD | Ql, presence vs. absence | IS  ICH | 54  15 | HR= 2.12 (1.01-4.42) for 4^th^ quartile WMHs  HR= 8.65 (2.49-30.05) for 4^th^ quartile WMHs | Age, sex, ASE, BP, HC, DM, smoking, CVD, APOE |
| Kario et al., 2001 | 3.5 | NA | 585 (958) | Hypertensive | Japan | 72.0 | 38.0 | 1.5T, T1, T2 | Ql, presence vs. absence | Any type | 45 | HR= 4.63 (2.04-10.5) | Age, sex, BMI, BP |
| Kobayashi et al., 1997 | NA | NA | 933 | Community-dwelling | Japan | 72.3 | 37.2 | 1.5T, T1, T2 | Ql, presence vs. absence | Any type | 19 | OR= 10.48 (3.63-30.21) | None |
| Mok et al., 2009 | 5.0 | NA | 75 | Stroke | China | NA | 57.4 | 0.15 - 0.2T, T1, T2, PD | Ql, presence vs. absence | Any type | 12 | HR= 9.02 (1.16-69.88) | Age |
| Poels et al., 2012 | 10.0 | RSS | 1,007 | Community-dwelling | Netherlands | 70.7 | 52.0 | 1.5T, T1, T2, DWI | Ql, presence vs. absence | Any type | 99 | HR= 2.50 (1.70-3.90) | Age, sex, BP, DM, CVD |
| Putaala et al., 2011 | 8.3 | NA | 609 (655) | Stroke | Finland | 72 | 48.2 | 1.5T, T2, 3D HASTE, PD | Ql, presence (1) vs. absence | IS | 64 | HR= 1.47 (0.68-3.16) | Age, sex, BP, DM, stroke type, silent brain infarcts, prior TIA |
| Weber et al., 2012 | 2.5 | PRoFESS | 815 (1,014) | Stroke | Europe | 66.1 | 63.9 | T1, T2, FLAIR | Ql, absence vs. presence, presence vs. absence | Any type | 90 | OR= 0.58 (0.36-0.94) for absence of lacunes  OR= 1.72 (1.06-2.78) for presence of lacunes^1^ | None |
| Windham et al., 2015 | 14.5 | ARIC | 1,667 (1,799) | Community-dwelling | USA | 40.0 | 58.8 | 1 - 1.5T, T1, T2, FLAIR | Ql, presence vs. absence | Any type  IS  ICH | 157  140  15 | HR= 2.30 (1.49-3.55)  HR= 2.04 (1.28-3.25)  HR= 7.14 (1.63-31.34) | ASE, race, BMI, BP, HC, DM, smoking, alcohol, CVD, centre |

*Table S4.1.b.* Summary of study characteristics. Abbreviations: APOE: apolipoprotein E; ARIC: Atherosclerosis Risk in Communities Study; ASE: age, sex, education; BP: blood pressure; BMI: body mass index; CerVD: cerebrovascular disease; CHS: Cardiovascular Health Study; CVD: cardiovascular disease; DM: diabetes mellitus; FLAIR: fluid attenuation inversion recovery; FOS: Framingham Offspring Study; FU: follow-up; GFR: glomerular filtration rate; HC: hypercholesterolemia; HR: hazard ratio; ICH: intracerebral haemorrhage; IR: inverse recovery; IS: ischaemic stroke; MRI: magnetic resonance imaging; NA: not available; OR: odds ratio; PD: proton density; PRoFESS: Prevention Regimen for Effectively Avoiding Second Strokes trial; Ql: qualitative; RSS: Rotterdam Scan Study; SMART-MR: Second Manifestations of ARTerial disease-Magnetic Resonance; TIA: transient ischaemic attack; USA: United States of America; 3-CS: Three-City Study. ^a^Participant number is presented as amount used in analysis, number between brackets represents total study participant number; ^1^risk estimate calculated based on information in original article.

Table S4.1.c, Studies on the association between cerebral microbleeds (CMBs) and incident ischaemic and haemorrhagic stroke

| **Reference** | **Study population characteristics** | | | | | | | **MRI characteristics** | **CMBs assessment** | **Stroke type** | **Number of events** | **Outcome** | **Adjustments** |
| --- | --- | --- | --- | --- | --- | --- | --- | --- | --- | --- | --- | --- | --- |
|  | **FU (y)** | **Study** | **N^a^** | **Study participants** | **Country** | **Age (y)** | **Male (%)** |  |  |  |  |  |  |
| Akoudad et al., 2015 | 4.9 | RSS | 4,759 | Community-dwelling | Netherlands | 63.8 | 44.7 | 1.5T, T2, 3D HASTE, PD | Ql, presence vs. absence | Any type  IS  ICH | 93  72  11 | HR= 1.79 (1.16-2.78)  HR= 1.40 (0.84-2.34)  HR= 5.41 (1.58-18.46) | Age, sex, BP, HC, DM, smoking, antithrombotic use, clinic |
| Benedictus et al., 2015 | 6.0 | MISTRAL | 301 (333) | AD | Netherlands | 71.2 | 58.0 | 1T - 3T, T1, T2, T2*, FLAIR | Ql, presence vs. absence | Any type | 23 | HR= 3.30 (1.30-8.40) | Age, sex, MMSE, cardiovascular risk factors, WMHs, lacunes |
| Bokura et al., 2011 | 3.6 | Shimane | 2,102 | Community-dwelling | Japan | 62.1 | 53.6 | NA | Ql, presence vs. absence | IS  ICH | 22  10 | HR= 4.48 (2.20-12.20)  HR= 50.20 (16.70-150.90) | Age, sex |
| Boulanger et al., 2006 | 1.2 | Vision | 236 | Stroke | Canada | NA | 55.1 | 3T, T1, T2, T2*, FLAIR | Ql, presence vs. absence | Any type | 24 | HR= 1.50 (0.70-3.60) | Age, WMHs |
| Fan et al., 2003 | 2.3 | NA | 121 | Stroke | China | 68.0 | 67.8 | 1.5T, T1, T2, T2* | Ql, presence vs. absence | Any type | 16 | OR 2.68 (0.92-7.82) | None |
| Haji et al., 2015 | 2.4 | NA | 134 | Stroke | USA | 79.9 | 53.0 | 1.5 - 3T | Ql, presence vs. absence | IS | 56 | OR 1.53 (0.48-4.90)1 | None |
| Imaizumi et al., 2015a | 4.2 | NA | 305 | Stroke | Japan |  |  | 1.5T, T2* FLAIR, DWI | Ql, presence vs. absence (of deep CMBs) | Lacunar infarction | 62 | OR 2.57 (0.93-7.08) | WMHs |
| Kumral et al., 2015 | 5.0 | ESR | 9,522 | Stroke | Turkey | 65.0 | 56.6 | T2, FLAIR | Ql, presence vs. absence | Any type | 2181 | OR= 1.26 (1.08-1.46) | None |
| Kwa et al., 2012 | 3.8 | ESPRIT | 397 | Stroke | Netherlands | 65.3 | 58.4 | 1.0 - 1.5T, T2* | Ql, presence vs. absence | Any type  IS  ICH | 28  23  5 | HR= 2.30 (1.00-5.30)  HR= 2.30 (0.90-5.80)  HR= 2.60 (0.30-27.00) | Age, sex |
| Mok et al., 2009 | 5.0 | NA | 75 | Stroke | China | 70.7 | 52.0 | 1.5T, T1, T2, DWI | Ql, presence vs. absence | Any type | 12 | HR= 5.95 (1.42-24.95) | None |
| Naganuma et al., 2015 | 5.0 | ODC | 179 | Receiving haemodialysis | Japan | 58.2 | 63.7 | 1.5T, T1, T2, T2*, FLAIR, PD | Ql, presence vs. absence | IS  ICH | 12  12 | HR= 1.28 (0.25-6.53)  HR= 21.14 (2.30-194.62) | Age, sex, BMI, BP, HC, DM, smoking, lacunes, PVHs, DWMHs, CVD, AF, dialysis duration, haemoglobin, serum albumin, CRP, anticoagulant use |
| Naka et al., 2006 | 1.5 | NA | 266 | Stroke | Japan | 67.2 | 62.8 | 1T, T2, T2* | Ql, presence vs. absence | IS  ICH | 16  10 | HR= 0.619 (0.17-2.13)  HR= 85.63 (6.34-1155.65) | None |
| Nishikawa et al., 2009 | 3.5 | NA | 698 | Community-dwelling | Japan | 65.4 | 45.7 | 1.5T, T2* | Ql, presence vs. absence | Any type  IS  ICH | 36  10  26 | HR= 2.64 (1.34-5.19)  HR=11.77 (2.95-46.82)  HR= 1.48 (0.63-3.45) | Age, sex, BP |
| Soo et al., 2008 | 2.2 | NA | 908 | Stroke | China | 68.4 | 57.7 | 1.5T, T1, T2, T2*, FLAIR | Ql, presence vs. absence | IS | 96 | HR= 1.35 (0.86-2.11)  HR= 5.99 (1.90-18.86) | None |
| Thijs et al., 2010 | 2.2 | NA | 487 | Stroke | Belgium | 72 .0 | 61.0 | 1 - 3T, T1, T2, FLAIR | Ql, presence vs. absence | Any type | 37 | HR= 2.10 (1.10-4.20) | Age, sex, DM |

*Tables S4.1.c.* Summary of study characteristics. Abbreviations: BP: blood pressure; BMI: body mass index; CMBs: cerebral microbleeds; CVD: cardiovascular disease; DM: diabetes mellitus; DWMHs: deep white matter hyperintensities; ESPRIT: European/Australasian Stroke Prevention in Reversible Ischaemia Trial; ESR: Ege Stroke Registry; FLAIR: fluid attenuation inversion recovery; FU: follow-up; GFR: glomerular filtration rate; HC: hypercholesterolemia; HR: hazard ratio; ICH: intracerebral haemorrhage; IS: ischaemic stroke; MISTRAL: do MIcrobleeds predict STRoke in ALzheimer's disease Study; MRI: magnetic resonance imaging; NA: not available; ODC: Osaka Dialysis Cohort; OR: odds ratio; PD: proton density; PVHs: periventricular hyperintensities; Ql: qualitative; RSS: Rotterdam Scan Study; USA: United States of America; WMHs: white matter hyperintensities. ^a^Participant number is presented as amount used in analysis, number between brackets represents total study participant number; ^1^risk estimate calculated based on information in original article.

Table S4.1.d, Studies on the association between total cerebral atrophy and incident ischaemic and haemorrhagic stroke

| **Reference** | **Study population characteristics** | | | | | | | **MRI characteristics** | **Cerebral atrophy assessment** | **Stroke type** | **Number of events** | **Outcome** | **Adjustments** |
| --- | --- | --- | --- | --- | --- | --- | --- | --- | --- | --- | --- | --- | --- |
|  | **FU (y)** | **Study** | **N^a^** | **Study participants** | **Country** | **Age (y)** | **Male (%)** |  |  |  |  |  |  |
| van der Veen et al., 2014 | 8.3 | SMART - MR | 1,215 | With cardiovascular disease(s) | Netherlands | 58.0 | 80.0 | 1.5T, T1, T2, FLAIR | Qt, continuous, (% ICV, per SD decrease) | IS | 49 | HR= 1.83 (1.33-2.51) per SD decrease in TCV | Age, sex, BMI, BP, DM, smoking, alcohol, intima media thickness |
| Weinstein et al., 2013 | 7.4 | FOS | 1,414 (1,469) | Community-dwelling | USA | 65.7 | 46.0 | 1 - 1.5T, T1, T2 | Qt, continuous, (% ICV, per SD decrease), dichotomised (1^st^ vs. 2^nd^- 5^th^ quintile) | Any type | 45 | HR= 1.51 (1.13-2.01) per SD decrease in TCV  HR= 1.78 (0.92-3.45) for 1^st^ quintile of TCV | ASE, BMI, BP, DM, smoking, APOE |
| Weinstein et al., 2013 | 5.9 | FHS | 224 | Community-dwelling | USA | 84.8 | 48.0 | 1 - 1.5T, T1, T2 | Qt, continuous, (per % ICV, per SD decrease), dichotomised (1^st^ vs. 2^nd^- 5^th^ quintile) | Any type | 20 | HR= 0.86 (0.49-1.53) per SD decrease in cerebral volume  HR= 0.54 (0.13-2.33) for 1^st^ quintile of TCV | ASE, BMI, BP, DM, smoking, APOE |

*Tables S4.1.d* Summary of study characteristics. Abbreviations: APOE: apolipoprotein E; ASE: age, sex, education; BP: blood pressure; BMI: body mass index; DM: diabetes mellitus; FHS: Framingham Health Study; FLAIR: fluid attenuation inversion recovery; FOS: Framingham Offspring Study; FU: follow-up; GFR: glomerular filtration rate; HR: hazard ratio; ICV: intracranial volume; IS: ischaemic stroke; MRI: magnetic resonance imaging; NA: not available; Qt: quantitative; SD: standard deviation; SMART-MR: Second Manifestations of ARTerial disease-Magnetic Resonance; TCV: total cerebral volume; USA: United States of America. ^a^Participant number is presented as amount used in analysis, number between brackets represents total study participant number.

Table S4.1.e, Studies on the association between combinations of CSVD features and incident ischaemic and haemorrhagic stroke

| **Reference** | **Study population characteristics** | | | | | | | **MRI characteristics** | **Definition of combinations of CSVD features** | **Stroke type** | **Number of events** | **Outcome** | **Adjustments** |
| --- | --- | --- | --- | --- | --- | --- | --- | --- | --- | --- | --- | --- | --- |
|  | **FU (y)** | **Study** | **N^a^** | **Study participants** | **Country** | **Age (y)** | **Male (%)** |  |  |  |  |  |  |
| Folsom et al., 2012 | 13.0 | ARIC/CHS | 4,872 | Community-dwelling | USA | 70.8 | 40.2 | 1.5-3.0T, T1, T2, FLAIR | Ql, dichotomised (WMHs grade 3-9 ^ð^ + presence of lacunes vs. WMHs grade 0-2 + absence of lacunes) | ICH | 71 | HR= 4.27 (2.20-8.28) for presence of CSVD | ASE, race, BP, smoking, HC, fibrinogen, intima media thickness, carotid plaque |
| Gioia et al., 2012 | 2.1 | NA | 170 | Stroke | Canada | 62 | 47.1 | 1 - 1.5T, T1, T2 | Ql, dichotomised (presence of WMHs + lacunes vs. absence of WMHs + lacunes) | Any type | 19 | HR= 7.30 (2.30-22.90) for presence of CSVD | Age, sex, BP, HC, DM, smoking, alcohol, drug use, CerVD, migraine, CVD |
| Naka et al., 2006 | 1.5 | NA | 266 | Stroke | Japan | 67.2 | 62.8 | 1T, T2, T2* | Ql, dichotomised (WMHs grade 2-3^‡^ + presence of CMBs presence vs. WMHs grade 0-1 + absence of CMBs | Any type | 24 | OR= 2.80 (0.80-9.00)^4^ for presence of CSVD | None |

*Tables S4.1.e.* Summary of study characteristics. Abbreviations: ARIC: Atherosclerosis Risk in Communities Study; ASE: age, sex, education; BP: blood pressure; BMI: body mass index; CerVD: cerebrovascular disease; CHS: Cardiovascular Health Study; CMBs: cerebral microbleeds; CREDOS: Clinical Research Centre for Dementia of South Korea Study; CVD: cardiovascular disease; DM: diabetes mellitus; FLAIR: fluid attenuation inversion recovery; FU: follow-up; GFR: glomerular filtration rate; HC: hypercholesterolemia; HR: hazard ratio; ICH: intracerebral haemorrhage; MRI: magnetic resonance imaging; NA: not available; NS: non-significant; OR: odds ratio; Ql qualitative; USA: United States of America; WMHs: white matter hyperintensities. ^a^Participant number is presented as amount used in analysis, number between brackets represents total study participant number; ^ð^ARIC study scale.

**Table S4.2 –Characteristics for studies on the association between cerebral small vessel disease and incident all-cause dementia**

Table S4.2.a, Studies on the association between white matter hyperintensities (WMHs) and incident all-cause dementia

| **Reference** | **Study population characteristics** | | | | | | | **MRI characteristics** | **WMHs assessment** | **Dementia type** | **Number of events** | **Outcome** | **Adjustments** |
| --- | --- | --- | --- | --- | --- | --- | --- | --- | --- | --- | --- | --- | --- |
|  | **FU (y)** | **Study** | **N^a^** | **Study participants** | **Country** | **Age (y)** | **Male (%)** |  |  |  |  |  |  |
| Bombois et al., 2008 | 3.8 | NA | 170 | MCI | France | 68.1 | 42.4 | 1.5T, T2, SE, FLAIR | SQ, (0-24^†^, dichotomised (7-24 vs. 0-6) | Any type  VaD  AD | 67  7  29 | HR= 1.01 (0.97-1.05) per unit WMHs  HR= 1.32 (0.77-2.24) for WMHs grade 7-24^3^  HR= 1.14 (1.06-1.24) per unit WMHs  HR= 10.00 (1.55-64.39) for WMHs grade 7-24  HR= 2.71 (1.60-4.58) per unit PVHs  HR= 1.02 (0.96-1.09) per unit WMHs^3^  HR= 1.67 (0.73-3.81) for WMHs grade 7-24^3^ | ASE, cognition AB, BP, HC, DM, MTA |
| Debette et al., 2010 | 5.9 | FOS | 2,013 (2,229) | Community-dwelling | USA | 62.0 | 47.1 | 1-1.5T, T1, T2 | Qt, continuous (per SD), dichotomised (extensive vs. normal) | Any type | 11 | HR= 2.22 (1.32-3.72) Per SD WMHs  HR= 3.97 (1.10-14.30) for extensive WMHs | Age, sex, BP, smoking, DM, CerVD |
| DeCarli et al., 2004 | 3.1 | NA | 52 | MCI | USA | 72.8 | 71.0 | 1.5T, T1, T2 | Qt, continuous (per SD) | Any type | 17 | HR= 0.83 (0.49-1.41) per SD WMHs | ASE, cognition AB, GMV, HCV, lacunes |
| Firbank et al., 2012a | 3.2 | NA | 106 | Stroke | UK | 79.8 | 53.8 | 1.5T, T1, FLAIR | Qt, continuous (per unit ln(WMHs/TCV, per SD), dichotomised (2^nd^-4^th^ quartile vs. first) | Any type | 27 | HR= 1.59 (0.851-2.96) per unit WMHs  HR= 3.05 (1.54-6.03) per SD WMHs^4^  HR= 2.41 (1.11-5.19) for highest quartiles WMHs | MTA, thalamic infarcts, cognition AB |
| Geroldi et al., 2006 | 1.3 | NA | 52 | MCI | Italy | 70.0 | 44.0 | NA | SQ, (0-24)†, dichotomised (7-24 or confluence of lesions vs. 0-6) | Any type | 11 | HR= 2.90 (0.70-11.40)) for WMHs grade 7-24 | None |
| Godin et al., 2010 | 4.0 | 3-CS | 1,139 (1,701) | Community-dwelling | France | 72.3 | 39.3 | 1.5T, T1, T2 | Qt, dichotomised (4^th^ quartile vs. rest) | Any type | 19 | HR= 1.90 (0.70-5.10) for 4^th^ quartile WMHs | ASE, cognition AB, BP, HC, DM, CVD, APOE, ICV |
| Ikram et al., 2010b | 5.9 | RSS | 490 | Community-dwelling | Netherlands | 73.4 | 49.0 | 1.5T, T2, 3D HASTE, PD | Qt, continuous (per SD) | Any type | 46 | HR= 1.57 (1.03-2.38) per SD WMHs | ASE, BP, DM, smoking, BI |
| Inzitari et al., 2009 | 2.4 | LADIS | 639 | MCI | Europe | 74.1 | 45.1 | 1.5T, T1, T2 | SQ, (1-3)^‡^, dichotomised (3 vs. 1) | Any type | NA | P< 0.05 (p-value for Kaplan-Meier log rank test for difference in WMHs grade for stroke group vs. control)^5^ | None |
| Kaffashian et al., 2016a | 7.9 | 3-CS | 1677 | Community-dwelling | France | 72.0 | 39.0 | 1.5T, T1, T2, PD | Qt, continuous (per unit ln(WMHs/TCV), dichotomised (4^th^ quartile vs. rest) | Any type | 124 | HR= 1.72 (1.24-2.40) per lnWMH  HR= 1.73 (1.24-2.59) for 4th quartile WMHs | Age, sex, ASE, BP, HC, DM, smoking, CVD, APOE |
| Kantarci et al., 2009 | 2.1 | NA | 151 | MCI | USA | 77.0 | 58.0 | 1.5T, T1, FLAIR | Qt, dichotomised (1 SD above mean vs. 1 SD below mean) | Any type | 75 | HR= 0.75 (0.42-1.35) for WMHs 1 SD above mean | ASE |
| Kim et al., 2015 | 1.1 | CREDOS | 622 | MCI | Korea | 72.0 | 40.0 | 1.5T, T1, T2, FLAIR | SQ, (1-3)^κ^, dichotomised (3 vs. 1-2) for PVHs and DWMHs | Any type  VaD  AD | 139  25  111 | HR= 2.22 (1.43-3.43) for PVHs grade 3  HR= 0.70 (0.44-1.11) for DWMHs grade 3  HR= 16.14(1.97-132.06) for PVHs grade 3  HR= 1.86 (1.12-3.07) for PVHs grade 3 | ASE, cognition AB, BP, HC, DM, Depression score, Hachinski Ischaemic Score |
| Korf et al., 2004 | 2.8 | NA | 54 | MCI | Sweden | 62.9 | 40.0 | 1.5T, T2, PD | SQ, (0-15)^¥^ | Any type | 37 | HR= 1.01 (0.94-1.08) per WMH grade | None |
| Kuller, 2003 | NA | CHS | 2,939 (3,608) | Community-dwelling | USA | NA | NA | 1.5T, T1, T2 | SQ, (0-9)^£^, dichotomised (0-3 vs 4-9) | Any type  VaD  AD | 480  52  330 | HR= 1.70 (1.36-2.10) for WMHs grade 4-9  HR= 2.10 (1.36-3.11) for WMHs grade 4-9  HR= 1.50 (1.17-1.99) for WMHs grade 4-9 | ASE, cognition AB, BP, DM, CVD, race, APOE, ventricular size, large infarcts |
| Lopez et al., 2014 | 2.0 | Gem Study | 159 (183) | Community-dwelling | USA | 85.5 | 41.5 | 1.5T, T1, T2 | Qt, continuous (per unit WMHs/TCV), dichotomised (4^th^ quartile vs. rest) | Any type | 21 | HR= 3.23 (1.34-7.79) per unit WMHs/TCV  HR= 2.80 (1.25-6.30) for 4^th^ quartile WMHs | ASE, cognition AB, PiB status, HCV |
| Meguro et al., 2007 | 5/7 | NA | 54 | MCI | Japan | NA | NA | 1.5T, T1, T2 | SQ, (1-4) for WMHs and PVHs | VaD  AD | 25  60 | OR= 4.14 (1.54-11.15) per PVHs grade^6^  OR= 0.78 (not significant) per PVHs grade | None |
| Miwa et al., 2014 | 7.5 | OFUS | 524 | With cardiovascular risk factor(s) | Japan | 67.7 | 57.6 | 1.5T, T1, T2, T2* | SQ, (0-30)^#^ | Any type  VaD  AD | 44  18  20 | HR= 1.07 (1.02-1.11) per WMHs grade  HR= 1.14 (1.07-1.21) per WMHs grade  HR= 1.00 (0.93-1.08) per WMHs grade | ASE, APOE |
| Prasad et al., 2011 | 1.5 | NA | 79 | MCI | Singapore | 61.0 | 59.5 | T2, FLAIR | SQ, (0-6)^$^ | Any type | 23 | HR= 2.38 (0.57-10.00) per PVHs grade  HR= 7.69 (1.22-50.00) per DWMHs grade | Age, HC, MTA |
| Prins et al., 2013 | 2.0 | PAGIT | 426 | MCI | International | 71.0 | 45.0 | 1.5T, T1, FLAIR | SQ, (0-15) ^¥^ | Any type | 81 | HR= 0.98 (0.94-1.03) per WMHs grade | Age, sex |
| Prins et al., 2004 | 5.2 | RSS | 810 | Community-dwelling | Netherlands | 72.2 | 48.5 | 1.5T, T1, T2 | SQ, (0-9 for PVHs 0-29.5 for DWMHs)^1^, dichotomised (4-6 vs. 0-3 for PVHs, 2-6 vs. 0-1 for DWMHs) | Any type | 34 | HR= 2.00 (1.00-3.90) for PVHs 3-6  HR= 0.60 (0.27-1.39) for DWMHs 3-6 | Age, sex |
| Smith et al., 2008 | 6.0 | NA | 156 | MCI | USA | 72.3 | 40.0 | 1.5T, T2 | Qt, dichotomised (1 SD above mean vs. 1 SD below mean) | AD | 54 | HR= 1.26 (0.61-2.59) for WMHs 1 SD above mean | None |
| Staekenborg et al., 2009 | 2.0 | NA | 152 | MCI | Netherlands | 69.9 | 53.0 | 1.0T, T1, FLAIR, T2 | SQ, (0-30), dichotomised (6-30 vs. 0-5) for WMHs, PVHs (3-6 vs. 0-2) and DWMHs (4-24 vs. 0-3) | AD  Non-AD | 56  16 | HR= 1.20 (0.70-2.20) for WMHs grade 6-30  HR= 1.10 (0.70-2.00) for PVHs grade 3-6  HR= 1.30 (0.80-2.30) for DWMHs grade 4-24  HR= 5.80 (1.20–26.60) for WMHs 6-30  HR= 6.50 (1.40-29.80) for PVHs 3-6  HR= 5.70 (1.20-26.70) for DWMHs 4-24 | Age, sex |
| Steffens et al., 2007 | 5.4 | NCODE | 161 | Depressive disorder | USA | 69.2 | 37.9 | 1.5T, T2 | Qt, continuous (per mL) | Any type | 20 | (p=0.11) (p-value for T-test for mean difference in WMHs volume for incident dementia group vs. control) | None |
| Stephan et al., 2015 | 7.4 | 3-CS | 1,634 (1,721) | Community-dwelling | France | 72.4 | 39.3 | 1.5T, T1, T2 | Qt, continuous (per ln[WMHs/TCV], per SD) | Any type | 119 | HR= 1.38 (1.05-1.81) per unit WMHs  HR= 1.44 (1.05-1.97) per SD WMHs^4^ | ASE, cognition AB, BP, DM, CVD, digit span, smoking, alcohol, APOE, HCV, ICV, impairment of activity |
| Tapiola et al., 2008 | 2.8 | NA | 60 | MCI | Finland | 72.7 | 31.7 | 1.5T, T2, FLAIR, PD | SQ, (0-15) ^¥^ | Any type | 13 | HR= 1.01 (0.89-1.14) per WMHs grade | None |
| van Straaten et al., 2008 | 3.0 | NA | 152 | MCI | North-America | 72.5 | 54.2 | T1, T2 | SQ, (0-30)# | AD | 55 | HR= 1.01 (0.97-1.05) per WMHs grade | Age, education, MTA, treatment arm |
| van Uden et al., 2015 | 5.2 | RUNDMC | 500 (503) | Community-dwelling | Netherlands | 65.6 | 56.8 | 1.5T, T1, T2, T2* | Qt, continuous (per log mL, per SD) | Any type | 42 | HR= 1.83 (0.80-4.21) per unit WMHs  HR= 1.74 (0.80-3.77) per SD WMHs^4^ | ASE, cognition AB, territorial infarcts, GMV, HCV |
| Verdelho et al., 2010 | 3.0 | LADIS | 442 | MCI | Europe | 74.1 | 45.0 | 0.5T of 1.5T T2 | SQ, (0-4) ^‡^, dichotomised (2 vs. 1) | VaD  AD | 54  22 | HR= 0.29 (0.06-1.41) for WMHs grade 2  HR= 1.69 (0.71-4.02) for WMHs grade 2 | Age, PB, DM, MTA, cerVD |
| Weinstein et al., 2013 | 5.9 | FHS | 224 | Community-dwelling | USA | 84.8 | 48.0 | 1.5T, T1, T2 | Qt, continuous (per SD), dichotomised (5^th^ quintile vs. rest) | AD | 28 | HR= 0.97 (0.65-1.45) per SD WMHs  HR= 1.13 (0.43-2.95) for 5^th^ quintile WMHs | ASE, BP, DM, smoking, BMI, APOE |
| Yamamoto et al., 2002 | 8.9 | NA | 177 | Stroke | Japan | 69.1 | 62.7 | 1.5T, T2 | SQ, (0-3)^2^, dichotomised (2 vs. 0-1) | Any type | 26 | HR= 7.13 (1.63-31.5) for DWMHs grade 2 | Age, sex, cognition AB, Lacunar grade, dipper vs. Non-dipper |

*Tables S4.2.a*. Summary of study characteristics. Abbreviations: AB: at baseline; AD: Alzheimer’s disease; APOE: apolipoprotein E; ASE: age, sex, education; BP: blood pressure; BI: brain infarcts; BMI: body mass index; CerVD: cerebrovascular disease; CHS: Cardiovascular Health Study; CREDOS: Clinical Research Centre for Dementia of South Korea Study; CVD: cardiovascular disease; DM: diabetes mellitus; DWMHs: deep white matter hyperintensities; FHS: Framingham Health Study; FLAIR: fluid attenuation inversion recovery; FOS: Framingham Offspring Study; FU: follow-up; GMV: grey matter volume; HC: hypercholesterolemia; HCV: hippocampal volume; HR: hazard ratio; ICV: intracranial volume; LADIS: Leukoaraiosis And DISability; mL: milliliter; MRI: magnetic resonance imaging; MTA: medial temporal lobe atrophy; NA: not available; NCODE: Neurocognitive Outcomes of Depression in the Elderly study; OFUS: Osaka Follow-Up Study; OR: odds ratio; PAGIT: Placebo-Arm of Galantamine-International-11 Trial; PD: proton density; PiB: Pittsburgh compound B; PVHs: periventricular hyperintensities; Qt: quantitative; RSS: Rotterdam Scan Study; SD: standard deviation; SQ: semi-quantitative; RUNDMC: Radboud University Nijmegen Diffusion Tensor and Magnetic Resonance Cohort; TCV: total cerebral volume; UK: United Kingdom; USA: United States of America; VaD: vascular dementia; WMHs: white matter hyperintensities; 3-CS: Three-City Study. ^a^Participant number is presented as amount used in analysis, number between brackets represents total study participant number; ^†^Scheltens scale; ^‡^ Fazekas scale; ^¥^ARWMC scale; ^£^Manolio scale; ^#^modified Scheltens scale; ^$^combination of Scheltens and adjusted Fazekas scale; ^κ^CREDOS scale; ^1^Rotterdam Scan Study scale; ^2^Schmidt scale; ^3^data published by Debette and Markus, 2010; ^4^risk estimate calculated based on information in original article; ^5^P-value calculated based on information in original article; ^6^unadjusted, confidence intervals calculated based on information in original article.

Table S4.2.b, Studies on the association between lacunes and incident all-cause dementia

| **Reference** | **Study population characteristics** | | | | | | | **MRI characteristics** | **Lacunes assessment** | **Dementia type** | **Number of events** | **Outcome** | **Adjustments** |
| --- | --- | --- | --- | --- | --- | --- | --- | --- | --- | --- | --- | --- | --- |
|  | **FU (y)** | **Study** | **N^a^** | **Study participants** | **Country** | **Age (y)** | **Male (%)** |  |  |  |  |  |  |
| Debette et al., 2010 | 5.9 | FOS | 2,013 (2,229) | Community-dwelling | USA | 62.0 | 47.1 | 1-1.5T, T1, T2 | Ql, presence vs. absence | Any type | 11 | HR= 6.12 (1.82-20.54) | Age, sex, BP, DM, smoking, CVD |
| DeCarli et al., 2004 | 3.1 | NA | 52 | MCI | USA | 72.8 | 71.0 | 1.5T, T1, T2 | Ql, presence vs. absence | Any type | 17 | HR= 2.88 (0.51-15.54) | ASE, cognition AB, GMV, HCV, WMHs |
| Kaffashian et al., 2016a | 7.9 | 3-CS | 1677 | Community-dwelling | France | 72.0 | 39.0 | 1.5T, T1, T2, PD | Ql, presence vs. absence | Any type | 124 | HR= 2.69 (1.46-4.95) | Age, sex, ASE, BP, HC, DM, smoking, CVD, APOE |
| Kantarci et al., 2009 | 2.1 | NA | 151 | MCI | USA | 77.0 | 58.0 | 1.5T, T1, FLAIR | Ql, presence vs. absence | Any type | 75 | HR= 0.82 (0.40-1.90) | ASE |
| Kitagawa et al., 2015 | 8.0 | OFUS | 600 | With cardiovascular risk factor(s) | Japan | 67.4 | 57.0 | 1.5, T1, T2, FLAIR | Ql, presence vs. absence | Any type  VaD  AD | 57  18  31 | HR= 2.64 (1.22-6.09)  HR= 1.78 (0.64-5.44)  HR= 5.37 (1.20-39.2) | ASE, cognition AB, BP, DM, CVD, APOE, GFR |
| Prasad et al., 2011 | 1.5 | NA | 79 | MCI | Singapore | 61.0 | 59.5 | T2, FLAIR | Ql, presence vs. absence | Any type | 23 | (p = 0.309) (P-value of T-test for mean difference in number of lacunes between incident dementia and control) | None |
| Prins et al., 2013 | 2.0 | PAGIT | 426 | MCI | International | 71.0 | 45.0 | 1.5T, T1, FLAIR | Ql, presence vs. absence | Any type | 81 | HR= 1.19 (0.75-1.88) | Age, sex |
| Rosano et al., 2007 | 4.3 | CHS | 155 | Community-dwelling | USA | 77.4 | 40.0 | 1.5T, T1, T2 | Ql, presence vs. absence | AD | 40 | HR= 3.50 (1.10-10.90) | ASE, cognition AB, race, APOE, ICV |
| Staekenborg et al., 2009 | 2.0 | NA | 152 | MCI | Netherlands | 69.9 | 53.0 | 1.0T, T1, FLAIR, T2 | Ql, presence vs. absence | AD  Non-AD | 56  16 | HR= 1.10 (0.50-2.20)  HR= 2.10 (0.70-6.40) | Age, sex |
| van Uden et al., 2015 | 5.2 | RUNDMC | 500 (503) | Community-dwelling | Netherlands | 65.6 | 56.8 | 1.5T, T1, T2, T2* | Ql, presence vs. absence | Any type | 42 | HR= 0.88 (0.44-1.76) | ASE, cognition AB, territorial infarct |
| Vermeer et al., 2003 | 3.6 | RSS | 1,015 | Community-dwelling | Netherlands | 72.1 | 48.0 | 1.5T, T1, T2 | Ql, presence vs. absence | Any type | 30 | HR= 2.26 (1.09-4.70) | ASE |
| Yamamoto et al., 2002 | 8.9 | NA | 177 | Stroke | Japan | 69.1 | 62.7 | 1.5T, T2 | Ql, 3-5 vs. 0-2 | Any type | 26 | HR= 2.38 (0.898-6.47) | Age, sex, PB, HC, DM, DWMHs, dipper vs. Non-dipper |

*Tables S4.2.b*. Summary of study characteristics. Abbreviations: AB: at baseline; AD: Alzheimer’s disease; APOE: apolipoprotein E; ASE: age, sex, education; BP: blood pressure; CHS: Cardiovascular Health Study; CVD: cardiovascular disease; DM: diabetes mellitus; DWMHs: deep white matter hyperintensities; FLAIR: fluid attenuation inversion recovery; FOS: Framingham Offspring Study; FU: follow-up; GFR: glomerular filtration rate; GMV: grey matter volume; HC: hypercholesterolemia; HCV: hippocampal volume; HR: hazard ratio; ICV: intracranial volume; MRI: magnetic resonance imaging; NA: not available; OFUS: Osaka Follow-Up Study; PAGIT: Placebo-Arm of Galantamine-International-11 Trial; PD: proton density; Ql: qualitative; RSS: Rotterdam Scan Study; RUNDMC: Radboud University Nijmegen Diffusion Tensor and Magnetic Resonance Cohort; USA: United States of America; VaD: vascular dementia; WMHs: white matter hyperintensities; 3-CS: Three-City Study. ^a^Participant number is presented as amount used in analysis, number between brackets represents total study participant number.

Table S4.2.c, Studies on the association between cerebral microbleeds (CMBs) and incident all-cause dementia

| **Reference** | **Study population characteristics** | | | | | | | **MRI characteristics** | **CMBs assessment** | **Dementia type** | **Number of events** | **Outcome** | **Adjustments** |
| --- | --- | --- | --- | --- | --- | --- | --- | --- | --- | --- | --- | --- | --- |
|  | **FU (y)** | **Study** | **N^a^** | **Study participants** | **Country** | **Age (y)** | **Male (%)** |  |  |  |  |  |  |
| Akoudad et al., 2016 | 4.8 | RSS | 4,841 | Community-dwelling | Netherlands | 63.8 | 45.0 | 1.5T, T1, T2 | Ql, presence vs. absence | Any type  AD | 72  53 | HR= 1.59 (0.88-2.89)  HR= 1.67 (0.83-3.36) | ASE, BP, HC, DM, APOE, smoking, antithrombotic use |
| Miwa et al., 2014 | 7.5 | OFUS | 524 | With cardiovascular risk factor(s) | Japan | 67.7 | 57.6 | 1.5T, T1, T2, T2* | Ql, presence vs. absence | Any type  VaD  AD | 44  18  20 | HR= 1.71 (0.87–3.27)  HR= 1.22 (0.32–3.72)  HR= 3.36 (1.25–8.88) | Age, sex, cognition AB, CVD, MTA, WMHs |
| Staekenborg et al., 2009 | 2.0 | NA | 152 | MCI | Netherlands | 69.9 | 53.0 | 1.0T, T1, FLAIR, T2 | Ql, presence vs. absence | AD  Non-AD | 56  16 | HR= 0.80 (0.20-2.20)  HR= 2.60 (0.90-7.50) | Age, sex |
| van Uden et al., 2015 | 5.2 | RUNDMC | 500 (503) | Community-dwelling | Netherlands | 65.6 | 56.8 | 1.5T, T1, T2, T2* | Ql, presence vs. absence | Any type | 42 | HR= 0.60 (0.25-1.43) | ASE, cognition AB, territorial infarct |

*Tables S4.2.c*. Summary of study characteristics. Abbreviations: AB: at baseline; AD: Alzheimer’s disease; APOE: apolipoprotein E; ASE: age, sex, education; BP: blood pressure; CMBs: cerebral microbleeds; CVD: cardiovascular disease; DM: diabetes mellitus; FLAIR: fluid attenuation inversion recovery; FU: follow-up; HC: hypercholesterolemia; HR: hazard ratio; MRI: magnetic resonance imaging; MTA: medial temporal lobe atrophy; NA: not available; OFUS: Osaka Follow-Up Study; Ql: qualitative; RSS: Rotterdam Scan Study; RUNDMC: Radboud University Nijmegen Diffusion Tensor and Magnetic Resonance Cohort; VaD: vascular dementia; WMHs: white matter hyperintensities. ^a^Participant number is presented as amount used in analysis, number between brackets represents total study participant number.

Table S4.2.d, Studies on the association between perivascular spaces and incident all-cause dementia

| **Reference** | **Study population characteristics** | | | | | | | **MRI characteristics** | **Perivascular spaces assessment** | **Dementia type** | **Number of events** | **Outcome** | **Adjustments** |
| --- | --- | --- | --- | --- | --- | --- | --- | --- | --- | --- | --- | --- | --- |
|  | **FU (y)** | **Study** | **N^a^** | **Study participants** | **Country** | **Age (y)** | **Male (%)** |  |  |  |  |  |  |
| Zhu et al., 2010 | 4.0 | 3-CS | 505 (1,178) | Community-dwelling | France | 72.5 | 36.4 | 1.5T, T1, T2 | SQ, (1-4), dichotomised (grade 2 vs. 1) | Any type | 7 | HR= 3.1 (0.7-13.9) | Age, APOE, ICV |

*Tables S4.2.d*. Summary of study characteristics. Abbreviations: APOE: apolipoprotein E; FU: follow-up; HR: hazard ratio; ICV: intracranial volume; MRI: magnetic resonance imaging; SQ: semi-quantitative; 3-CS: Three-City Study. ^a^Participant number is presented as amount used in analysis, number between brackets represents total study participant number.

Table S4.2.e, Studies on the association between total cerebral atrophy and incident all-cause dementia

| **Reference** | **Study population characteristics** | | | | | | | **MRI characteristics** | **Cerebral atrophy assessment** | **Dementia type** | **Number of events** | **Outcome** | **Adjustments** |
| --- | --- | --- | --- | --- | --- | --- | --- | --- | --- | --- | --- | --- | --- |
|  | **FU (y)** | **Study** | **N^a^** | **Study participants** | **Country** | **Age (y)** | **Male (%)** |  |  |  |  |  |  |
| Firbank et al., 2012a | 3.2 | NA | 106 | Stroke | UK | 79.8 | 53.8 | 1.5T, T1, FLAIR | Qt, continuous (per mL, per SD) | Any type | 28 | HR= 1.00 (0.99-1.00) per mL increase TCV  HR= 1.51 (0.92-2.49) per SD decrease in TCV^1^ | None |
| Gomar et al., 2011 | 2.0 | ADNI | 320 | MCI | USA | 74.9 | 63.4 | 1.5T, NA | Qt, continuous (per mm^3^) | Any type | 60 | p=0.04 (p-value of F-test for mean difference in baseline TCV between incident dementia and control) | None |
| Henneman et al., 2009 | 3.0 | NA | 72 | MCI | Netherlands | 69.0 | 52.6 | 1.0T, T1 | Qt, dichotomised (higher than median vs. lower than median) | AD | 26 | HR= 1.40 (0.60-3.60) for TCV lower than median | Age, sex, cognition AB |
| Steffens et al., 2002b | 1.9 | MHCRC | 97 | Depressed | USA | 70.2 | 29.6 | 1.5T, T2 | Qt, continuous (per mL) | Dementia | 14 | HR= 1.002 (1.00-1.01) per mL increase in TCV | Age, cognition AB, left hippocampal volume |
| Stephan et al., 2015 | 6.7 | 3-CS | 1,634 (1,721) | Community-dwelling | France | 72.4 | 39.3 | 1.5T, T1, T2 | Qt, continuous (per TCV/ICV, per SD) | Any type | 119 | HR= 0.94 (0.88-1.01) per unit higher TCV  HR= 1.46 (0.94-2.18) per SD decrease in TCV^1^ | ASE, cognition AB, BP, DM, CVD, digit span, smoking, alcohol, APOE, HCV, ICV, impairment of activity |
| Stoub et al., 2014 | 8.5 | ROS | 65 | MCI | USA | 74.2 | 32.3 | 1.5T, T1 | Qt, continuous (mm^3^) | AD | 15 | p<0.05 (p-value for t-test for mean difference in baseline TCV between incident dementia and control) | None |
| Weinstein et al., 2013 | 5.9 | FOS | 1,288 | Community-dwelling | USA | 65.7 | 46.7 | 1.5T, T1, T2 | Qt, continuous (per SD), dichotomised (1^st^ quintile vs. rest) | AD | 63 | HR= 1.28 (0.89-1.83) per SD decrease in TCV  HR= 1.47 (0.54-4.04) for lowest 5^th^ quintile TCV | ASE, BP, DM, smoking, BMI, APOE |
| Weinstein et al., 2013 | 5.9 | FHS | 224 | Community-dwelling | USA | 84.8 | 48.0 | 1.5T, T1, T2 | Qt, continuous (per SD), dichotomised (1^st^  quintile vs. rest) | AD | 28 | HR= 2.41 (1.51-3.84) per SD decrease in TCV  HR= 6.69 (2.62-17.09) for lowest 1^st^ quintile TCV | ASE, BP, DM, smoking, BMI, APOE |

*Tables S4.2.e*. Summary of study characteristics. Abbreviations: AB: at baseline; AD: Alzheimer’s disease; ADNI: Alzheimer's Disease Neuroimaging Initiative; APOE: apolipoprotein E; ASE: age, sex, education; BP: blood pressure; BMI: body mass index; CVD: cardiovascular disease; DM: diabetes mellitus; FHS: Framingham Health Study; FLAIR: fluid attenuation inversion recovery; FOS: Framingham Offspring Study; FU: follow-up; HCV: hippocampal volume; HR: hazard ratio; ICV: intracranial volume; MHCRC: Mental Health Clinical Research Centre Study; mL: milliliter; MRI: magnetic resonance imaging; NA: not available; Qt: quantitative; ROS: Religious Order Study; SD: standard deviation; TCV: total cerebral volume; UK: United Kingdom; MV: white matter volume; 3-CS: Three-City Study. ^a^Participant number is presented as amount used in analysis, number between brackets represents total study participant number; ^1^risk estimate calculated based on information in original article.

**Table S4.3 –Characteristics for studies on the association between cerebral small vessel disease and incident depression**

Table S4.3.a, Studies on the association between white matter hyperintensities (WMHs) and incident depression

| **Reference** | **Study population characteristics** | | | | | | | **MRI characteristics** | **WMHs assessment** | **Depression type** | **Number of events** | **Outcome** | **Adjustments** |
| --- | --- | --- | --- | --- | --- | --- | --- | --- | --- | --- | --- | --- | --- |
|  | **FU (y)** | **Study** | **N^a^** | **Study participants** | **Country** | **Age (y)** | **Male (%)** |  |  |  |  |  |  |
| Godin et al., 2008 | 4.0 | 3-CS | 956 | Community-dwelling | France | NA | NA | 1.5T, T1, T2, PD | Qt, continuous (per quartile, per SD), dichotomised (2^nd^ quartile vs. 1^st^ quartile) | Any type | 103 | OR= 2.03 (1.29-4.19) per SD^1^  OR= 1.20 (0.60-2.30) for 2^nd^ quartile WMHs  OR= 1.30 (1.10-1.70) per quartile | Age, sex, BP, CVD, smoking, alcohol, baseline depression score, impairment of activity, WMV |
| Ikram et al., 2010a | 7.5 | RSS | 443 | Community-dwelling | Netherlands | 73.4 | 50.0 | 1.5T, T1, T2 | Qt, continuous (per SD) | Any type | 35 | HR= 0.83 (0.59-1.18) per SD WMHs | ASE |
| Kim et al., 2016 | 1.2 | CREDOS | 287 (590) | MCI | Korea | 73.0 | 46.3% | 1.5T, T1, T2, FLAIR, GE | SQ, (1-3)^κ^, dichotomised (PVHs 3 vs. 1-2, and DWMHs 2-3 vs 1) | Any type | 45 | HR= 1.25 (0.63-2.48) for PVHs grade 3  HR= 2.75 (1.43-5.28) for DWMHs grade 2-3 | None |
| Park et al., 2015 | 3.0 | NA | 54 | High risk of depression | Korea | NA | NA | 3T, FLAIR | Qt, continuous (logmL, per SD)  SQ, (0-4)^‡^, dichotomised (2-4 vs. 0-1) | Any type | 4 | OR= 5.26 (1.01-26.68) per SD WMHs  OR= 8.14 (1.37-48.22) for WMHs grade 2-4 | ASE, MMSE, baseline depression score  None |
| Perez, 2013 | 3.6 | RSS | 961 (1,047) | Community-dwelling | Netherlands | NA | NA | 1.5T, T1, T2 | SQ, (0-9)^‡^, dichotomised (PVHs 5-9 vs. 0-4, and DWMHs $\geq2mL$ vs. <2mL) | Any type | 60 | OR= 1.30 (0.60-2.60) for PVHs score 5-9  OR= 2.10 (1.10-3.90) for DWMHs ($\geq2mL$) | ASE, MMSE |
| Qiu et al., 2016 | 6.6 | FOS | 1212 | Community-dwelling | USA | 60.0 | 47.6 | 1T, T2 | Qt, continuous (logmL, per SD), dichotomised (1 SD above mean vs. rest) | Any type | 110 | OR= 1.17 (0.93-1.48) per logmL WMHs  OR= 1.16 (0.93-1.45) per SD WMHs^1^  OR= 1.60 (0.89-2.88) for WMHs 1 SD above mean | ASE, time to MRI, living alone, and CES-D at seventh examination |
| Steffens et al., 2002a | 7.0 | CHS | 3,236 | Community-dwelling | USA | NA | 40.4 | 0.35T-1.5T, T2, PD | SQ, (0-9)^β^, dichotomised (6-9 vs. 0-5) | Any type | 1033 | OR= 1.21 (0.73-2.00) for WMHs score 6-9 | Age, sex, race, BP, CVD, 3MSE, antidepressants, APOE |
| Teodorczuk et al., 2007 | 1.0 | LADIS | 526 | With WMHs | Europe | 73.9 | 44.7 | 0.5-1.5T, FLAIR | Qt, continuous (logmL, per SD) | Any type | 85 | OR= 1.63 (1.20-2.20) per logmL WMHs  OR= 1.60 (1.19-2.12) per SD WMHs^1^ | Education, MMSE, history of depression, QoL, worsening IADL, incident stroke |
| Teodorczuk et al., 2010 | 3.0 | LADIS | 399 | With WMHs | Europe | 73.6 | 45.6 | 0.5-1.5T, FLAIR | Qt, continuous (logmL, per SD) | Any type | 82 | OR= 1.36 (1.04-1.76) per logmL WMHs  OR= 1.34 (1.04-1.70) per SD WMHs^1^ | Education, MMSE, history of depression, QoL, worsening IADL, incident stroke |
| van Sloten et al., 2015 | 5.2 | AGES-R | 1,949 | Community-dwelling | Iceland | 74.6 | 43.4 | 1.5T, T1, T2, T2*, FLAIR | Qt, continuous (per SD), dichotomised (4^th^ quartile vs. rest) | Any type | 197 | OR= 1.02 (0.88-1.19) per SD WMHs  OR= 1.12 (0.78-1.60) for 4^th^ quartile WMHs^2^ | ASE, BMI, BP, DM, cognition/depression /geriatric/anxiety scores, smoking, alcohol, coronary calcium score, head coil, FU time |
| Versluis et al., 2006 | 2.8 | PROSPER | 484 | With cardiovascular disease(s) | Netherlands | 74.9 | 57.0 | 1.5T, FLAIR | Qt, dichotomised (2^nd^- 4^th^ quartile vs. 1^st^) | Any type | 31 | OR= 1.20 (0.40-3.50) for highest quartiles WMHs | Age, sex, pravastatin use |

*Tables S4.3.a* Summary of study characteristics. Abbreviations: APOE: apolipoprotein E; AGES-R: Age, Gene/Environment Susceptibility-Reykjavik Study; ASE: age, sex, education; BP: blood pressure; BMI: body mass index; CHS: Cardiovascular Health Study; CREDOS: Clinical Research Centre for Dementia of South Korea Study; CVD: cardiovascular disease; DM: diabetes mellitus; DWMHs: deep white matter hyperintensities; FLAIR: fluid attenuation inversion recovery; FOS: Framingham Offspring Study; FU: follow-up; HR: hazard ratio; LADIS: Leukoaraiosis And DISability; mL: milliliter; MRI: magnetic resonance imaging; NA: not available; OR: odds ratio; PD: proton density; PROSPER: PROspective Study of Pravastatin in the Elderly at Risk; PVHs: periventricular hyperintensities; Qt: quantitative; RSS: Rotterdam Scan Study; SD: standard deviation; SQ: semi-quantitative; USA: United States of America; WMHs: white matter hyperintensities; WMV: white matter volume; 3-CS: Three-City Study. ^a^Participant number is presented as amount used in analysis, number between brackets represents total study participant number; ^‡^Fazekas scale; ^β^CHS scale; ^κ^CREDOS scale; ^1^risk estimate calculated based on information in original article; ^2^unpublished data.

Table S4.3.b, Studies on the association between lacunes and incident depression

| **Reference** | **Study population characteristics** | | | | | | | **MRI characteristics** | **Lacunes assessment** | **Depression type** | **Number of events** | **Outcome** | **Adjustments** |
| --- | --- | --- | --- | --- | --- | --- | --- | --- | --- | --- | --- | --- | --- |
|  | **FU (y)** | **Study** | **N^a^** | **Study population** | **Country** | **Age (y)** | **Male (%)** |  |  |  |  |  |  |
| Perez, 2013 | 3.6 | RSS | 961 (1,047) | Community-dwelling | Netherlands | NA | NA | 1.5T, T1, T2 | Ql, presence vs. absence | Any type | 60 | OR= 1.00 (0.50-1.80) | ASE, MMSE |
| van Sloten et al., 2015 | 5.2 | AGES-R | 1,949 | Community-dwelling | Iceland | 74.6 | 43.4 | 1.5T, T1, T2, T2*, FLAIR | Ql, presence vs. absence | Any type | 197 | OR= 1.83 (1.10-3.05) | ASE, BMI, BP, DM, cognition/depression /geriatric/anxiety scores, smoking, alcohol, coronary calcium score, head coil, FU time |

*Tables S4.3.b.* Summary of study characteristics. Abbreviations; AGES-R: Age, Gene/Environment Susceptibility-Reykjavik Study; ASE: age, sex, education; BP: blood pressure; BMI: body mass index; DM: diabetes mellitus; FLAIR: fluid attenuation inversion recovery; FU: follow-up; HC: hypercholesterolemia; MRI: magnetic resonance imaging; NA: not available; OR: odds ratio; Ql: qualitative; RSS: Rotterdam Scan Study. ^a^Participant number is presented as amount used in analysis, number between brackets represents total study participant number.

Table S4.3.c, Studies on the association between cerebral microbleeds (CMBs) and incident depression

| **Reference** | **Study population characteristics** | | | | | | | **MRI characteristics** | **CMBs assessment** | **Depression type** | **Number of events** | **Outcome** | **Adjustments** |
| --- | --- | --- | --- | --- | --- | --- | --- | --- | --- | --- | --- | --- | --- |
|  | **FU (y)** | **Study** | **N^a^** | **Study population** | **Country** | **Age (y)** | **Male (%)** |  |  |  |  |  |  |
| van Sloten et al., 2015 | 5.2 | AGES-R | 1,949 | Community-dwelling | Iceland | 74.6 | 43.4 | 1.5T, T1, T2, T2*, FLAIR | Ql, presence vs. absence | Any type | 197 | OR= 1.10 (0.73-1.66) | ASE, BMI, BP, DM, cognition/depression /geriatric/anxiety scores, smoking, alcohol, coronary calcium score, head coil, FU time |

*Tables S4.3.c.* Summary of study characteristics. Abbreviations: AGES-R: Age, Gene/Environment Susceptibility-Reykjavik Study; ASE: age, sex, education; BP: blood pressure; BMI: body mass index; CMBs: cerebral microbleeds; DM: diabetes mellitus; FLAIR: fluid attenuation inversion recovery; FU: follow-up; MRI: magnetic resonance imaging; OR: odds ratio; Ql: qualitative. ^a^Participant number is presented as amount used in analysis, number between brackets represents total study participant number.

Table S4.3.d, Studies on the association of perivascular spaces and incident depression

| **Reference** | **Study population characteristics** | | | | | | | **MRI characteristics** | **Perivascular spaces assessment** | **Depression type** | **Number of events** | **Outcome** | **Adjustments** |
| --- | --- | --- | --- | --- | --- | --- | --- | --- | --- | --- | --- | --- | --- |
|  | **FU (y)** | **Study** | **N^a^** | **Study population** | **Country** | **Age (y)** | **Male (%)** |  |  |  |  |  |  |
| van Sloten et al., 2015 | 5.2 | AGES-R | 1,949 | Community-dwelling | Iceland | 74.6 | 43.4 | 1.5T, T1, T2, T2*, FLAIR | Ql, presence vs. absence | Any type | 197 | OR= 1.08 (0.70-1.66) | ASE, BMI, BP, DM, cognition/depression/geriatric/anxiety scores, smoking, alcohol, coronary calcium score, head coil, FU time |

*Tables S4.3.d.* Summary of study characteristics. Abbreviations: AGES-R: Age, Gene/Environment Susceptibility-Reykjavik Study; ASE: age, sex, education; BP: blood pressure; BMI: body mass index; DM: diabetes mellitus; FLAIR: fluid attenuation inversion recovery; FU: follow-up; MRI: magnetic resonance imaging; OR: odds ratio; Ql: qualitative. ^a^Participant number is presented as amount used in analysis, number between brackets represents total study participant number.

Table S4.3.e, Studies on the association between total cerebral atrophy and incident depression

| **Reference** | **Study population characteristics** | | | | | | | **MRI characteristics** | **Cerebral atrophy assessment** | **Depression type** | **Number of events** | **Outcome** | **Adjustments** |
| --- | --- | --- | --- | --- | --- | --- | --- | --- | --- | --- | --- | --- | --- |
|  | **FU (y)** | **Study** | **N^a^** | **Study population** | **Country** | **Age (y)** | **Male (%)** |  |  |  |  |  |  |
| Ikram et al., 2010a | 7.5 | RSS | 443 (479) | Community-dwelling | Netherlands | 73.4 | 50.0 | 1.5T, T1, T2 | Qt, continuous (per SD) | Any type | 35 | HR= 0.83 (0.59-1.18) per SD increase in TCV  HR= 1.12 (0.70-1.82) per SD decrease in TCV^1^ | ASE |
| Qiu et al., 2016 | 6.6 | FOS | 1,212 | Community-dwelling | USA | 60.0 | 47.6 | 1T, T2 | Qt, continuous (logmL, per SD), | Any type | 110 | OR= 0.80 (0.67-0.95) per logmL increase in TCV  OR= 2.02 (1.18-3.53) per SD decrease in TCV^1^ | ASE, time to MRI, living alone, and CES-D at seventh examination |
| van Sloten et al., 2015 | 5.2 | AGES-R | 1,949 | Community-dwelling | Iceland | 74.6 | 43.4 | 1.5T, T1, T2, T2*, FLAIR | Qt, continuous (per SD) | Any type | 197 | OR= 1.23 (1.04-1.45) per SD decrease in TCV | ASE, BMI, BP, DM, cognition/depression /geriatric/anxiety scores, smoking, alcohol, coronary calcium score, head coil, FU time |

*Tables S4.3.e.* Summary of study characteristics. Abbreviations: AGES-R: Age, Gene/Environment Susceptibility-Reykjavik Study; ASE: age, sex, education; BP: blood pressure; BMI: body mass index; DM: diabetes mellitus; FLAIR: fluid attenuation inversion recovery; FOS: Framingham Offspring Study; FU: follow-up; HR: hazard ratio; mL: milliliter; MRI: magnetic resonance imaging; OR: odds ratio; Qt: quantitative; RSS: Rotterdam Scan Study; SD: standard deviation; TCV: total cerebral volume; USA: United States of America. ^a^Participant number is presented as amount used in analysis, number between brackets represents total study participant number; ^1^risk estimate calculated based on information in original article.

**Table S4.4 –Characteristics for studies on the association between cerebral small vessel disease and all-cause mortality**

Table S4.4.a, Studies on the association between white matter hyperintensities (WMHs) and all-cause mortality

| **Reference** | **Study population characteristics** | | | | | | | **MRI characteristics** | **WMHs assessment** | **Number of events** | **Outcome** | **Adjustments** |
| --- | --- | --- | --- | --- | --- | --- | --- | --- | --- | --- | --- | --- |
|  | **FU (y)** | **Study** | **N^a^** | **Study participants** | **Country** | **Age (y)** | **Male (%)** |  |  |  |  |  |
| Andersen et al., 2017 | 3.3 | NA | 832 | Stroke | Denmark | 59.6 | 58.0 | 1.5-3T, T2, DWI | SQ, (0-6)^‡^, dichotomised (2 vs. 1) for WMHs, (0-3) ^‡^, dichotomised (2 vs. 1) for PVHs and DWMHs | 80 | HR= 1.45 (0.66-3.17) for WMHs grade 2  HR= 1.79 (0.98-3.25) for PVHs grade 2  HR= 1.59 (0.80-3.16) for DWMHs grade 2 | CHA2DS2-VASc score |
| Appelros et al., 2005 | 5.0 | NA | 81 | Stroke | Sweden | 66.4 | 63.0 | T2 | SQ, (0-3) ^¥^, dichotomised (high vs. low) | 15 | HR= 1.60 (1.20-2.20) for high WMHs | None |
| Bokura et al., 2006 | 6.3 | Shimane | 2,684 | Community-dwelling | Japan | 57.8 | 54.9 | 0.15- 1.5T, T1, T2 | SQ, (0-4 for PVHs, 0-3 for DWMHs)^‡^, dichotomised (3-4 vs. 0-2 for PVHs, 2-3 vs. 0 for DWMHs) | 93 | OR= 4.01 (1.91-8.45) for PVHs grade 3-4  OR= 1.03 (0.45-2.53) for DWMHs grade 2-3 | Age, sex, HC, BP, DM, smoking, alcohol, family history of stroke |
| Conijn et al., 2011 | 5.3 | SMART-MR | 1,228 | With cardiovascular disease(s) | Netherlands | 58.6 | 79.6 | 1.5T, T1, T2, FLAIR | Qt, continuous (per mL, per SD), dichotomised (5^th^ quintile vs. rest) | 106 | HR= 1.03 (1.01-1.05) per mL WMHs  HR= 1.33 (1.10-1.61) per SD WMHs  HR= 2.00 (1.30-3.00) for 5^th^ quintile WMHs | Age, sex, BMI, BP, HC, DM, smoking, alcohol |
| Debette et al., 2010 | 5.6 | FOS | 2,208 (2,229) | Community-dwelling | USA | 62 | 47.1 | 1-1.5T, T1, T2 | Qt, continuous (per SD), dichotomised (high vs. low) | 97 | HR= 1.38 (1.13-1.69) per SD WMHs  HR= 2.27 (1.41-3.65) for high WMHs | Age, sex, BP, DM, smoking, alcohol, CerVD |
| Firbank et al., 2012a | 3.2 | NA | 106 | Stroke | UK | 79.8 | 53.8 | 1.5T, T1, FLAIR | Qt, continuous (lnWMHs%, per SD) | 60 | HR= 1.18 (0.87-1.60) per lnWMHs  HR= 1.23 (0.84-1.81) per SD WMHs | None |
| Fu et al., 2005 | 1.9 | NA | 228 | Stroke | China | 68.3 | 57.0 | 1.5T, T1, T2, FLAIR, DWI | SQ, (0-3)^¥^, dichotomised (2-3 vs. 0-1) | 25 | OR= 2.02 (1.03-3.96) per WMHs grade  OR= 3.60 (1.30-9.98) for WMHs grade 2-3 | Age, sex, BP, DM, smoking, alcohol, CVD, AF |
| Henneman et al., 2009 | 2.6 | NA | 1,117 (1,138) | MCI | Netherlands | 66 | 55.2 | 1-1.5T, T1, T2, T2*, FLAIR | SQ, (0-3)^†^, dichotomised (3 vs. 0) | 153 | HR= 1.20 (1.00-1.40) per WMHs  HR= 1.70 (1.00-2.80) for WMHs grade 3 | Age, sex, HC, BP, DM, CVD |
| Ikram et al., 2009 | 8.4 | RSS | 490 | Community-dwelling | Netherlands | 73.4 | 49.2 | 1.5T, T1, T2*, FLAIR, PD | Qt, continuous (per SD), dichotomised (4^th^ quartile vs 1^st^) | 191 | HR= 1.38 (1.16-1.65) per SD WMHs  HR= 2.05 (1.32-3.20) for 4^th^ quartile WMHs | Age, sex |
| Inzitari et al., 2009 | 2.4 | LADIS | 639 | MCI | Europe | 74.1 | 45.10 | 1.5T, T1, T2 | SQ, (0-4)^‡^ | NA | P<0.001 (p-value for Kaplan-Meier log rank test for difference in WMHs grade for mortality group vs. control) | None |
| Kerber et al., 2006 | 11.8 | NA | 72 (108) | With imbalance, nested cohort study | USA | 81.9 | 41.7 | 1.5T, T1, T2 | SQ, (0-2)^γ^, dichotomised (2 vs. 0) | 40 | HR= 2.31 (1.21-4.40) for WMHs grade 2 | HC, BP, DM, CVD |
| Kuller et al., 2007 | 10.0 | CHS | 3245 | Community-dwelling | USA | 74.8 | 40.0 | 0.35-3T, T1, T2, PD | SQ, (0-9)^β^, dichotomised (2 vs. 0-1) | 72 | HR= 1.46 (1.23-1.72) for WMHs grade 2 | ASE, BP, DM, Cognition AB, smoking, CVD, APOE, renal insufficiency, walking pace, FEV1 and subclinical disease |
| Kuller et al., 2007 | 5.5 | NA | 259 | Depressed | USA | 70.0 | 29.3 | 1.5T, T1, T2, PD | SQ, (0-3)^θ^, dichotomised (2-3 vs. 0-1) | 30 | OR= 2.36 (1.07-5.21) for PVHs grade 2-3^1^  HR= 3.43 (1.29-9.08) for DWMHs grade 2-3 | None  Age, sex, race, CIRS score |
| Mok et al., 2009 | 5.0 | NA | 75 | Stroke | China | 70.7 | 52 | 1.5T, T1, T2, DWI | Qt, continuous (per mL, per SD) | 16 | HR= 1.78 (1.35-2.35) per mL WMHs  HR= 3.91 (2.03-7.55) per SD WMHs | Age |
| Oksala et al., 2009 | 12.0 | HSAMC | 396 | Stroke | Finland | 70.8 | 48.5 | 1.0T, T1, T2, PD | SQ, (0-3)^¥^, dichotomised (3 vs. 0) | 277 | HR= 1.34 (1.03-1.73) for WMHs grade 3 | Age, sex, HC, BP, CVD, AF, smoking, disability score |
| Putaala et al., 2011 | 8.3 | NA | 630 (655) | Stroke | Finland | 40.0 | 58.8 | 1-1.5T, T1, T2, FLAIR | SQ, (0-3)^γ^, dichotomised (2-3 vs. 0) | 53 | HR= 3.43 (1.58-7.42) for WMHs grade 2-3 | Age, sex, BP, DM, stroke type, silent brain infarcts, history of TIA |
| van der Holst et al., 2016 | 7.8 | RUNDMC | 494 (503) | Community-dwelling | Netherlands | 65.7 | 56.5 | 1.5T, T1, T2*, FLAIR, DTI | Qt, continuous (per SD), dichotomised (4^th^ quartile vs. rest) | 80 | HR= 1.62 (1.24-2.11) per SD WMHs  OR= 1.81 (1.17-2.80) for 4th quartile WMHs^1^ | Age, sex, BP, DM, smoking  None |
| Windham et al., 2015 | 14.5 | ARIC | 1,667 (1,799) | Community-dwelling | USA | 62.4 | 40 | 1.5T, T1, T2, PD | SQ, (0-9)^δ^, dichotomised (3-9 vs. 0-2) | 576 | HR= 1.20 (1.12-1.29) per WMHs grade  OR= 1.78 (1.42-2.23) for WMHs grade 3-9 | ASE, race, BMI, BP, HC, DM, smoking, alcohol, CVD, centre |
| Yamauchi et al., 2002 | 4.3 | NA | 89 | Stroke | Japan | 66 | 42.7 | 0.5T, T1, T2 | SQ, (0-16)^α^, dichotomised (1-16 vs. 0) | 4 | OR= 0.28 (0.03-2.90) for WMHs grade 1-16^1^ | None |

*Tables S4.4.a.* Summary of study characteristics. Abbreviations: AB: at baseline; APOE: apolipoprotein E; ARIC: Atherosclerosis Risk in Communities Study; ASE: age, sex, education; BP: blood pressure; BMI: body mass index; CerVD: cerebrovascular disease; CHS: Cardiovascular Health Study; CIRS: Cumulative Illness Rating Scale; CVD: cardiovascular disease; DM: diabetes mellitus; DWMHs: deep white matter hyperintensities; FLAIR: fluid attenuation inversion recovery; FOS: Framingham Offspring Study; FU: follow-up; HC: hypercholesterolemia; HR: hazard ratio; HSAMC: Helsinki Stroke Aging Memory Cohort; LADIS: Leukoaraiosis And DISability; mL: milliliter; MRI: magnetic resonance imaging; NA: not available; OR: odds ratio; PD: proton density; PVHs: periventricular hyperintensities; Qt: quantitative; RSS: Rotterdam Scan Study; SD: standard deviation; SQ: semi-quantitative; RUNDMC: Radboud University Nijmegen Diffusion Tensor and Magnetic Resonance Cohort; SMART-MR: Second Manifestations of ARTerial disease-Magnetic Resonance; TIA: transient ischaemic attack; UK: United Kingdom; USA: United States of America; WMHs: white matter hyperintensities. ^a^Participant number is presented as amount used in analysis, number between brackets represents total study participant number; ^†^Scheltens scale; ^‡^ Fazekas scale; ^¥^ARWMC scale; ^ð^ARIC study scale; ^α^Swieten scale; ^β^CHS scale; ^γ^scale not specified; ^θ^Coffey scale; ^1^risk estimate calculated based on information in original article.

Table S4.4.b, Studies on the association between lacunes and all-cause mortality

| **Reference** | **Study population characteristics** | | | | | | | **MRI characteristics** | **Lacunes assessment** | **Number of events** | **Outcome** | **Adjustments** |
| --- | --- | --- | --- | --- | --- | --- | --- | --- | --- | --- | --- | --- |
|  | **FU (y)** | **Study** | **N^a^** | **Study population** | **Country** | **Age (y)** | **Male (%)** |  |  |  |  |  |
| Andersen et al., 2016 | 2.9 | NA | 786 | Stroke | Denmark | 59.5 | 57.1 | 1.5-3.0T, T1, T2, FLAIR | Ql, Presence ($\geq$2) vs. absence (0) | 69 | HR= 0.65 (0.33-1.50) | Age, sex, BP, DM, CVD |
| Bokura et al., 2006 | 6.3 | Shimane | 2,684 | Community-dwelling | Japan | 57.8 | 54.9 | 0.15-1.5T, T1, T2, | Ql, Presence vs. absence | 93 | OR= 1.95 (1.16-3.29) | Age, sex, HC, BP, DM, smoking, alcohol, family history of stroke |
| Conijn et al., 2011 | 5.3 | SMART-MR | 1,228 | With cardiovascular disease(s) | Netherlands | 58.6 | 79.6 | 1.5T, T1, T2, FLAIR | Ql, Presence vs. absence | 106 | HR= 2.60 (1.70-3.90) | Age, sex, BMI, BP, HC, DM, smoking, alcohol |
| Debette et al., 2010 | 5.6 | FOS | 2,208 (2,229) | Community-dwelling | USA | 62 .0 | 47.1 | 1-1.5T, T1, T2 | Ql, Presence vs. absence | 97 | HR= 1.53 (0.94-2.48) | Age, sex, BP, DM, smoking, alcohol, CerVD |
| Ikram et al., 2009 | 8.4 | RSS | 490 | Community-dwelling | Netherlands | 73.4 | 49.2 | 1.5T, T1, T2*, FLAIR, PD | Ql, Presence vs. absence | 191 | HR= 1.25 (0.90-1.73) | Age, sex |
| Lavretsky et al., 2010 | 4.7 | IVDPP | 498 | Dementia | USA | 74.5 | 50.2 | 1.5T, T1, T2, PD | Ql, Presence vs. absence | 175 | HR= 1.90 (1.40-2.50) | None |
| Mok et al., 2009 | 5.0 | NA | 75 | Stroke | China | 70.7 | 52.0 | 1.5T, T1, T2, DWI | Ql, Presence vs. absence | 16 | HR= 2.21 (0.70-6.94) | Age |
| Putaala et al., 2011 | 8.3 | NA | 607 (651) | Stroke | Finland | 40.0 | 58.8 | 1-1.5T, T1, T2, FLAIR | Ql, Presence (1) vs. absence | 50 | HR= 1.32 (0.51-3.38) | Age, sex, BP, DM, stroke type, WMHs, history of TIA |
| van der Holst et al., 2016 | 7.8 | RUNDMC | 494 (503) | Community-dwelling | Netherlands | 65.7 | 56.5 | 1.5T, T1, T2*, FLAIR, DTI | Ql, Presence vs. absence | 78 | OR= 1.85 (1.21-2.83)^1^ | None |
| Weber et al., 2012 | 2.5 | PRoFESS | 815 (1,014) | Stroke | Europe | 66.1 | 63.9 | T1, T2, FLAIR | Ql, Absence vs. presence, Presence vs. absence | 20 | OR= 0.58 (0.36-0.94) for absence of lacunes  OR= 2.22 (1.12-4.35) for presence of lacunes^1^ | None |
| Windham et al., 2015 | 14.5 | ARIC | 1,167 (1,799) | Community-dwelling | USA | 62.4 | 40.0 | 1.5T, T1, T2, PD | Ql, Presence vs. absence | 576 | HR= 1.69 (1.31-2.17) | ASE, race, BMI, BP, HC, DM, smoking, alcohol, CVD, centre |

*Tables S4.4.b*. Summary of study characteristics. Abbreviations: ARIC: Atherosclerosis Risk in Communities Study; ASE: age, sex, education; BP: blood pressure; BMI: body mass index; CerVD: cerebrovascular disease; CVD: cardiovascular disease; DM: diabetes mellitus; FLAIR: fluid attenuation inversion recovery; FOS: Framingham Offspring Study; FU: follow-up; HC: hypercholesterolemia; HR: hazard ratio; IVDPP: Ischaemic Vascular Dementia Program Project; MRI: magnetic resonance imaging; NA: not available; OR: odds ratio; PD: proton density; PRoFESS: Prevention Regimen for Effectively Avoiding Second Strokes trial; Ql: qualitative; RSS: Rotterdam Scan Study; RUNDMC: Radboud University Nijmegen Diffusion Tensor and Magnetic Resonance Cohort; SMART-MR: Second Manifestations of ARTerial disease-Magnetic Resonance; TIA: transient ischaemic attack; USA: United States of America; WMHs: white matter hyperintensities. ^a^Participant number is presented as amount used in analysis, number between brackets represents total study participant number; ^1^risk estimate calculated based on information in original article.

Table S4.4.c, Studies on the association between cerebral microbleeds (CMBs) and all-cause mortality

| **Reference** | **Study population characteristics** | | | | | | | **MRI characteristics** | **CMBs assessment** | **Number of events** | **Outcome** | **Adjustments** |
| --- | --- | --- | --- | --- | --- | --- | --- | --- | --- | --- | --- | --- |
|  | **FU (y)** | **Study** | **N^a^** | **Study population** | **Country** | **Age (y)** | **Male (%)** |  |  |  |  |  |
| Akoudad et al., 2013 | 5.2 | RSS | 3,979 | Community-dwelling | Netherlands | 60.3 | 45.6 | 1.5T, T1, T2*, FLAIR, PD | Ql, Presence vs. absence | 172 | HR= 1.37 (0.96-1.94) | Age, sex, BP, HC, DM, smoking, antithrombotic use, centre |
| Altmann-Schneider et al., 2011 | 7.0 | PROSPER | 381 (435) | Stroke | Netherlands | 75.0 | 56.4 | 1.5T, T2, T2*, FLAIR | Ql, Presence ($\geq$2) vs. absence (0) | 137 | HR= 1.41 (0.87-2.27) | age, sex, BMI, HC, BP, DM, CVD, TIA, smoking, alcohol, statin use |
| Benedictus et al., 2015 | 3-6 | MISTRAL | 333 | AD | Netherlands | 72.2 | 58.0 | 1T-3T, T1, T2, T2*, FLAIR | Ql, Presence vs. absence | 147 | HR= 1.70 (1.20-2.40) | Age, sex, MMSE, cardiovascular risk factors, WMHs, lacunes |
| Boulanger et al., 2006 | NA | Vision | 236 | Stroke | Canada | NA | 55.1 | 3T, T1, T2, T2*, FLAIR | Ql, Presence vs. absence | 20 | HR= 3.10 (1.20-7.80) | Age, WMHs |
| Fan et al., 2003 | 2.3 | NA | 121 | Stroke | China | 68.0 | 67.8 | 1.5T, T1, T2, T2* | Ql, Presence vs. absence | 14 | OR= 1.01 (0.32-3.23) | None |
| Haji et al., 2015 | 2.4 | NA | 134 | Stroke | USA | 79.9 | 53.0 | 1.5 - 3T | Ql, Presence vs. absence | 64 | OR= 1.22 (0.57-2.60) | None |
| Henneman et al., 2009 | 2.6 | NA | 228 | MCI | Netherlands | 66 .0 | 55.2 | 1-1.5T, T1, T2, T2*, FLAIR | Ql, categorical , presence ($\geq$3) vs. absence (0) | 153 | HR= 2.00 (1.50-2.60) per microbleed  HR= 2.40 (1.40-4.30) for presence of CMBs | Age, sex, HC, BP, DM, CVD |
| Kwa et al., 2012 | 3.8 | ESPRIT | 397 | Stroke | Netherlands | 65.3 | 58.4 | 1.0T, 1.5T, T2* | Ql, Presence vs. absence | 40 | HR= 1.60 (0.80-3.30) | Age, sex |
| Mok et al., 2009 | 5.0 | NA | 259 | Stroke | China | 70.7 | 52.0 | 1.5T, T1, T2, DWI | Ql, Presence vs. absence | 16 | HR= 0.55 (0.17-1.83) | Age |
| van der Holst et al., 2016 | 7.8 | RUNDMC | 630 (655) | Community-dwelling | Netherlands | 65.7 | 56.5 | 1.5T, T1, T2*, FLAIR, DTI | Ql, Presence vs. absence | 80 | OR= 1.93 (1.17-3.17)^1^ | None |

*Tables S4.4.c*. Summary of study characteristics. Abbreviations: AD: Alzheimer’s disease; BP: blood pressure; BMI: body mass index; CMBs: cerebral microbleeds; CVD: cardiovascular disease; DM: diabetes mellitus; ESPRIT: European/Australasian Stroke Prevention in Reversible Ischaemia Trial; FLAIR: fluid attenuation inversion recovery; FU: follow-up; HC: hypercholesterolemia; HR: hazard ratio; MISTRAL: do MIcrobleeds predict STRoke in ALzheimer's disease Study; MRI: magnetic resonance imaging; NA: not available; OR: odds ratio; PD: proton density; PROSPER: PROspective Study of Pravastatin in the Elderly at Risk; Ql: qualitative; RSS: Rotterdam Scan Study; RUNDMC: Radboud University Nijmegen Diffusion Tensor and Magnetic Resonance Cohort; TIA: transient ischaemic attack; USA: United States of America; WMHs: white matter hyperintensities. ^a^Participant number is presented as amount used in analysis, number between brackets represents total study participant number; ^1^risk estimate calculated based on information in original article.

Table S4.4.d, Studies on the association between total cerebral atrophy and all-cause mortality

| **Reference** | **Study population characteristics** | | | | | | | **MRI characteristics** | **Cerebral atrophy assessment** | **Number of events** | **Outcome** | **Adjustments** |
| --- | --- | --- | --- | --- | --- | --- | --- | --- | --- | --- | --- | --- |
|  | **FU (y)** | **Study** | **N^a^** | **Study population** | **Country** | **Age (y)** | **Male (%)** |  |  |  |  |  |
| Firbank et al., 2012a | 3.2 | NA | 106 | Stroke | UK | 79.8 | 53.8 | 1.5T, T1, FLAIR | Qt, continuous (per mL, per SD) | 60 | HR= 1.00 (0.99-1.00) per mL higher TCV  HR= 1.28 (1.00-1.78) per SD decrease in TCV^1^ | None |
| Staff et al., 2010 | 6.0 | NA | 98 | Community-dwelling | UK | 78.0 | 57.1 | 1T, T1, T2 | Qt, dichotomised (1^st^ vs. 2^nd^-4^th^ quartile) | 37 | HR= 2.77 (1.05-7.31) for 1^st^ quartile TCV | None |
| van der Veen et al., 2014 | 8.3 | SMART-MR | 1,215 | With cardiovascular disease(s) | Netherlands | 58.0 | 80.0 | 1.5T, T1, T2, FLAIR | Qt, continuous (per SD) | 184 | HR= 1.46 (1.22-1.76) per SD decrease in TCV | Age, sex, BMI, BP, DM, smoking, alcohol, intima media thickness |

*Tables S4.4.d*. Summary of study characteristics. Abbreviations: BP: blood pressure; BMI: body mass index; DM: diabetes mellitus; FLAIR: fluid attenuation inversion recovery; FU: follow-up; HR: hazard ratio; mL: milliliter; MRI: magnetic resonance imaging; NA: not available; Qt: quantitative; SD: standard deviation; SMART-MR: Second Manifestations of ARTerial disease-Magnetic Resonance; TCV: total cerebral volume; UK: United Kingdom . ^a^Participant number is presented as amount used in analysis, number between brackets represents total study participant number^1^risk estimate calculated based on information in original article.

**Table S5.1 – Newcastle-Ottawa Scale scores for studies on the association between cerebral small vessel disease and incident ischaemic and haemorrhagic stroke**

| Study | S1 | S2 | S3 | C1 | O1 | O2 | O3 | Total score |
| --- | --- | --- | --- | --- | --- | --- | --- | --- |
| **Akoudad et al., 2015** | 1 | 1 | 0 | 2 | 1 | 1 | 1 | **7** |
| Andersen et al., 2016 | 0 | 1 | 0 | 1 | 1 | 0 | 1 | **4** |
| Andersen et al., 2017 | 0 | 1 | 0 | 0 | 1 | 1 | 1 | **4** |
| Appelros et al., 2005 | 0 | 0 | 0 | 0 | 1 | 0 | 1 | **2** |
| Benedictus et al., 2015 | 0 | 0 | 0 | 1 | 1 | 0 | 1 | **3** |
| **Bernick et al., 2001** | 1 | 1 | 1 | 1 | 0 | 1 | 0 | **5** |
| Bokura et al., 2011 | 1 | 0 | 1 | 1 | 0 | 0 | 1 | **4** |
| **Bokura et al., 2006** | 1 | 0 | 0 | 2 | 1 | 1 | 0 | **5** |
| Boulanger et al., 2006 | 0 | 1 | 0 | 0 | 0 | 0 | 1 | **2** |
| **Buyck et al., 2009** | 1 | 1 | 1 | 2 | 1 | 1 | 1 | **8** |
| **Conijn et al., 2011** | 0 | 1 | 0 | 2 | 1 | 1 | 1 | **6** |
| **Debette et al., 2010** | 1 | 0 | 1 | 2 | 1 | 1 | 0 | **6** |
| Fan et al., 2003 | 0 | 1 | 0 | 0 | 0 | 0 | 1 | **2** |
| Fu et al., 2005 | 0 | 1 | 0 | 0 | 0 | 0 | 1 | **2** |
| Gerdes et al., 2006 | 0 | 1 | 0 | 0 | 0 | 0 | 0 | **1** |
| Gioia et al., 2012 | 0 | 1 | 0 | 2 | 0 | 0 | 0 | **3** |
| Haji et al., 2015 | 0 | 0 | 0 | 0 | 0 | 0 | 0 | **0** |
| Imaizumi et al., 2015b | 0 | 1 | 0 | 0 | 0 | 0 | 1 | **2** |
| Inzitari et al., 2009 | 0 | 0 | 0 | 0 | 1 | 0 | 0 | **1** |
| Ishikawa et al., 2007 | 1 | 1 | 1 | 0 | 0 | 0 | 0 | **3** |
| **Kaffashian et al., 2016a** | 1 | 1 | 1 | 2 | 1 | 1 | 1 | **8** |
| **Kaffashian et al., 2016b** | 1 | 1 | 1 | 2 | 1 | 1 | 1 | **8** |
| **Kario et al., 2001** | 0 | 1 | 1 | 1 | 1 | 0 | 1 | **5** |
| Kobayashi et al., 1997 | 1 | 0 | 1 | 0 | 0 | 0 | 1 | **3** |
| Kuller et al., 2004 | 1 | 0 | 1 | 1 | 1 | 0 | 0 | **4** |
| Kumral et al., 2015 | 0 | 0 | 0 | 0 | 0 | 1 | 1 | **2** |
| Kwa et al., 2012 | 0 | 0 | 0 | 1 | 0 | 0 | 1 | **2** |
| Melkas et al., 2012 | 0 | 0 | 0 | 1 | 1 | 1 | 0 | **3** |
| Mok et al., 2009 | 0 | 1 | 0 | 0 | 0 | 1 | 1 | **3** |
| Naganuma et al., 2013 | 0 | 1 | 1 | 0 | 0 | 0 | 1 | **3** |
| **Naganuma et al., 2015** | 0 | 1 | 1 | 2 | 0 | 1 | 1 | **6** |
| Naka et al., 2006 | 0 | 0 | 0 | 2 | 1 | 0 | 1 | **4** |
| **Nishikawa et al., 2009** | 1 | 1 | 1 | 1 | 1 | 0 | 0 | **5** |
| Ntaios et al., 2015 | 0 | 0 | 0 | 0 | 1 | 0 | 0 | **1** |
| **Poels et al., 2012** | 1 | 1 | 1 | 2 | 1 | 1 | 1 | **8** |
| Putaala et al., 2011 | 0 | 0 | 0 | 1 | 0 | 1 | 1 | **3** |
| Smith et al., 2004 | 0 | 0 | 0 | 0 | 0 | 0 | 0 | **0** |
| Soo et al., 2008 | 0 | 1 | 0 | 0 | 1 | 0 | 1 | **3** |
| Thijs et al., 2010 | 0 | 0 | 0 | 1 | 1 | 0 | 1 | **3** |
| **van der Veen et al., 2014** | 0 | 1 | 0 | 2 | 1 | 1 | 0 | **5** |
| Weber et al., 2012 | 0 | 0 | 0 | 0 | 0 | 0 | 0 | **0** |
| **Weinstein et al., 2013** | 1 | 0 | 1 | 2 | 1 | 1 | 0 | **6** |
| **Windham et al., 2015** | 1 | 1 | 1 | 2 | 1 | 1 | 1 | **8** |
| Yamauchi et al., 2002 | 0 | 0 | 0 | 2 | 1 | 1 | 0 | **4** |

Newcastle-Ottawa Scale score (NOS) for studies on the association between cerebral small vessel disease and incident haemorrhagic and ischaemic stroke. For an explanation of the individual items, see the provided adjusted NOS (Appendix C). Maximal NOS score is 8. Articles indicated in bold are of high methodologic quality (NOS score >4). S1= Representativeness of the cohort; S2 = ascertainment of determinant; S3= presence of outcome of interest at start of study; C1= Comparability of cohorts: use of adjustments; O1= assessment of outcome; O2= follow up duration; O3= adequacy of follow up.

**Table S5.2 – Newcastle-Ottawa Scale scores for studies on the association between cerebral small vessel disease and incident all-cause dementia**

| Study | S1 | S2 | S3 | C1 | O1 | O2 | O3 | Total score |
| --- | --- | --- | --- | --- | --- | --- | --- | --- |
| **Akoudad et al., 2016** | 1 | 1 | 1 | 2 | 1 | 0 | 1 | **7** |
| **Bombois et al., 2008** | 0 | 1 | 1 | 2 | 1 | 0 | 1 | **6** |
| **Debette et al., 2010** | 1 | 1 | 1 | 1 | 1 | 1 | 1 | **7** |
| DeCarli et al., 2004 | 0 | 0 | 0 | 0 | 0 | 0 | 1 | **1** |
| Firbank et al., 2012a | 0 | 1 | 1 | 0 | 1 | 0 | 0 | **3** |
| Geroldi et al., 2006 | 0 | 0 | 1 | 0 | 1 | 0 | 0 | **2** |
| **Godin et al., 2010** | 1 | 1 | 1 | 2 | 0 | 1 | 1 | **7** |
| Gomar et al., 2011 | 0 | 0 | 1 | 0 | 1 | 0 | 0 | **2** |
| **Ikram et al., 2010b** | 1 | 1 | 1 | 2 | 1 | 1 | 1 | **8** |
| **Kaffashian et al., 2016a** | 1 | 1 | 1 | 2 | 1 | 1 | 1 | **8** |
| **Kaffashian et al., 2016b** | 1 | 1 | 1 | 2 | 1 | 1 | 1 | **8** |
| **Kantarci et al., 2009** | 0 | 1 | 1 | 1 | 1 | 0 | 1 | **5** |
| **Kim et al., 2015** | 0 | 1 | 1 | 1 | 1 | 0 | 1 | **5** |
| **Kitagawa et al., 2015** | 1 | 1 | 1 | 2 | 1 | 1 | 0 | **7** |
| Korf et al., 2004 | 0 | 1 | 1 | 0 | 1 | 0 | 0 | **3** |
| **Kuller, 2003** | 1 | 1 | 1 | 1 | 0 | 1 | 0 | **5** |
| **Lopez et al., 2014** | 1 | 1 | 1 | 1 | 1 | 0 | 1 | **6** |
| Meguro et al., 2007 | 0 | 1 | 1 | 0 | 1 | 1 | 0 | 4 |
| **Miwa et al., 2014** | 0 | 1 | 1 | 1 | 1 | 1 | 0 | **5** |
| Prasad et al., 2011 | 0 | 0 | 1 | 0 | 1 | 0 | 0 | **2** |
| **Prins et al., 2004** | 1 | 1 | 1 | 0 | 1 | 1 | 1 | **6** |
| Prins et al., 2013 | 0 | 1 | 1 | 0 | 0 | 0 | 1 | **3** |
| **Rosano et al., 2007** | 1 | 1 | 1 | 1 | 1 | 1 | 0 | **6** |
| Sluimer et al., 2008 | 0 | 0 | 1 | 0 | 1 | 0 | 0 | **2** |
| Smith et al., 2008 | 0 | 1 | 1 | 0 | 1 | 1 | 0 | **4** |
| Staekenborg et al., 2009 | 0 | 0 | 1 | 0 | 1 | 0 | 0 | **2** |
| Steffens et al., 2002b | 0 | 0 | 1 | 0 | 1 | 0 | 0 | **2** |
| Steffens et al., 2007 | 0 | 0 | 1 | 0 | 1 | 1 | 0 | **3** |
| **Stephan et al., 2015** | 1 | 1 | 1 | 2 | 1 | 1 | 1 | **8** |
| **Stoub et al., 2014** | 1 | 1 | 1 | 0 | 1 | 1 | 0 | **5** |
| Tapiola et al., 2008 | 0 | 1 | 1 | 0 | 1 | 0 | 0 | **3** |
| van Straaten et al., 2008 | 0 | 1 | 1 | 0 | 1 | 0 | 0 | **3** |
| **van Uden et al., 2015** | 0 | 1 | 1 | 1 | 1 | 1 | 1 | **6** |
| Verdelho et al., 2010 | 0 | 1 | 1 | 0 | 1 | 0 | 0 | **3** |
| **Vermeer et al., 2003** | 1 | 1 | 1 | 1 | 1 | 0 | 1 | **6** |
| **Weinstein et al., 2013** | 1 | 1 | 1 | 2 | 1 | 1 | 0 | **7** |
| Yamamoto et al., 2002 | 0 | 1 | 1 | 0 | 1 | 1 | 0 | **4** |
| **Zhu et al., 2010** | 1 | 1 | 1 | 0 | 1 | 0 | 1 | **5** |

Newcastle-Ottawa Scale score (NOS) for studies on the association between cerebral small vessel disease and incident dementia. For an explanation of the individual items, see the provided adjusted NOS (Appendix C). Maximal NOS score is 8. Articles indicated with bold are of high methodologic quality (NOS score >4). S1= Representativeness of the cohort; S2 = ascertainment of determinant; S3= presence of outcome of interest at start of study; C1= Comparability of cohorts: use of adjustments; O1= assessment of outcome; O2= follow up duration; O3= adequacy of follow up.

**Table S5.3 – Newcastle-Ottawa Scale scores for studies on the association between cerebral small vessel disease and incident depression**

| Study | S1 | S2 | S3 | C1 | O1 | O2 | O3 | Total score |
| --- | --- | --- | --- | --- | --- | --- | --- | --- |
| **Godin et al., 2008** | 1 | 1 | 1 | 1 | 1 | 1 | 0 | **6** |
| **Ikram et al., 2009** | 1 | 1 | 1 | 1 | 1 | 1 | 1 | **7** |
| Kim et al., 2016 | 0 | 1 | 1 | 0 | 1 | 0 | 0 | **3** |
| **Park et al., 2015** | 1 | 1 | 1 | 1 | 1 | 0 | 0 | **5** |
| **Perez, 2013** | 1 | 1 | 1 | 1 | 1 | 0 | 1 | **6** |
| **Qiu et al., 2016** | 1 | 0 | 1 | 1 | 1 | 1 | 0 | **5** |
| **Steffens et al., 2002a** | 1 | 0 | 1 | 1 | 1 | 1 | 0 | **5** |
| Teodorczuk et al., 2007 | 0 | 1 | 1 | 0 | 1 | 0 | 1 | **4** |
| Teodorczuk et al., 2010 | 0 | 1 | 1 | 0 | 1 | 0 | 1 | **4** |
| **van Sloten et al., 2015** | 1 | 1 | 1 | 1 | 1 | 1 | 1 | **7** |
| **Versluis et al., 2006** | 1 | 1 | 1 | 1 | 1 | 0 | 1 | **6** |

Newcastle-Ottawa Scale score (NOS) for studies on the association between cerebral small vessel disease and incident depression. For an explanation of the individual items, see the provided adjusted NOS (Appendix C). Maximal NOS score is 8. Articles indicated with bold are of high methodologic quality (NOS score >4). S1= Representativeness of the cohort; S2 = ascertainment of determinant; S3= presence of outcome of interest at start of study; C1= Comparability of cohorts: use of adjustments; O1= assessment of outcome; O2= follow up duration; O3= adequacy of follow up.

**Table S5.4 – Newcastle-Ottawa Scale scores for studies on the association between cerebral small vessel disease and incident all-cause mortality**

| Study | S1 | S2 | S3 | C1 | O1 | O2 | O3 | Total score |
| --- | --- | --- | --- | --- | --- | --- | --- | --- |
| **Akoudad et al., 2015** | 1 | 1 | 1 | 2 | 1 | 1 | 1 | **8** |
| **Altmann-Schneider et al., 2011** | 0 | 1 | 1 | 2 | 1 | 1 | 1 | **7** |
| Andersen et al., 2016 | 0 | 1 | 1 | 1 | 0 | 0 | 1 | **4** |
| **Andersen et al., 2017** | 0 | 1 | 1 | 0 | 1 | 1 | 1 | **5** |
| Appelros et al., 2005 | 0 | 0 | 1 | 0 | 1 | 1 | 0 | **3** |
| Benedictus et al., 2015 | 0 | 0 | 1 | 1 | 1 | 0 | 1 | **4** |
| **Bokura et al., 2006** | 1 | 0 | 1 | 2 | 0 | 1 | 0 | **5** |
| Boulanger et al., 2006 | 0 | 1 | 1 | 0 | 0 | 0 | 1 | **3** |
| **Conijn et al., 2011** | 0 | 1 | 1 | 2 | 1 | 1 | 1 | **7** |
| **Debette et al., 2010** | 1 | 0 | 1 | 2 | 1 | 1 | 0 | **6** |
| Fan et al., 2003 | 0 | 1 | 1 | 0 | 0 | 0 | 1 | **3** |
| Firbank et al., 2012a | 0 | 1 | 1 | 0 | 0 | 0 | 0 | **2** |
| Fu et al., 2005 | 0 | 1 | 1 | 0 | 0 | 0 | 1 | **3** |
| Haji et al., 2015 | 0 | 0 | 1 | 0 | 1 | 0 | 0 | **2** |
| Henneman et al., 2009 | 0 | 1 | 1 | 1 | 1 | 0 | 0 | **4** |
| **Ikram et al., 2009** | 1 | 1 | 1 | 1 | 1 | 1 | 1 | **7** |
| Inzitari et al., 2009 | 0 | 0 | 1 | 0 | 0 | 0 | 0 | **1** |
| **Kerber et al., 2006** | 1 | 1 | 1 | 0 | 1 | 1 | 1 | **6** |
| **Kuller et al., 2007** | 1 | 0 | 1 | 2 | 1 | 1 | 1 | **7** |
| Kwa et al., 2012 | 0 | 0 | 1 | 1 | 0 | 0 | 1 | **3** |
| Lavretsky et al., 2010 | 0 | 1 | 1 | 0 | 0 | 1 | 0 | **3** |
| **Levy et al., 2003** | 0 | 1 | 1 | 1 | 1 | 1 | 0 | **5** |
| Mok et al., 2009 | 0 | 1 | 1 | 0 | 0 | 1 | 1 | **4** |
| **Oksala et al., 2009** | 0 | 0 | 1 | 1 | 2 | 1 | 1 | **6** |
| **Putaala et al., 2011** | 0 | 0 | 1 | 1 | 1 | 1 | 1 | **5** |
| Staff et al., 2010 | 1 | 0 | 1 | 0 | 1 | 1 | 0 | **4** |
| **van der Holst et al., 2016** | 1 | 1 | 1 | 2 | 1 | 1 | 1 | **8** |
| **van der Veen et al., 2014** | 0 | 1 | 1 | 2 | 1 | 1 | 0 | **6** |
| Weber et al., 2012 | 0 | 0 | 1 | 0 | 0 | 0 | 0 | **1** |
| **Windham et al., 2015** | 1 | 1 | 1 | 2 | 1 | 1 | 1 | **8** |
| Yamauchi et al., 2002 | 0 | 0 | 1 | 0 | 1 | 1 | 0 | **3** |

Newcastle-Ottawa Scale score (NOS) for studies on the association between cerebral small vessel disease and all-cause mortality. For an explanation of the individual items, see the provided adjusted NOS (Appendix C). Maximal NOS score is 8. Articles indicated with bold are of high methodologic quality (NOS score >4). S1= Representativeness of the cohort; S2 = ascertainment of determinant; S3= presence of outcome of interest at start of study; C1= Comparability of cohorts: use of adjustments; O1= assessment of outcome; O2= follow up duration; O3= adequacy of follow up.

**Table S6.1 – Heterogeneity for analyses with incident ischaemic and haemorrhagic stroke**


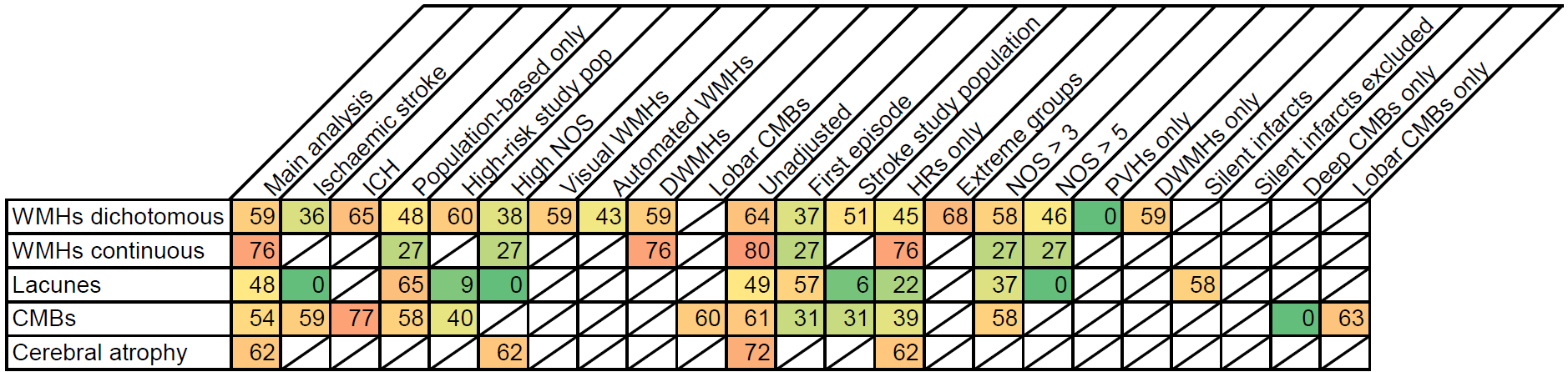


Heterogeneity (I^2^) in the main and sensitivity analyses for the association of cerebral small vessel disease with incident ischaemic and haemorrhagic stroke. For a description of the sensitivity analyses, see Figures S2.1 to S2.5. Abbreviations: CMBs: cerebral microbleeds; DWMHs: deep white matter hyperintensities; HRs: hazard ratios; ICH: intracerebral haemorrhage; NOS: Newcastle-Ottawa scale score; pop: population; PVHs: periventricular hyperintensities; WMHs: white matter hyperintensities.

**Table S6.2 – Heterogeneity for analyses with incident all-cause dementia**


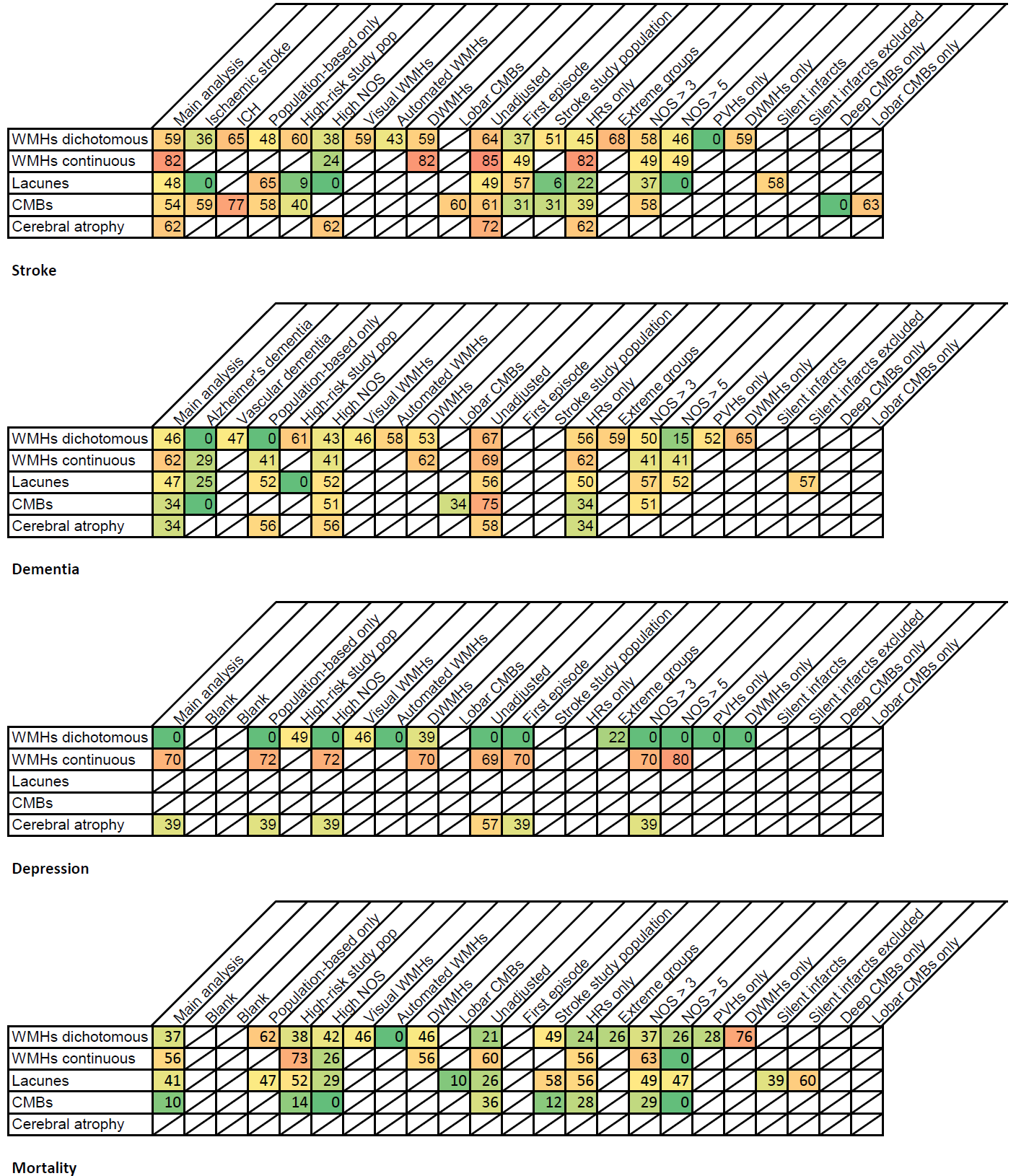
Heterogeneity (I^2^) in the main and sensitivity analyses for the association of cerebral small vessel disease with incident all-cause dementia. For a description of the sensitivity analyses, see Figures S2.1 to S2.5. Abbreviations: CMBs: cerebral microbleeds; DWMHs: deep white matter hyperintensities; HRs: hazard ratios; ICH: intracerebral haemorrhage; NOS: Newcastle-Ottawa scale score; pop: population; PVHs: periventricular hyperintensities; WMHs: white matter hyperintensities.

**Table S6.3 – Heterogeneity for analysis with incident depression**

**
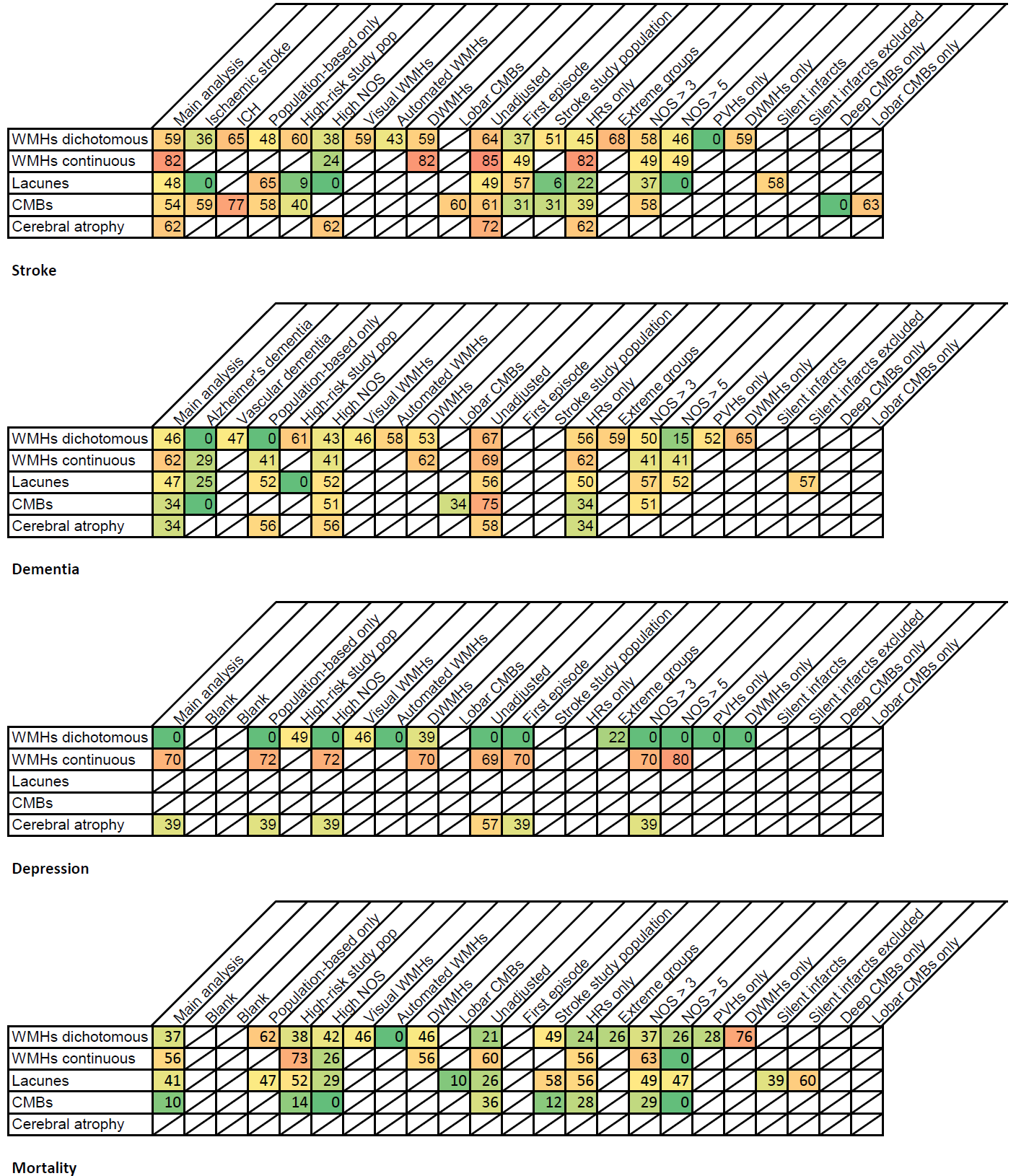
**

Heterogeneity (I^2^) in the main and sensitivity analyses for the association of cerebral small vessel disease with incident depression. For a description of the sensitivity analyses, see Figures S2.1 to S2.5. Abbreviations: CMBs: cerebral microbleeds; DWMHs: deep white matter hyperintensities; HRs: hazard ratios; ICH: intracerebral haemorrhage; NOS: Newcastle-Ottawa scale score; pop: population; PVHs: periventricular hyperintensities; WMHs: white matter hyperintensities.

**Table S6.4 – Heterogeneity for analysis with all-cause mortality**


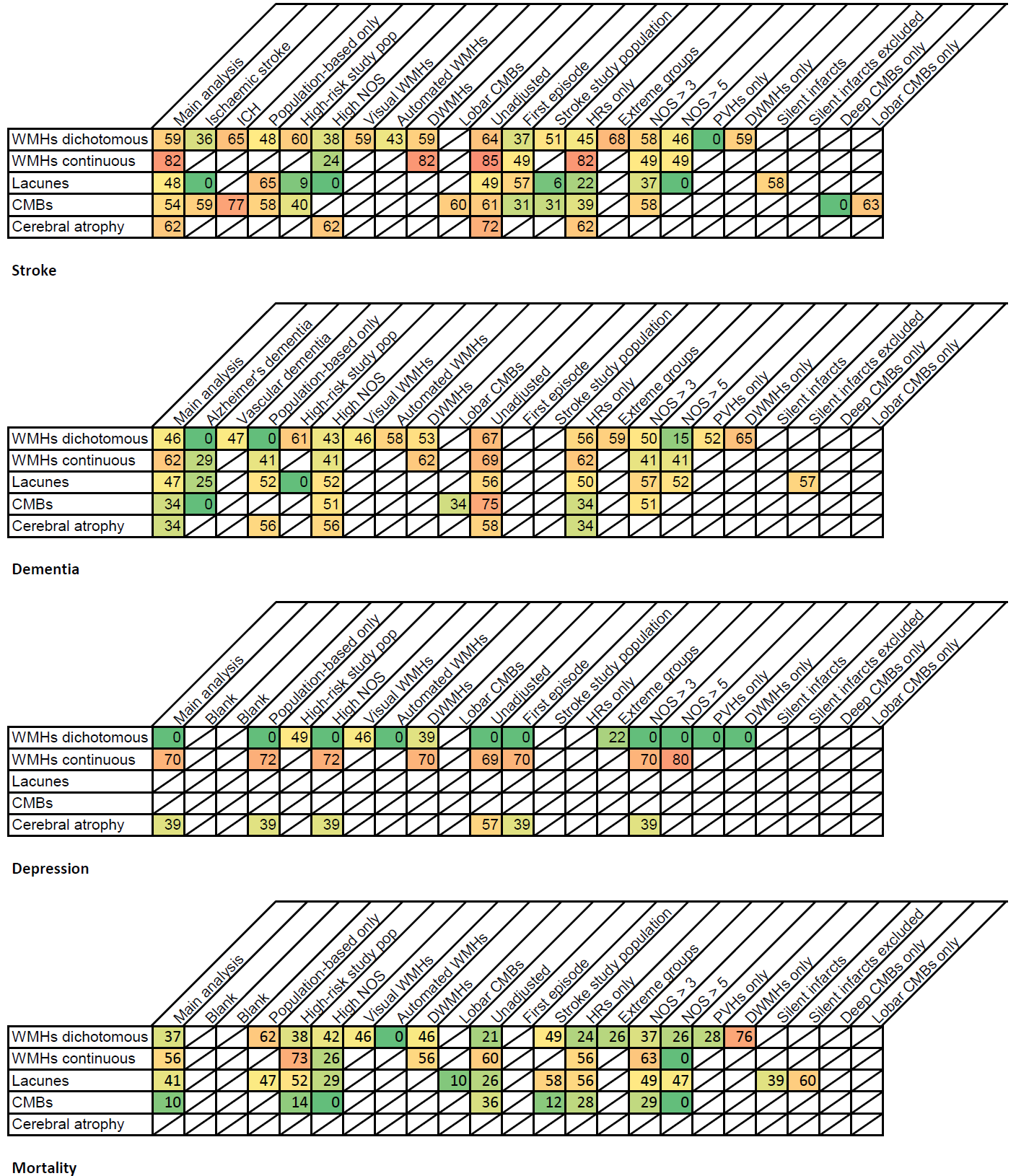
Heterogeneity (I^2^) in the main and sensitivity analyses for the association of cerebral small vessel disease with all-cause mortality. For a description of the sensitivity analyses, see Figures S2.1 to S2.5. Abbreviations: CMBs: cerebral microbleeds; DWMHs: deep white matter hyperintensities; HRs: hazard ratios; ICH: intracerebral haemorrhage; NOS: Newcastle-Ottawa scale score; pop: population; PVHs: periventricular hyperintensities; WMHs: white matter hyperintensities.

**Table S7 –Egger’s Tests to detect significant funnel plot asymmetry**

| Association | T-value | df | p-value |
| --- | --- | --- | --- |
| WMHs on a dichotomous scale with ischaemic or haemorrhagic stroke | 4.70 | 21 | 0.0001 |
| Lacunes with ischaemic or haemorrhagic stroke | 2.55 | 13 | 0.02 |
| CMBs with ischaemic or haemorrhagic stroke | 3.01 | 13 | 0.01 |
| WMHs on a dichotomous scale with all-cause dementia | 0.22 | 13 | 0.83 |
| Lacunes with all-cause dementia | 2.00 | 8 | 0.08 |
| WMHs on a dichotomous scale with all-cause mortality | 2.33 | 14 | 0.04 |
| Lacunes with all-cause mortality | -0.33 | 9 | 0.75 |
| CMBs with all-cause mortality | -0.56 | 8 | 0.59 |

Tests were done when more than 10 studies were available per analysis. Abbreviations: CMBs: cerebral microbleeds; df: degrees of freedom; WMHs: white matter hyperintensities.

**Table S8- Trim and fill test for analyses with significant funnel plot asymmetry**

| Association | Estimated hazard ratio | Estimated 95% CI interval | | Estimated number of missing studies |
| --- | --- | --- | --- | --- |
|  |  | Lower limit | Upper limit |  |
| WMHs on a dichotomous scale with stroke | 1.94 | 1.54 | 2.45 | 7 |
| Lacunes with stroke | 2.36 | 1.84 | 3.02 | 4 |
| CMBs with stroke | 1.62 | 1.23 | 2.13 | 4 |
| WMHs on a dichotomous scale with mortality | 1.68 | 1.48 | 1.90 | 4 |

Abbreviations: CI: confidence interval; CMBs: cerebral microbleeds; WMHs: white matter hyperintensities.

**Figure S1.1a, Forest plot for the association between white matter hyperintensities on a dichotomous scale and incident ischaemic and haemorrhagic stroke**


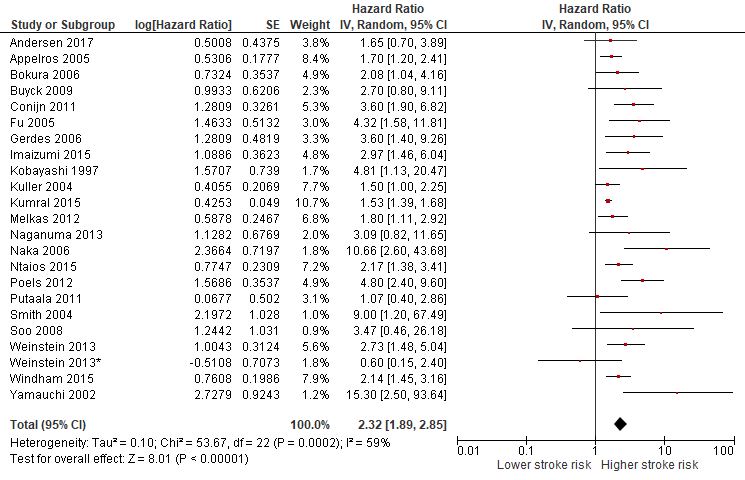


*data from the original cohort of the Framingham Health Study, the main study used data from the Framingham Offspring study.

**Figure S1.1b, Forest plot for the association between white matter hyperintensities on a continuous scale and incident ischaemic and haemorrhagic stroke**


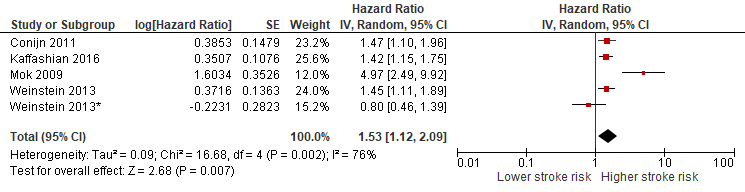


*data from the original cohort of the Framingham Health Study, the main study used data from the Framingham Offspring study.

**Figure S1.1c, Forest plot for the association between lacunes and incident ischaemic and haemorrhagic stroke**


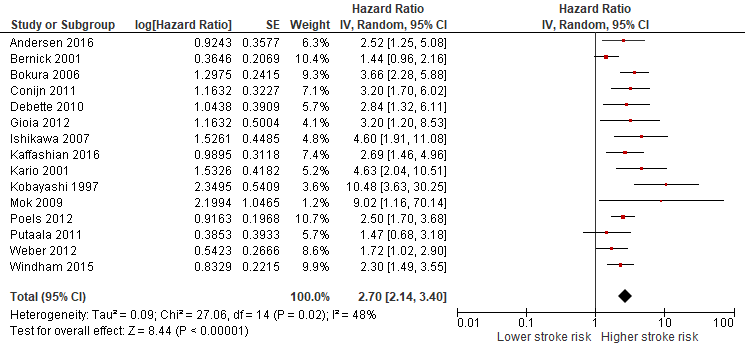


**Figure S1.1d, Forest plot for the association between cerebral microbleeds and incident ischaemic and haemorrhagic stroke**


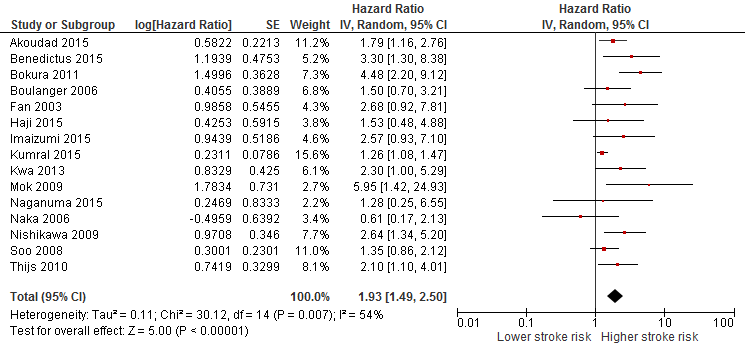


**Figure S1.1e, Forest plot for the association between total cerebral atrophy and incident ischaemic and haemorrhagic stroke**


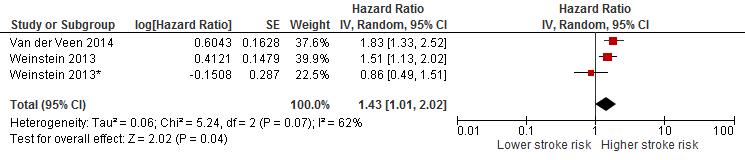


*data from the original cohort of the Framingham Health Study, the main study used data from the Framingham Offspring study.

**Figure S1.1f, Forest plot for the association between combinations of cerebral small vessel disease features and incident ischaemic and haemorrhagic stroke**


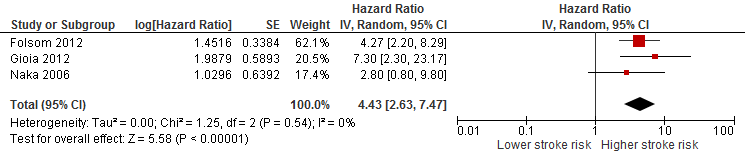


**Figure S1.2a, Forest plot for the association between white matter hyperintensities on a dichotomous scale and incident all-cause dementia**


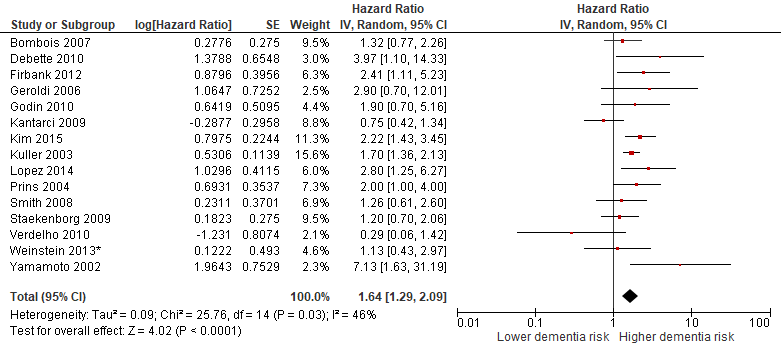


*data from the original cohort of the Framingham Health Study, the main study used data from the Framingham Offspring study.

**Figure S1.2b, Forest plot for the association between white matter hyperintensities on a continuous scale and incident all-cause dementia**

**
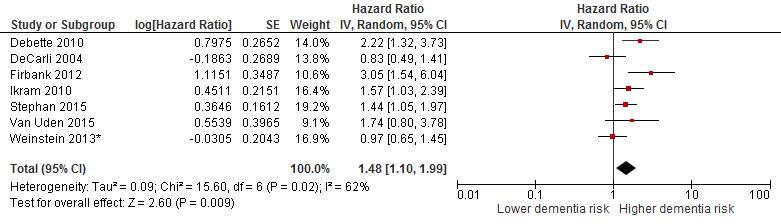
**

*data from the original cohort of the Framingham Health Study, the main study used data from the Framingham Offspring study.

**Figure S1.2c, Forest plot for the association between lacunes and incident all-cause dementia**

**
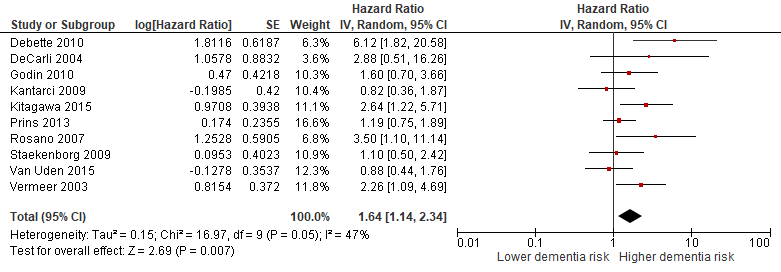
**

**
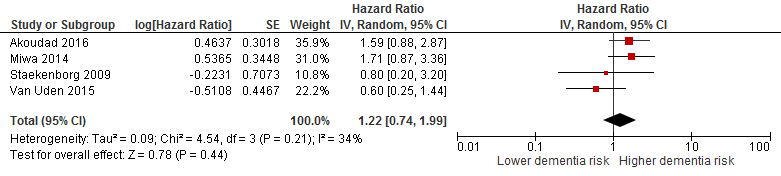
Figure S1.2d, Forest plot for the association between cerebral microbleeds and incident all-cause dementia**

**Figure S1.2e, Forest plot for the association between total cerebral atrophy and incident all-cause dementia**


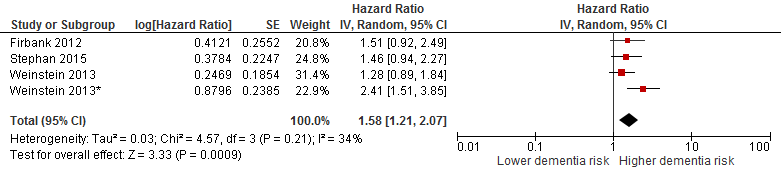


*data from the original cohort of the Framingham Health Study, the main study used data from the Framingham Offspring study.

**Figure S1.3a, Forest plot for the association between white matter hyperintensities on a dichotomous scale and incident depression**

**
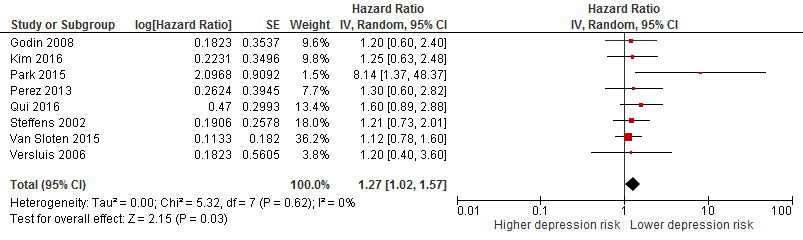
**

**Figure S1.3b, Forest plot for the association between white matter hyperintensities on a continuous scale and incident depression**

**
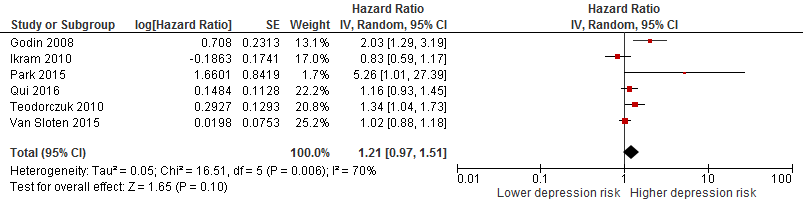
**

**Figure S1.3c, Forest plot for the association between total cerebral atrophy and incident depression**
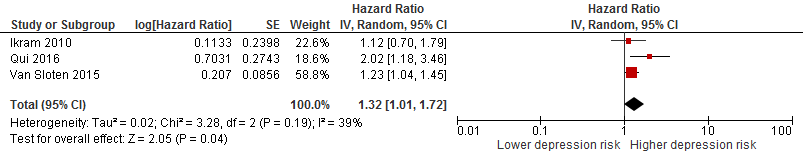


**Figure S1.4a, Forest plot for the association between white matter hyperintensities on a dichotomous scale and all-cause mortality**

**
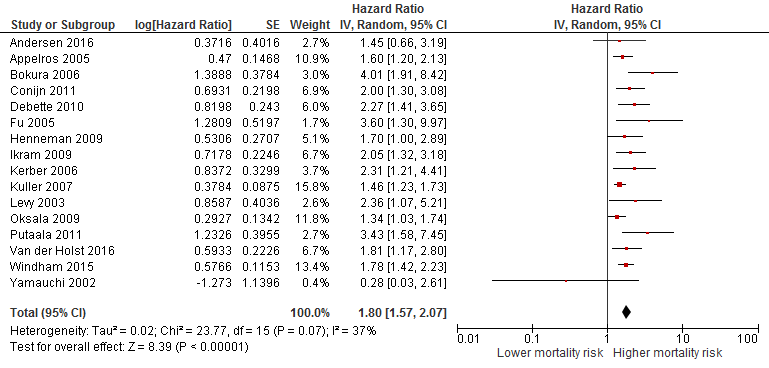
**

**Figure S1.4b, Forest plot for the association between white matter hyperintensities on a continuous scale and all-cause mortality
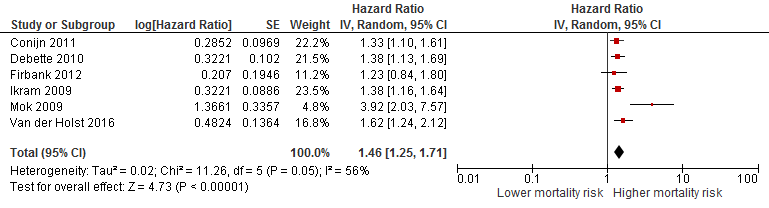
**

**Figure S1.4c, Forest plot for the association between lacunes and all-cause mortality
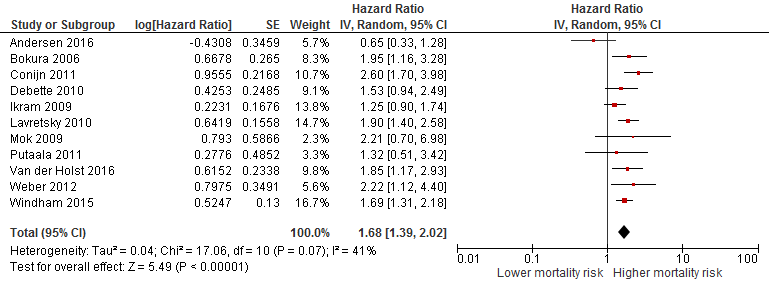
**

**Figure S1.4d, Forest plot for the association between cerebral microbleeds and all-cause mortality**
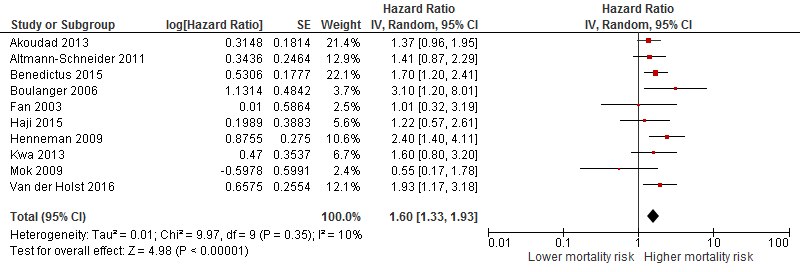


*Figures S1.1 to S1.4.* Forest plots for the associations between features of cerebral small vessel disease and incident ischaemic and haemorrhagic stroke, all-cause dementia and depression, and all-cause mortality. Abbreviations: CI: confidence interval; df: degrees of freedom; IV: inverse variance; SE: standard error;

**Figure S2.1 – Pooled hazard ratios for sensitivity analyses for white matter hyperintensities on a dichotomous scale (High vs. low)***

**
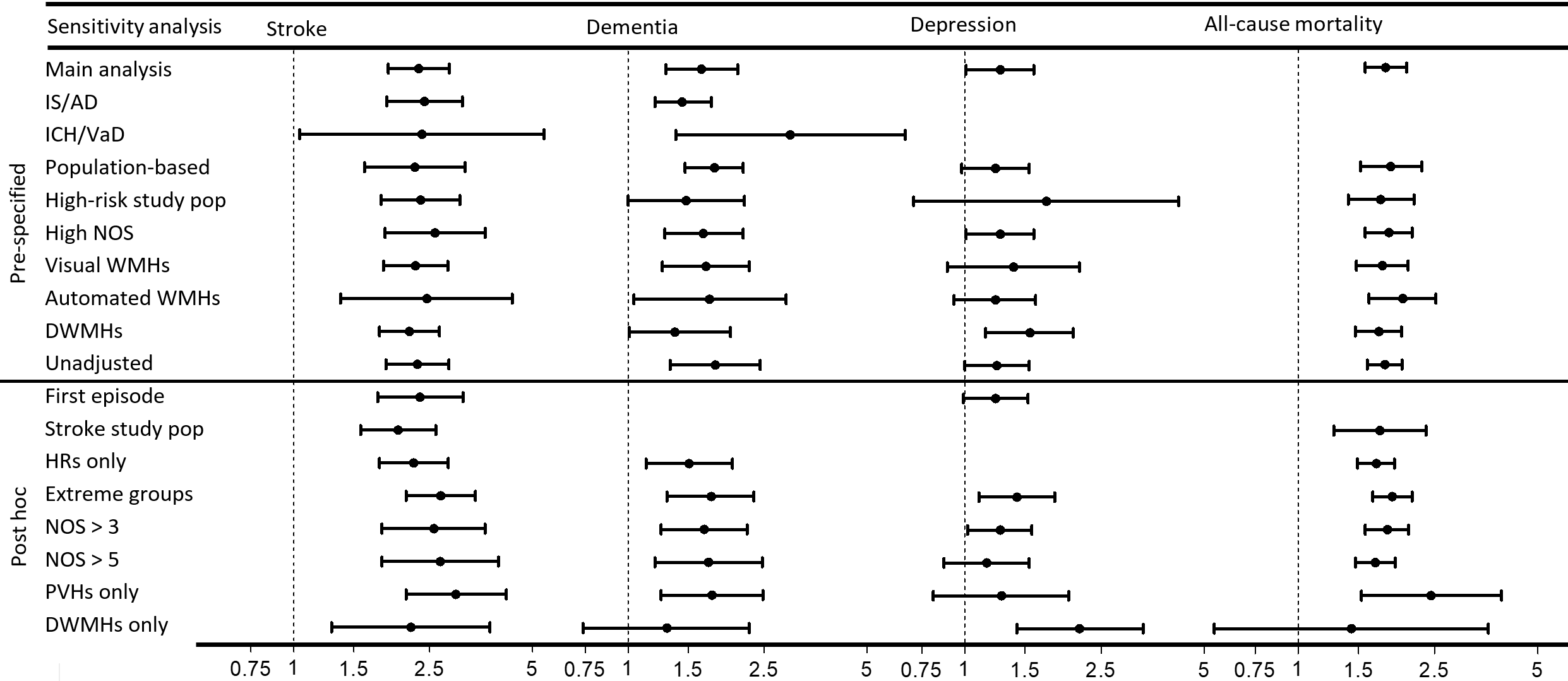
**

*Figures S2.1.* Pooled hazard ratios (HRs) (95% confidence intervals) for the association between features of white matter hyperintensities on a dichotomous scale and incident ischaemic and haemorrhagic stroke, all-cause dementia, depression, and all-cause mortality. *High vs. low as defined by the individual studies. Results were pooled when at least 3 studies were available. The following pre-specified analyses were done: analyses were repeated using only subtypes of stroke (ischaemic and haemorrhage) or dementia (Alzheimer’s disease and presumed vascular dementia) as the outcome; using only population-based cohort studies; using only studies with high-risk populations; using only high-quality studies (defined as Newcastle-Ottawa Scale (NOS) score >4); using only studies that measured white matter hyperintensities (WMHs) on an observer-rated semi-quantitative scale; using only studies that measured WMHs on an automated quantitative scale; replacing the risk estimates for periventricular WMHs with those for deep WMHs; and replacing adjusted risk estimates with unadjusted risk estimates. The following post hoc analyses were done: analyses were repeated using only studies with a first episode of stroke or depression; using only studies with stroke patients; using only hazard ratios (i.e. excluding studies that reported odds ratios or relative risks); using risk estimates comparing highest vs. lowest categories of WMHs (irrespective of the number of participants per category) (indicated in the figures as “extreme groups”), instead of risk estimates comparing higher and lower categories with the highest number of participants and events; using only studies with NOS score >3; using only studies with NOS score >5; using only risk estimates of periventricular white matter hyperintensities (PVHs); and using only risk estimates of deep white matter hyperintensities (DWMHs)**.** Abbreviations: AD: Alzheimer’s disease; CMBs: cerebral microbleeds; DWMHs: deep white matter hyperintensities; HRs: hazard ratios; ICH: intracerebral haemorrhage; IS: ischaemic stroke; NOS: Newcastle-Ottawa scale score; pop: population; PVHs: periventricular hyperintensities; VaD: presumed vascular dementia; WMHs: white matter hyperintensities.

**Figure S2.2 – Pooled hazard ratios for sensitivity analyses for white matter hyperintensities on a continuous scale (per 1 SD increase)**

**
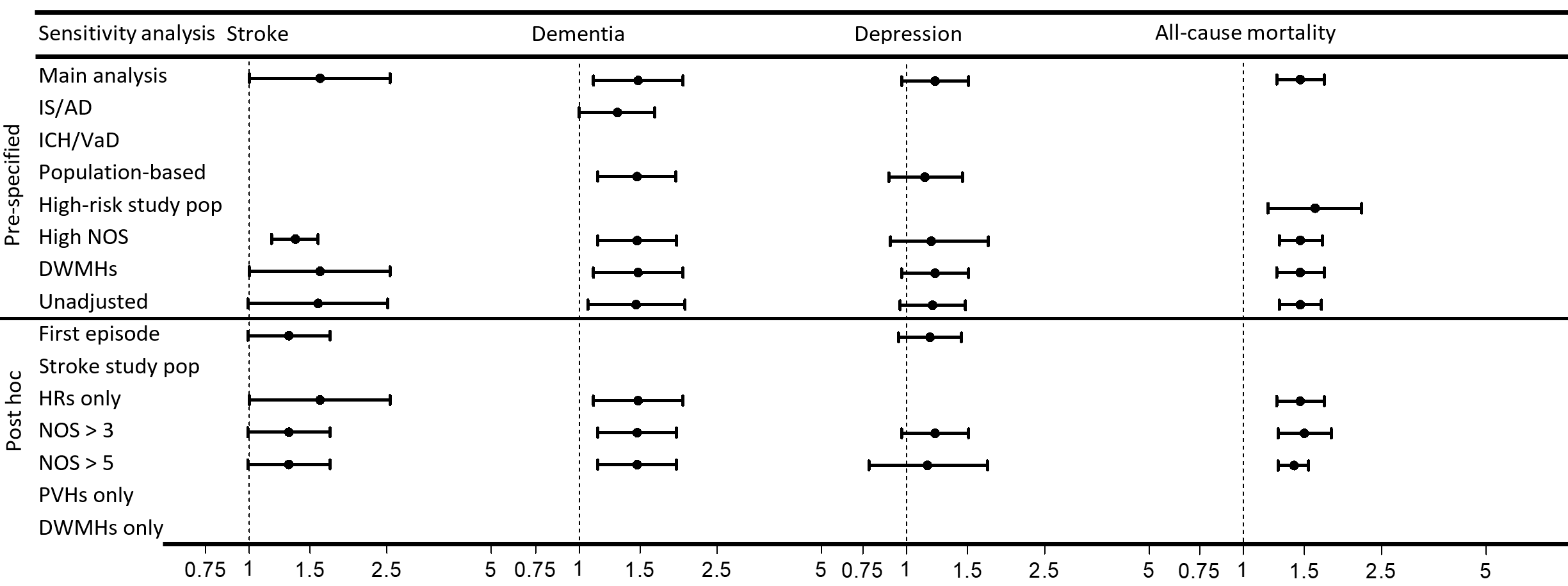
**

*Figures S2.2.* Pooled hazard ratios (HRs) (95% confidence intervals) for the association between white matter hyperintensities on a continuous scale and incident ischaemic and haemorrhagic stroke, all-cause dementia, depression, and all-cause mortality. Results were pooled when at least 3 studies were available. The following pre-specified analyses were done: analyses were repeated using only subtypes of stroke (ischaemic and haemorrhage) or dementia (Alzheimer’s disease and presumed vascular dementia) as the outcome; using only population-based cohort studies; using only studies with high-risk populations; using only high-quality studies (defined as Newcastle-Ottawa Scale (NOS) score >4); replacing the risk estimates for periventricular WMHs with those for deep WMHs; and replacing adjusted risk estimates with unadjusted risk estimates. The following post hoc analyses were done: analyses were repeated using only studies with a first episode of stroke or depression; using only studies with stroke patients; using only hazard ratios (i.e. excluding studies that reported odds ratios or relative risks); using only studies with NOS score >3; using only studies with NOS score >5; using only risk estimates of periventricular white matter hyperintensities (PVHs); and using only risk estimates of deep white matter hyperintensities (DWMHs)**.** Abbreviations: AD: Alzheimer’s disease; DWMHs: deep white matter hyperintensities; HRs: hazard ratios; ICH: intracerebral haemorrhage; IS: ischaemic stroke; NOS: Newcastle-Ottawa scale score; pop: population; PVHs: periventricular hyperintensities; SD: standard deviation; VaD: presumed vascular dementia; WMHs: white matter hyperintensities.

**Figure S2.3 – Pooled hazard ratios for sensitivity analyses for lacunes (absence vs. presence)**

**
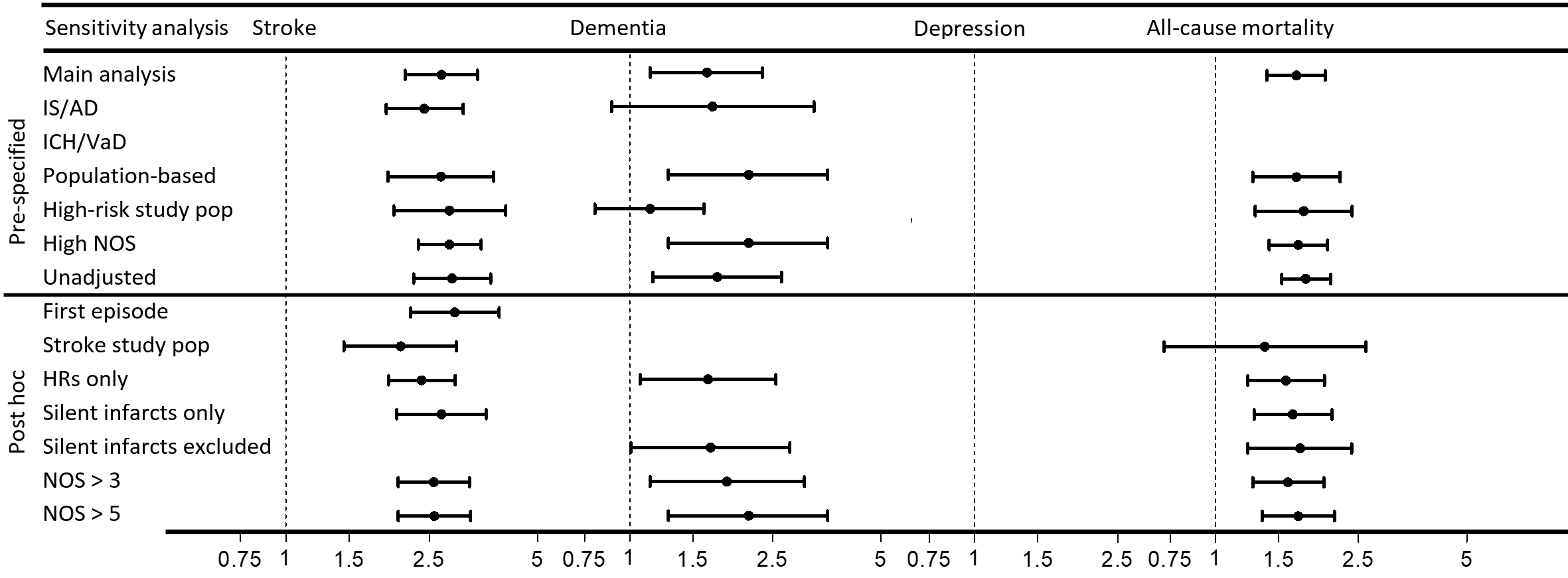
**

*Figures S2.3.* Pooled hazard ratios (HRs) (95% confidence intervals) for the association between lacunes and incident ischaemic and haemorrhagic stroke, all-cause dementia, depression, and all-cause mortality. Results were pooled when at least 3 studies were available. The following pre-specified analyses were done: analyses were repeated using only subtypes of stroke (ischaemic and haemorrhage) or dementia (Alzheimer’s disease and presumed vascular dementia) as the outcome; using only population-based cohort studies; using only studies with high-risk populations; using only high-quality studies (defined as Newcastle-Ottawa Scale (NOS) score >4); and replacing adjusted risk estimates with unadjusted risk estimates. The following post hoc analyses were done: analyses were repeated using only studies with a first episode of stroke or depression; using only studies with stroke patients; using only hazard ratios (i.e. excluding studies that reported odds ratios or relative risks); using only risk estimates for silent infarcts; excluding studies that reported risk estimates for silent infarcts only; using only studies with NOS score >3; and using only studies with NOS score >5**.** Abbreviations: AD: Alzheimer’s disease; HRs: hazard ratios; ICH: intracerebral haemorrhage; IS: ischaemic stroke; NOS: Newcastle-Ottawa scale score; pop: population; VaD: presumed vascular dementia.

**Figure S2.4 – Pooled hazard ratios for sensitivity analyses for cerebral microbleeds (absence vs. presence)**

**
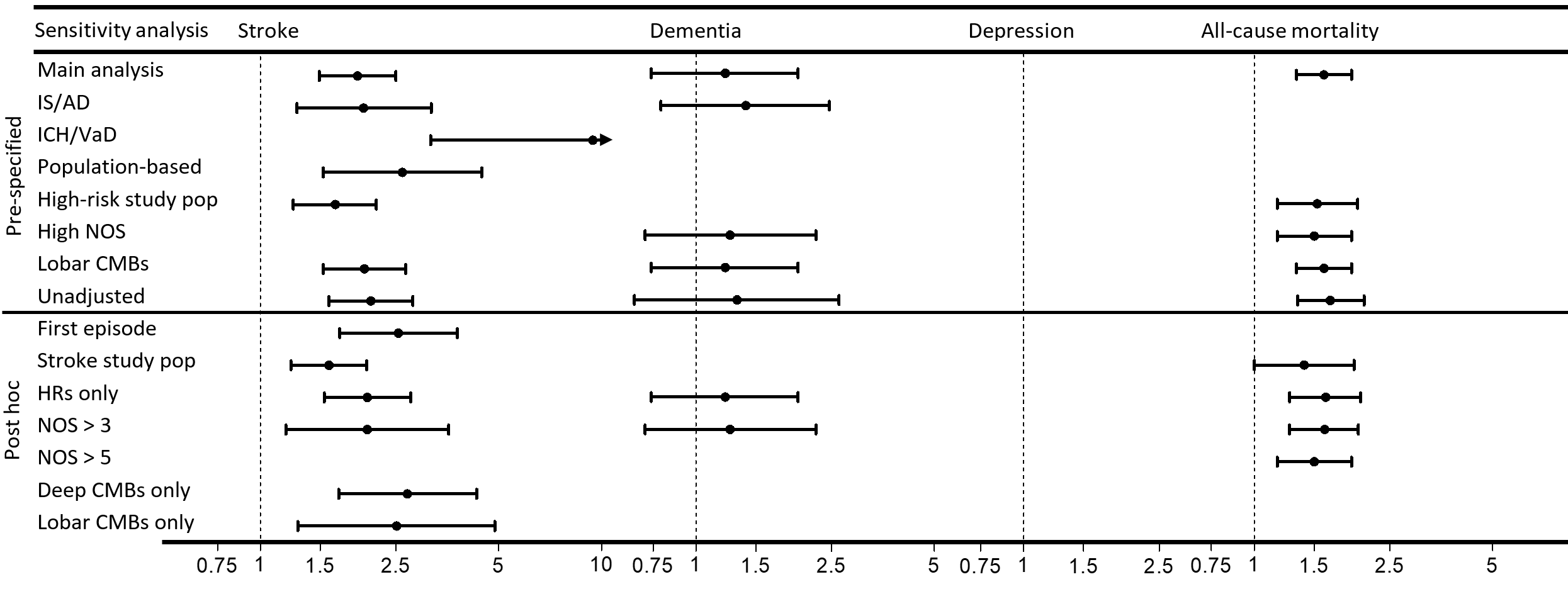
**

*Figures S2.4.* Pooled hazard ratios (HRs) (95% confidence intervals) for the association between cerebral microbleeds and incident ischaemic and haemorrhagic stroke, all-cause dementia, depression, and all-cause mortality. Results were pooled when at least 3 studies were available. The following pre-specified analyses were done: analyses were repeated using only subtypes of stroke (ischaemic and haemorrhage) or dementia (Alzheimer’s disease and presumed vascular dementia) as the outcome; using only population-based cohort studies; using only studies with high-risk populations; using only high-quality studies (defined as Newcastle-Ottawa Scale (NOS) score >4); replacing the risk estimates for deep cerebral microbleeds (CMBs) with those for lobar CMBs; and replacing adjusted risk estimates with unadjusted risk estimates. The following post hoc analyses were done: analyses were repeated using only studies with a first episode of stroke or depression; using only studies with stroke patients; using only hazard ratios (i.e. excluding studies that reported odds ratios or relative risks); using only studies with NOS score >3; using only studies with NOS score >5; using only risk estimates for deep CMBs; and using only risk estimates for lobar CMBs**.** Abbreviations: AD: Alzheimer’s disease; CMBs: cerebral microbleeds; HRs: hazard ratios; ICH: intracerebral haemorrhage; IS: ischaemic stroke; NOS: Newcastle-Ottawa scale score; pop: population; VaD: presumed vascular dementia.

**Figure S2.5 – Pooled hazard ratios for sensitivity analyses for total cerebral atrophy (per 1 SD decrease)**

*
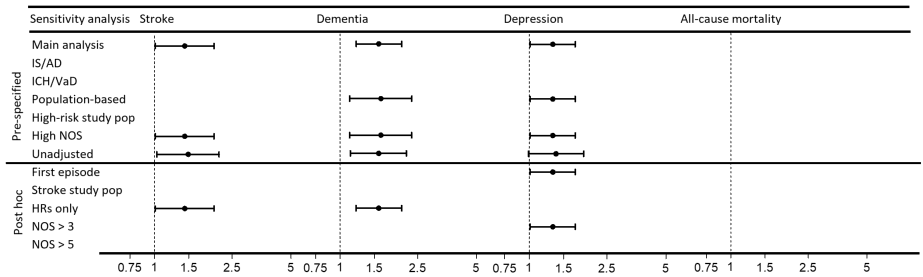
*

*Figures S2.5.* Pooled hazard ratios (HRs) (95% confidence intervals) for the association between cerebral atrophy and incident ischaemic and haemorrhagic stroke, all-cause dementia, depression, and all-cause mortality. Results were pooled when at least 3 studies were available. The following pre-specified analyses were done: analyses were repeated using only subtypes of stroke (ischaemic and haemorrhage) or dementia (Alzheimer’s disease and presumed vascular dementia) as the outcome; using only population-based cohort studies; using only studies with high-risk populations; using only high-quality studies (defined as Newcastle-Ottawa Scale (NOS) score >4); and replacing adjusted risk estimates with unadjusted risk estimates. The following post hoc analyses were done: analyses were repeated using only studies with a first episode of stroke or depression; using only studies with stroke patients; using only hazard ratios (i.e. excluding studies that reported odds ratios or relative risks); using only studies with NOS score >3; and using only studies with NOS score >5. Abbreviations: AD: Alzheimer’s disease; HRs: hazard ratios; ICH: intracerebral haemorrhage; IS: ischaemic stroke; NOS: Newcastle-Ottawa scale score; pop: population; SD: standard deviation; VaD: presumed vascular dementia.


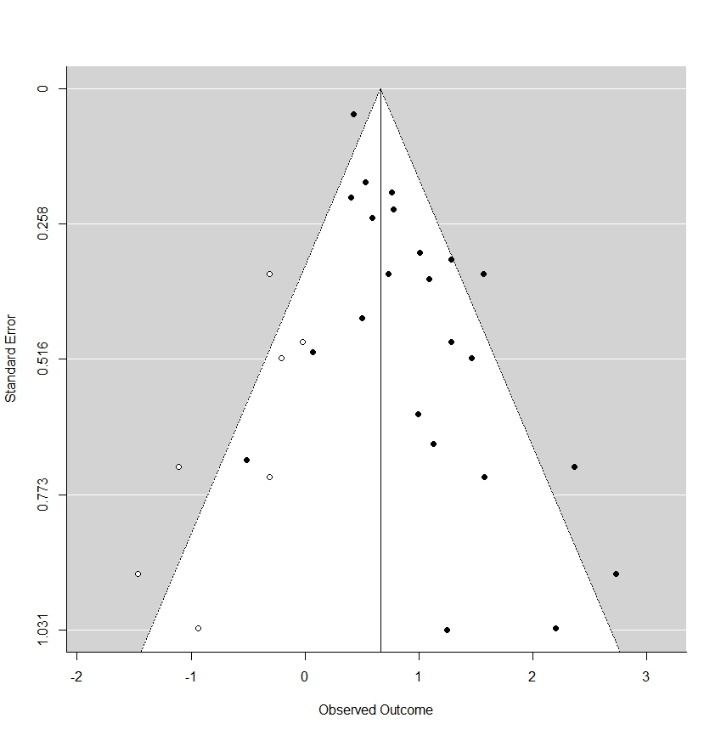

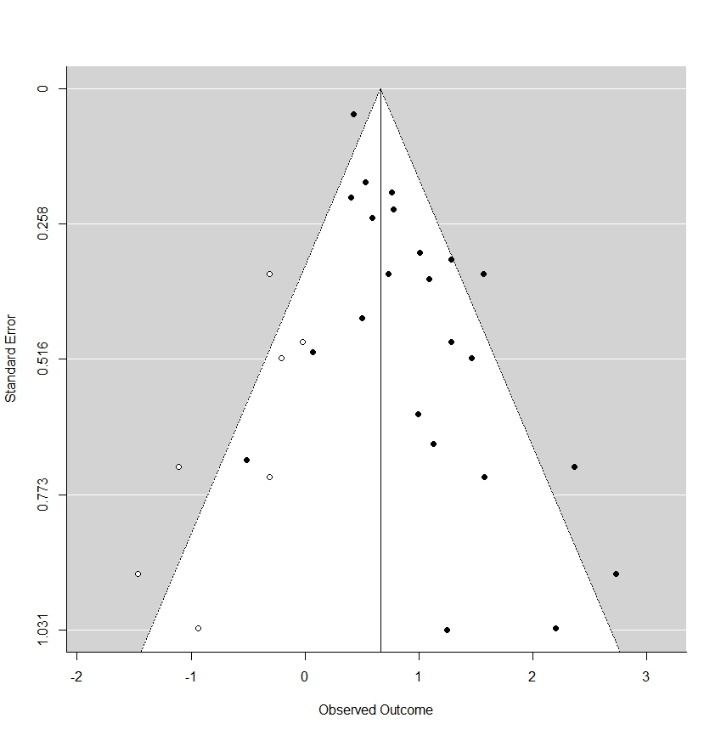

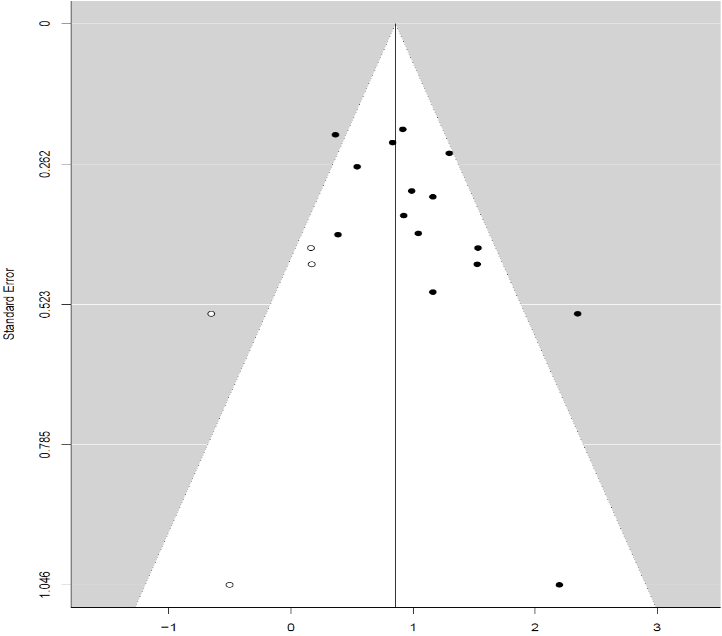
**Figure S3 – Funnel plots for analyses with significant funnel plot asymmetry**

Log hazard ratio

Log hazard ratio


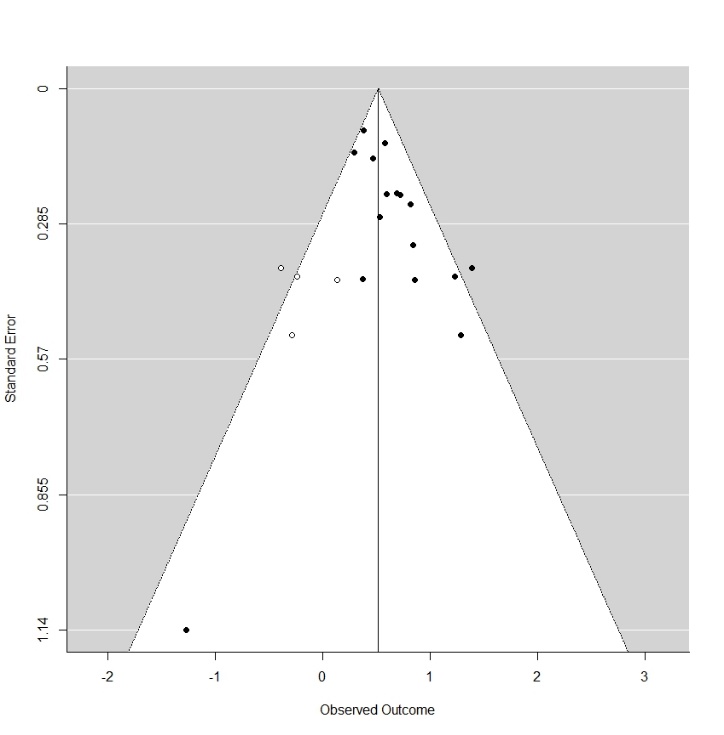


Log hazard ratio

A

B


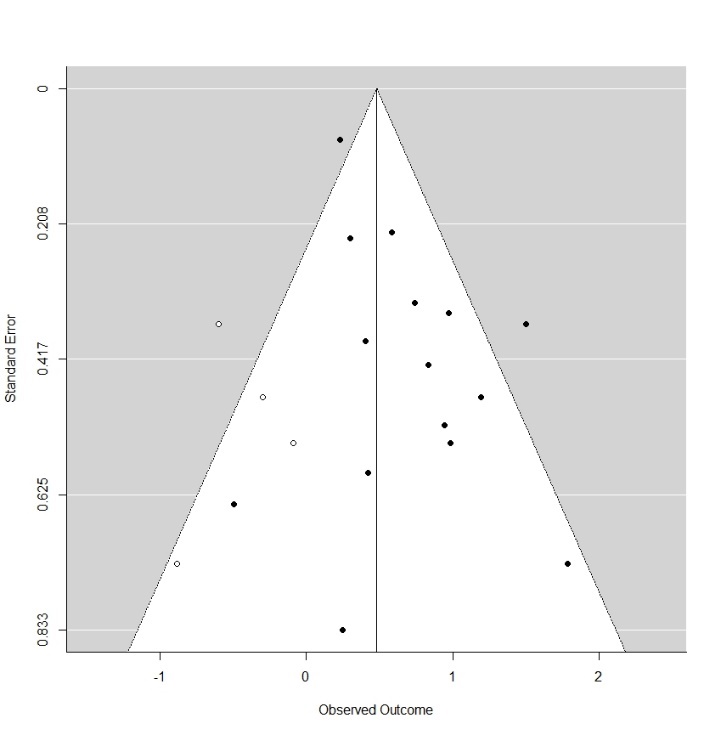


Log hazard ratio

D

C

Funnel plot for the association between white matter hyperintensities on a dichotomous scale and incident haemorrhagic and ischaemic stroke (A), lacunes and incident haemorrhagic and ischaemic stroke (B), cerebral microbleeds and incident haemorrhagic and ischaemic stroke (C), and white matter hyperintensities on a dichotomous scale and all-cause mortality (D). Filled dots correspond to the observed risk estimates, blank dots represent missed studies imputed using the trim and fill test. Diagonal lines indicate the expected 95% confidence intervals around the summary estimate. Hazard ratios are plotted on a natural log scale.

**References**

Akoudad, S., Ikram, M.A., Koudstaal, P.J., Hofman, A., Van Der Lugt, A., Vernooij, M.W., 2013. Cerebral microbleeds and the risk of mortality in the general population. Eur. J. Epidemiol. 28, 815-821.

Akoudad, S., Portegies, M.L.P., Koudstaal, P.J., Hofman, A., Van Der Lugt, A., Ikram, M.A., Vernooij, M.W., 2015. Cerebral microbleeds are associated with an increased risk of stroke: the Rotterdam study. Circulation 132, 509-516.

Akoudad, S., Wolters, F.J., Viswanathan, A., de Bruijn, R.F., van der Lugt, A., Hofman, A., Koudstaal, P.J., Ikram, M.A., Vernooij, M.W., 2016. Association of cerebral microbleeds with cognitive decline and dementia. JAMA Neurol. 73, 934-943.

Altmann-Schneider, I., Trompet, S., de Craen, A.J., van Es, A.C., Jukema, J.W., Stott, D.J., Sattar, N., Westendorp, R.G., van Buchem, M.A., van der Grond, J., 2011. Cerebral microbleeds are predictive of mortality in the elderly. Stroke 42, 638-644.

Andersen, S.D., Larsen, T.B., Gorst-Rasmussen, A., Yavarian, Y., Lip, G.Y., Bach, F.W., 2017. White matter hyperintensities improve ischemic stroke recurrence prediction. Cerebrovasc. Dis. 43, 17-24.

Andersen, S.D., Skjoth, F., Yavarian, Y., Bach, F.W., Lip, G.Y., Larsen, T.B., 2016. Multiple silent lacunes are associated with recurrent ischemic stroke. Cerebrovasc. Dis. 42, 73-80.

Appelros, P., Samuelsson, M., Lindell, D., 2005. Lacunar infarcts: functional and cognitive outcomes at five years in relation to MRI findings. Cerebrovasc. Dis. 20, 34-40.

Benedictus, M.R., Prins, N.D., Goos, J.D., Scheltens, P., Barkhof, F., van der Flier, W.M., 2015. Microbleeds, mortality, and stroke in Alzheimer disease: the MISTRAL study. JAMA Neurol. 72, 539-545.

Bernick, C., Kuller, L., Dulberg, C., Longstreth, W.T., Jr., Manolio, T., Beauchamp, N., Price, T., 2001. Silent MRI infarcts and the risk of future stroke: the cardiovascular health study. Neurology 57, 1222-1229.

Bokura, H., Kobayashi, S., Yamaguchi, S., Iijima, K., Nagai, A., Toyoda, G., Oguro, H., Takahashi, K., 2006. Silent brain infarction and subcortical white matter lesions increase the risk of stroke and mortality: a prospective cohort study. J. Stroke Cerebrovasc. Dis. 15, 57-63.

Bokura, H., Saika, R., Yamaguchi, T., Nagai, A., Oguro, H., Kobayashi, S., Yamaguchi, S., 2011. Microbleeds are associated with subsequent hemorrhagic and ischemic stroke in healthy elderly individuals. Stroke 42, 1867-1871.

Bombois, S., Debette, S., Bruandet, A., Delbeuck, X., Delmaire, C., Leys, D., Pasquier, F., 2008. Vascular subcortical hyperintensities predict conversion to vascular and mixed dementia in MCI patients. Stroke 39, 2046-2051.

Boulanger, J.M., Coutts, S.B., Eliasziw, M., Gagnon, A.J., Simon, J.E., Subramaniam, S., Sohn, C.H., Scott, J., Demchuk, A.M., 2006. Cerebral microhemorrhages predict new disabling or fatal strokes in patients with acute ischemic stroke or transient ischemic attack. Stroke 37, 911-914.

Buyck, J.F., Dufouil, C., Mazoyer, B., Maillard, P., Ducimetiere, P., Alperovitch, A., Bousser, M.G., Kurth, T., Tzourio, C., 2009. Cerebral white matter lesions are associated with the risk of stroke but not with other vascular events: the 3-City Dijon study. Stroke 40, 2327-2331.

Conijn, M.M., Kloppenborg, R.P., Algra, A., Mali, W.P., Kappelle, L.J., Vincken, K.L., van der Graaf, Y., Geerlings, M.I., Group, S.S., 2011. Cerebral small vessel disease and risk of death, ischemic stroke, and cardiac complications in patients with atherosclerotic disease: the second manifestations of arterial disease-magnetic resonance (SMART-MR) study. Stroke 42, 3105-3109.

Debette, S., Beiser, A., Decarli, C., Au, R., Himali, J.J., Kelly-Hayes, M., Romero, J.R., Kase, C.S., Wolf, P.A., Seshadri, S., 2010. Association of MRI markers of vascular brain injury with incident stroke, mild cognitive impairment, dementia, and mortality: the framingham offspring study. Stroke 41, 600-606.

Debette, S., Markus, H.S., 2010. The clinical importance of white matter hyperintensities on brain magnetic resonance imaging: systematic review and meta-analysis. BMJ 341, c3666.

DeCarli, C., Mungas, D., Harvey, D., Reed, B., Weiner, M., Chui, H., Jagust, W., 2004. Memory impairment, but not cerebrovascular disease, predicts progression of MCI to dementia. Neurology 63, 220-227.

Fan, Y.H., Zhang, L., Lam, W.W.M., Mok, V.C.T., Wong, K.S., 2003. Cerebral microbleeds as a risk factor for subsequent intracerebral hemorrhages among patients with acute ischemic stroke. Stroke 34, 2459-2462.

Firbank, M.J., Allan, L.M., Burton, E.J., Barber, R., O'Brien, J.T., Kalaria, R.N., 2012a. Neuroimaging predictors of death and dementia in a cohort of older stroke survivors. J. Neurol. Neurosurg. Psychiatry 83, 263-267.

Firbank, M.J., Teodorczuk, A., van der Flier, W.M., Gouw, A.A., Wallin, A., Erkinjuntti, T., Inzitari, D., Wahlund, L.O., Pantoni, L., Poggesi, A., Pracucci, G., Langhorne, P., O'Brien, J.T., 2012b. Relationship between progression of brain white matter changes and late-life depression: 3-year results from the LADIS study. Br. J. Psychiatry 201, 40-45.

Folsom, A.R., Yatsuya, H., Mosley Jr, T.H., Psaty, B.M., Longstreth Jr, W.T., 2012. Risk of intraparenchymal hemorrhage with magnetic resonance imaging-defined leukoaraiosis and brain infarcts. Annals of Neurology 71, 552-559.

Fu, J.H., Lu, C.Z., Hong, Z., Dong, Q., Luo, Y., Wong, K.S., 2005. Extent of white matter lesions is related to acute subcortical infarcts and predicts further stroke risk in patients with first ever ischaemic stroke. J. Neurol. Neurosurg. Psychiatry 76, 793-796.

Gerdes, V.E., Kwa, V.I., ten Cate, H., Brandjes, D.P., Buller, H.R., Stam, J., 2006. Cerebral white matter lesions predict both ischemic strokes and myocardial infarctions in patients with established atherosclerotic disease. Atherosclerosis 186, 166-172.

Geroldi, C., Rossi, R., Calvagna, C., Testa, C., Bresciani, L., Binetti, G., Zanetti, O., Frisoni, G.B., 2006. Medial temporal atrophy but not memory deficit predicts progression to dementia in patients with mild cognitive impairment. J. Neurol. Neurosurg. Psychiatry 77, 1219-1222.

Gioia, L.C., Tollard, E., Dubuc, V., Lanthier, S., Deschaintre, Y., Chagnon, M., Poppe, A.Y., 2012. Silent ischemic lesions in young adults with first stroke are associated with recurrent stroke. Neurology 79, 1208-1214.

Godin, O., Dufouil, C., Maillard, P., Delcroix, N., Mazoyer, B., Crivello, F., Alperovitch, A., Tzourio, C., 2008. White matter lesions as a predictor of depression in the elderly: the 3C-Dijon study. Biol. Psychiatry 63, 663-669.

Godin, O., Tzourio, C., Rouaud, O., Zhu, Y., Maillard, P., Pasquier, F., Crivello, F., Alperovitch, A., Mazoyer, B., Dufouil, C., 2010. Joint effect of white matter lesions and hippocampal volumes on severity of cognitive decline: the 3C-Dijon MRI study. J. Alzheimers Dis. 20, 453-463.

Gomar, J.J., Bobes-Bascaran, M.T., Conejero-Goldberg, C., Davies, P., Goldberg, T.E., 2011. Utility of combinations of biomarkers, cognitive markers, and risk factors to predict conversion from mild cognitive impairment to Alzheimer disease in patients in the Alzheimer's disease neuroimaging initiative. Arch. Gen. Psychiatry 68, 961-969.

Haji, S., Zubair, A., Planchard, R., Flemming, K., 2015. Clinical relevance of cerebral microbleeds in patients with atrial fibrillation and stroke. Stroke 46.

Henneman, W.J., Sluimer, J.D., Barnes, J., van der Flier, W.M., Sluimer, I.C., Fox, N.C., Scheltens, P., Vrenken, H., Barkhof, F., 2009. Hippocampal atrophy rates in Alzheimer disease: added value over whole brain volume measures. Neurology 72, 999-1007.

Ikram, M.A., Luijendijk, H.J., Vernooij, M.W., Hofman, A., Niessen, W.J., van der Lugt, A., Tiemeier, H., Breteler, M.M., 2010a. Vascular brain disease and depression in the elderly. Epidemiology 21, 78-81.

Ikram, M.A., Vernooij, M.W., Vrooman, H.A., Hofman, A., Breteler, M.M., 2009. Brain tissue volumes and small vessel disease in relation to the risk of mortality. Neurobiol. Aging 30, 450-456.

Ikram, M.A., Vrooman, H.A., Vernooij, M.W., Heijer, T.d., Hofman, A., Niessen, W.J., van der Lugt, A., Koudstaal, P.J., Breteler, M.M.B., 2010b. Brain tissue volumes in relation to cognitive function and risk of dementia. Neurobiol. Aging 31, 378-386.

Imaizumi, T., Inamura, S., Nomura, T., 2015a. Contribution of deep microbleeds to stroke recurrence: differences between patients with past deep intracerebral hemorrhages and lacunar infarctions. J. Stroke Cerebrovasc. Dis. 24, 1855-1864.

Imaizumi, T., Inamura, S., Nomura, T., Kanno, A., Kim, S.N., 2015b. The severity of white matter lesions possibly influences stroke recurrence in patients with histories of lacunar infarctions. J. Stroke Cerebrovasc. Dis. 24, 2154-2160.

Inzitari, D., Pracucci, G., Poggesi, A., Carlucci, G., Barkhof, F., Chabriat, H., Erkinjuntti, T., Fazekas, F., Ferro, J.M., Hennerici, M., Langhorne, P., O'Brien, J., Scheltens, P., Visser, M.C., Wahlund, L.O., Waldemar, G., Wallin, A., Pantoni, L., 2009. Changes in white matter as determinant of global functional decline in older independent outpatients: three year follow-up of LADIS (leukoaraiosis and disability) study cohort. BMJ 339, b2477.

Ishikawa, J., Tamura, Y., Hoshide, S., Eguchi, K., Ishikawa, S., Shimada, K., Kario, K., 2007. Low-grade inflammation is a risk factor for clinical stroke events in addition to silent cerebral infarcts in Japanese older hypertensives: the Jichi medical school ABPM study, wave 1. Stroke 38, 911-917.

Kaffashian, S., Soumare, A., Zhu, Y.C., Mazoyer, B., Debette, S., Tzourio, C., 2016a. Long-term clinical impact of vascular brain lesions on magnetic resonance imaging in older adults in the population. Stroke 47, 2865-2869.

Kaffashian, S., Tzourio, C., Zhu, Y.C., Mazoyer, B., Debette, S., 2016b. Differential effect of white-matter lesions and covert brain infarcts on the risk of ischemic stroke and intracerebral hemorrhage. Stroke 47, 1923-1925.

Kantarci, K., Weigand, S.D., Przybelski, S.A., Shiung, M.M., Whitwell, J.L., Negash, S., Knopman, D.S., Boeve, B.F., O'Brien, P.C., Petersen, R.C., Jack, C.R., Jr., 2009. Risk of dementia in MCI: combined effect of cerebrovascular disease, volumetric MRI, and 1H MRS. Neurology 72, 1519-1525.

Kario, K., Shimada, K., Schwartz, J.E., Matsuo, T., Hoshide, S., Pickering, T.G., 2001. Silent and clinically overt stroke in older Japanese subjects with white-coat and sustained hypertension. J. Am. Coll. Cardiol. 38, 238-245.

Kerber, K.A., Whitman, G.T., Brown, D.L., Baloh, R.W., 2006. Increased risk of death in community-dwelling older people with white matter hyperintensities on MRI. J. Neurol. Sci. 250, 33-38.

Kim, S., Choi, S.H., Lee, Y.M., Kim, M.J., Kim, Y.D., Kim, J.Y., Park, J.H., Myung, W., Na, H.R., Han, H.J., Shim, Y.S., Kim, J.H., Yoon, S.J., Kim, S.Y., Kim, D.K., 2015. Periventricular white matter hyperintensities and the risk of dementia: a CREDOS study. Int. Psychogeriatr. 27, 2069-2077.

Kim, S., Woo, S.Y., Kang, H.S., Lim, S.W., Choi, S.H., Myung, W., Jeong, J.H., Lee, Y., Hong, C.H., Kim, J.H., Na, H., Carroll, B.J., Kim, D.K., 2016. Factors related to prevalence, persistence, and incidence of depressive symptoms in mild cognitive impairment: vascular depression construct. Int. J. Geriatr. Psychiatry 31, 818-826.

Kitagawa, K., Miwa, K., Yagita, Y., Okazaki, S., Sakaguchi, M., Mochizuki, H., 2015. Association between carotid stenosis or lacunar infarction and incident dementia in patients with vascular risk factors. Eur. J. Neurol. 22, 187-192.

Kobayashi, S., Okada, K., Koide, H., Bokura, H., Yamaguchi, S., 1997. Subcortical silent brain infarction as a risk factor for clinical stroke. Stroke 28, 1932-1939.

Korf, E.S., Wahlund, L.O., Visser, P.J., Scheltens, P., 2004. Medial temporal lobe atrophy on MRI predicts dementia in patients with mild cognitive impairment. Neurology 63, 94-100.

Kuller, L.H., 2003. Risk factors for dementia in the cardiovascular health study cognition study. Rev. Neurol. 37, 122-126.

Kuller, L.H., Arnold, A.M., Longstreth, W.T., Jr., Manolio, T.A., O'Leary, D.H., Burke, G.L., Fried, L.P., Newman, A.B., 2007. White matter grade and ventricular volume on brain MRI as markers of longevity in the cardiovascular health study. Neurobiol. Aging 28, 1307-1315.

Kuller, L.H., Longstreth, W.T., Jr., Arnold, A.M., Bernick, C., Bryan, R.N., Beauchamp, N.J., 2004. White matter hyperintensity on cranial magnetic resonance imaging: a predictor of stroke. Stroke 35, 1821-1825.

Kumral, E., Gulluoglu, H., Alakbarova, N., Karaman, B., Deveci, E.E., Bayramov, A., Evyapan, D., Gokcay, F., Orman, M., 2015. Association of leukoaraiosis with stroke recurrence within 5 years after initial stroke. J. Stroke Cerebrovasc. Dis. 24, 573-582.

Kwa, V.I.H., Algra, A., Brundel, M., Bouvy, W., Kappelle, L.J., 2012. Microbleeds as a predictor of intracerebral hemorrhage in patients receiving oral antithrombotic drugs after a TIA or minor ischemic stroke: a prospective cohort study in Western outpatients. Cerebrovasc. Dis. 33, 779-780.

Lavretsky, H., Zheng, L., Weiner, M.W., Mungas, D., Reed, B., Kramer, J.H., Jagust, W., Chui, H., Mack, W.J., 2010. Association of depressed mood and mortality in older adults with and without cognitive impairment in a prospective naturalistic study. Am. J. Psychiatry 167, 589-597.

Levy, R.M., Steffens, D.C., McQuoid, D.R., Provenzale, J.M., MacFall, J.R., Krishnan, K.R., 2003. MRI lesion severity and mortality in geriatric depression. Am. J. Geriatr. Psychiatry 11, 678-682.

Lopez, O.L., Klunk, W.E., Mathis, C., Coleman, R.L., Price, J., Becker, J.T., Aizenstein, H.J., Snitz, B., Cohen, A., Ikonomovic, M., McDade, E., DeKosky, S.T., Weissfeld, L., Kuller, L.H., 2014. Amyloid, neurodegeneration, and small vessel disease as predictors of dementia in the oldest-old. Neurology 83, 1804-1811.

Meguro, K., Ishii, H., Kasuya, M., Akanuma, K., Meguro, M., Kasai, M., Lee, E., Hashimoto, R., Yamaguchi, S., Asada, T., 2007. Incidence of dementia and associated risk factors in Japan: the Osaki-Tajiri project. J. Neurol. Sci. 260, 175-182.

Melkas, S., Sibolt, G., Oksala, N.K.J., Putaala, J., Pohjasvaara, T., Kaste, M., Karhunen, P.J., Erkinjuntti, T., 2012. Extensive white matter changes predict stroke recurrence up to 5 years after a first-ever ischemic stroke. Cerebrovasc. Dis. 34, 191-198.

Miwa, K., Tanaka, M., Okazaki, S., Yagita, Y., Sakaguchi, M., Mochizuki, H., Kitagawa, K., 2014. Multiple or mixed cerebral microbleeds and dementia in patients with vascular risk factors. Neurology 83, 646-653.

Mok, V.C., Lau, A.Y., Wong, A., Lam, W.W., Chan, A., Leung, H., Wong, E., Soo, Y., Leung, T., Wong, L.K., 2009. Long-term prognosis of Chinese patients with a lacunar infarct associated with small vessel disease: a five-year longitudinal study. Int. J. Stroke 4, 81-88.

Naganuma, T., Takemoto, Y., Shoji, T., Ishimura, E., Okamura, M., Nakatani, T., 2015. Cerebral microbleeds predict intracerebral hemorrhage in hemodialysis patients. Stroke 46, 2107-2112.

Naganuma, T., Takemoto, Y., Shoji, T., Shima, H., Ishimura, E., Okamura, M., Nakatani, T., 2013. Cerebral white matter hyperintensity predicts cardiovascular events in haemodialysis patients. Nephrology 18, 676-681.

Naka, H., Nomura, E., Takahashi, T., Wakabayashi, S., Mimori, Y., Kajikawa, H., Kohriyama, T., Matsumoto, M., 2006. Combinations of the presence or absence of cerebral microbleeds and advanced white matter hyperintensity as predictors of subsequent stroke types. Am. J. Neuroradiol. 27, 830-835.

Nishikawa, T., Ueba, T., Kajiwara, M., Fujisawa, I., Miyamatsu, N., Yamashita, K., 2009. Cerebral microbleeds predict first-ever symptomatic cerebrovascular events. Clin. Neurol. Neurosurg. 111, 825-828.

Ntaios, G., Lip, G.Y.H., Lambrou, D., Papavasileiou, V., Manios, E., Milionis, H., Spengos, K., Makaritsis, K., Vemmos, K., 2015. Leukoaraiosis and stroke recurrence risk in patients with and without atrial fibrillation. Neurology 84, 1213-1219.

Oksala, N.K., Oksala, A., Pohjasvaara, T., Vataja, R., Kaste, M., Karhunen, P.J., Erkinjuntti, T., 2009. Age related white matter changes predict stroke death in long term follow-up. J. Neurol. Neurosurg. Psychiatry 80, 762-766.

Park, J.H., Lee, S.B., Lee, J.J., Yoon, J.C., Han, J.W., Kim, T.H., Jeong, H.G., Newhouse, P.A., Taylor, W.D., Kim, J.H., Woo, J.I., Kim, K.W., 2015. Epidemiology of MRI-defined vascular depression: a longitudinal, community-based study in Korean elders. J. Affect. Disord. 180, 200-206.

Perez, H.C.S., 2013. Silent brain infarcts: a cause of depression in the elderly? Psychiatry Res. 211, 180-182.

Poels, M.M., Steyerberg, E.W., Wieberdink, R.G., Hofman, A., Koudstaal, P.J., Ikram, M.A., Breteler, M.M., 2012. Assessment of cerebral small vessel disease predicts individual stroke risk. J. Neurol. Neurosurg. Psychiatry 83, 1174-1179.

Prasad, K., Wiryasaputra, L., Ng, A., Kandiah, N., 2011. White matter disease independently predicts progression from mild cognitive impairment to Alzheimer's disease in a clinic cohort. Dement. Geriatr. Cogn. Disord. 31, 431-434.

Prins, N.D., van der Flier, W.M., Brashear, H.R., Knol, D.L., van de Pol, L.A., Barkhof, F., Scheltens, P., 2013. Predictors of progression from mild cognitive impairment to dementia in the placebo-arm of a clinical trial population. J. Alzheimers Dis. 36, 79-85.

Prins, N.D., van Dijk, E.J., den Heijer, T., Vermeer, S.E., Koudstaal, P.J., Oudkerk, M., Hofman, A., Breteler, M.M., 2004. Cerebral white matter lesions and the risk of dementia. Arch. Neurol. 61, 1531-1534.

Putaala, J., Haapaniemi, E., Kurkinen, M., Salonen, O., Kaste, M., Tatlisumak, T., 2011. Silent brain infarcts, leukoaraiosis, and long-term prognosis in young ischemic stroke patients. Neurology 76, 1742-1749.

Qiu, W.Q., Himali, J.J., Wolf, P.A., DeCarli, D.C., Beiser, A., Au, R., 2016. Effects of white matter integrity and brain volumes on late life depression in the framingham heart study. Int. J. Geriatr. Psychiatry.

Rosano, C., Aizenstein, H.J., Wu, M., Newman, A.B., Becker, J.T., Lopez, O.L., Kuller, L.H., 2007. Focal atrophy and cerebrovascular disease increase dementia risk among cognitively normal older adults. J. Neuroimaging 17, 148-155.

Sluimer, J.D., van der Flier, W.M., Karas, G.B., Fox, N.C., Scheltens, P., Barkhof, F., Vrenken, H., 2008. Whole-brain atrophy rate and cognitive decline: longitudinal MR study of memory clinic patients. Radiology 248, 590-598.

Smith, E.E., Egorova, S., Blacker, D., Killiany, R.J., Muzikansky, A., Dickerson, B.C., Tanzi, R.E., Albert, M.S., Greenberg, S.M., Guttmann, C.R., 2008. Magnetic resonance imaging white matter hyperintensities and brain volume in the prediction of mild cognitive impairment and dementia. Arch. Neurol. 65, 94-100.

Smith, E.E., Gurol, M.E., Eng, J.A., Engel, C.R., Nguyen, T.N., Rosand, J., Greenberg, S.M., 2004. White matter lesions, cognition, and recurrent hemorrhage in lobar intracerebral hemorrhage. Neurology 63, 1606-1612.

Soo, Y.O., Yang, S.R., Lam, W.W., Wong, A., Fan, Y.H., Leung, H.H., Chan, A.Y., Leung, C., Leung, T.W., Wong, L.K., 2008. Risk vs benefit of anti-thrombotic therapy in ischaemic stroke patients with cerebral microbleeds. J. Neurol. 255, 1679-1686.

Staekenborg, S.S., Koedam, E.L., Henneman, W.J., Stokman, P., Barkhof, F., Scheltens, P., van der Flier, W.M., 2009. Progression of mild cognitive impairment to dementia: contribution of cerebrovascular disease compared with medial temporal lobe atrophy. Stroke 40, 1269-1274.

Staff, R.T., Murray, A.D., Ahearn, T., Salarirad, S., Mowat, D., Starr, J.M., Deary, I.J., Lemmon, H., Whalley, L.J., 2010. Brain volume and survival from age 78 to 85: the contribution of Alzheimer-type magnetic resonance imaging findings. J. Am. Geriatr. Soc. 58, 688-695.

Steffens, D.C., Krishnan, K.R., Crump, C., Burke, G.L., 2002a. Cerebrovascular disease and evolution of depressive symptoms in the cardiovascular health study. Stroke 33, 1636-1644.

Steffens, D.C., Payne, M.E., Greenberg, D.L., Byrum, C.E., Welsh-Bohmer, K.A., Wagner, H.R., MacFall, J.R., 2002b. Hippocampal volume and incident dementia in geriatric depression. Am. J. Geriatr. Psychiatry 10, 62-71.

Steffens, D.C., Potter, G.G., McQuoid, D.R., Macfall, J.R., Payne, M.E., Burke, J.R., Plassman, B.L., Welsh-Bohmer, K.A., 2007. Longitudinal magnetic resonance imaging vascular changes, apolipoprotein e genotype, and development of dementia in the neurocognitive outcomes of depression in the elderly study. Am. J. Geriatr. Psychiatry 15, 839-849.

Stephan, B.C.M., Tzourio, C., Auriacombe, S., Amieva, H., Dufouil, C., Alperovitch, A., Kurth, T., 2015. Usefulness of data from magnetic resonance imaging to improve prediction of dementia: population based cohort study. BMJ 350.

Stoub, T.R., DeToledo-Morrell, L., Dickerson, B.C., 2014. Parahippocampal white matter volume predicts Alzheimer's disease risk in cognitively normal old adults. Neurobiol. Aging 35, 1855-1861.

Tapiola, T., Pennanen, C., Tapiola, M., Tervo, S., Kivipelto, M., Hanninen, T., Pihlajamaki, M., Laakso, M.P., Hallikainen, M., Hamalainen, A., Vanhanen, M., Helkala, E.L., Vanninen, R., Nissinen, A., Rossi, R., Frisoni, G.B., Soininen, H., 2008. MRI of hippocampus and entorhinal cortex in mild cognitive impairment: a follow-up study. Neurobiol. Aging 29, 31-38.

Teodorczuk, A., Firbank, M.J., Pantoni, L., Poggesi, A., Erkinjuntti, T., Wallin, A., Wahlund, L.O., Scheltens, P., Waldemar, G., Schrotter, G., Ferro, J.M., Chabriat, H., Bazner, H., Visser, M., Inzitari, D., O'Brien, J.T., Group, L., 2010. Relationship between baseline white-matter changes and development of late-life depressive symptoms: 3-year results from the LADIS study. Psychol. Med. 40, 603-610.

Teodorczuk, A., O'Brien, J.T., Firbank, M.J., Pantoni, L., Poggesi, A., Erkinjuntti, T., Wallin, A., Wahlund, L.O., Gouw, A., Waldemar, G., Schmidt, R., Ferro, J.M., Chabriat, H., Bazner, H., Inzitari, D., 2007. White matter changes and late-life depressive symptoms: longitudinal study. Br. J. Psychiatry 191, 212-217.

Thijs, V., Lemmens, R., Schoofs, C., Gorner, A., Van Damme, P., Schrooten, M., Demaerel, P., 2010. Microbleeds and the risk of recurrent stroke. Stroke 41, 2005-2009.

van der Holst, H.M., van Uden, I.W., Tuladhar, A.M., de Laat, K.F., van Norden, A.G., Norris, D.G., van Dijk, E.J., Rutten-Jacobs, L.C., de Leeuw, F.E., 2016. Factors associated with 8-Year mortality in older patients with cerebral small vessel disease: the Radboud university Nijmegen diffusion tensor and magnetic resonance cohort (RUN DMC) study. JAMA Neurol. 73, 402-409.

van der Veen, P.H., Muller, M., Vincken, K.L., Mali, W.P., van der Graaf, Y., Geerlings, M.I., 2014. Brain volumes and risk of cardiovascular events and mortality. The SMART-MR study. Neurobiol. Aging 35, 1624-1631.

van Sloten, T.T., Sigurdsson, S., van Buchem, M.A., Phillips, C.L., Jonsson, P.V., Ding, J., Schram, M.T., Harris, T.B., Gudnason, V., Launer, L.J., 2015. Cerebral small vessel disease and association with higher incidence of depressive symptoms in a general elderly population: the AGES-Reykjavik study. Am. J. Psychiatry 172, 570-578.

van Straaten, E.C., Harvey, D., Scheltens, P., Barkhof, F., Petersen, R.C., Thal, L.J., Jack, C.R., Jr., DeCarli, C., 2008. Periventricular white matter hyperintensities increase the likelihood of progression from amnestic mild cognitive impairment to dementia. J. Neurol. 255, 1302-1308.

van Uden, I.W., van der Holst, H.M., Tuladhar, A.M., van Norden, A.G., de Laat, K.F., Rutten-Jacobs, L.C., Norris, D.G., Claassen, J.A., van Dijk, E.J., Kessels, R.P., de Leeuw, F.E., 2015. White matter and hippocampal volume predict the risk of dementia in patients with cerebral small vessel disease: the RUN DMC study. J. Alzheimers Dis. 49, 863-873.

Verdelho, A., Madureira, S., Moleiro, C., Ferro, J.M., Santos, C.O., Erkinjuntti, T., Pantoni, L., Fazekas, F., Visser, M., Waldemar, G., Wallin, A., Hennerici, M., Inzitari, D., 2010. White matter changes and diabetes predict cognitive decline in the elderly: the LADIS study. Neurology 75, 160-167.

Vermeer, S.E., Prins, N.D., Den Heijer, T., Hofman, A., Koudstaal, P.J., Breteler, M.M.B., 2003. Silent brain infarcts and the risk of dementia and cognitive decline. N. Engl. J. Med. 348, 1215-1222.

Versluis, C.E., van der Mast, R.C., van Buchem, M.A., Bollen, E.L., Blauw, G.J., Eekhof, J.A., van der Wee, N.J., de Craen, A.J., 2006. Progression of cerebral white matter lesions is not associated with development of depressive symptoms in elderly subjects at risk of cardiovascular disease: the PROSPER study. Int. J. Geriatr. Psychiatry 21, 375-381.

Weber, R., Weimar, C., Wanke, I., Moller-Hartmann, C., Gizewski, E.R., Blatchford, J., Hermansson, K., Demchuk, A.M., Forsting, M., Sacco, R.L., Saver, J.L., Warach, S., Diener, H.C., Diehl, A., Group, P.R.I.S., 2012. Risk of recurrent stroke in patients with silent brain infarction in the prevention regimen for effectively avoiding second strokes (PRoFESS) imaging substudy. Stroke 43, 350-355.

Weinstein, G., Beiser, A.S., Decarli, C., Au, R., Wolf, P.A., Seshadri, S., 2013. Brain imaging and cognitive predictors of stroke and Alzheimer disease in the framingham heart study. Stroke 44, 2787-2794.

Windham, B.G., Deere, B., Griswold, M.E., Wang, W., Bezerra, D.C., Shibata, D., Butler, K., Knopman, D., Gottesman, R.F., Heiss, G., Mosley, T.H., 2015. Small brain lesions and incident stroke and mortality: a cohort study. Ann. Intern. Med. 163, 22-31.

Yamamoto, Y., Akiguchi, I., Oiwa, K., Hayashi, M., Kasai, T., Ozasa, K., 2002. Twenty-four-hour blood pressure and MRI as predictive factors for different outcomes in patients with lacunar infarct. Stroke 33, 297-305.

Yamauchi, H., Fukuda, H., Oyanagi, C., 2002. Significance of white matter high intensity lesions as a predictor of stroke from arteriolosclerosis. J. Neurol. Neurosurg. Psychiatry 72, 576-582.

Zhu, Y.C., Dufouil, C., Soumare, A., Mazoyer, B., Chabriat, H., Tzourio, C., 2010. High degree of dilated Virchow-Robin spaces on MRI is associated with increased risk of dementia. J. Alzheimers Dis. 22, 663-672.
